# Class IIa HDACs forced degradation allows resensitization of oxaliplatin‐resistant FBXW7‐mutated colorectal cancer

**DOI:** 10.1002/1878-0261.70152

**Published:** 2025-10-31

**Authors:** Vanessa Tolotto, Nicolò Gualandi, Ylenia Cortolezzis, Raffaella Picco, Monica Colitti, Francesca D'Este, Mariachiara Gani, Wayne W. Hancock, Giovanni Terrosu, Cristina Degrassi, Francesca Agostini, Claudio Brancolini, Luigi E. Xodo, Eros Di Giorgio

**Affiliations:** ^1^ Laboratory of Biochemistry, Department of Medicine Università Degli Studi di Udine Italy; ^2^ Dipartimento di Scienze Agroalimentari, Ambientali e Animali Università Degli Studi di Udine Italy; ^3^ Division of Transplant Immunology, Department of Pathology and Laboratory Medicine, Children's Hospital of Philadelphia and Perelman School of Medicine University of Pennsylvania Philadelphia PA USA; ^4^ Department of Medicine, Institute for Biomedicine Università Degli Studi di Udine Italy; ^5^ MTTLab Trieste Italy; ^6^ Laboratory of Epigenomics, Department of Medicine Università Degli Studi di Udine Italy

**Keywords:** colon cancer, epigenetic landscape, HDAC4, oxaliplatin, PROTAC, super‐enhancer

## Abstract

Epigenetic plasticity and large‐scale chromatin remodeling characterize tumor evolution and the emergence of subclones resistant to conventional therapies. Catalytically inactive class IIa HDACs (HDAC4, HDAC5, HDAC7, HDAC9) control the targeted recruitment of chromatin remodeling complexes, making them attractive therapeutic targets in oncology. In this study, we found that HDAC4 is degraded by the proteasome in cancer cells with impaired DNA repair by homologous recombination and after oxaliplatin (OXPT) treatment. Genetic screening identified FBXW7 as the E3 ligase responsible for HDAC4 degradation. FBXW7 loss‐of‐function mutations are frequently found in patients with colorectal cancer (CRC) and were found associated with the development of resistance to OXPT. Forced degradation of Class IIa HDACs using a PROTAC‐based compound restored OXPT sensitivity in FBXW7‐mutated CRC cells, patient‐derived organoids (PDOs), and mice. Mechanistically, removal of HDAC4 in FBXW7‐mutated CRC treated with OXPT recreated an epigenetic state comparable to OXPT‐sensitive cells. Furthermore, patient profiling based on the epigenetic state of the super‐enhancers controlled by HDAC4 successfully identified *a priori* CRC patients resistant to platinum. This study supports HDAC4 as a key mediator of oxaliplatin resistance in FBXW7‐mutated CRC and highlights the remodeling of a well‐defined super‐enhancer repertoire as part of the process of OXPT resensitization.

## Graphical abstract

HDAC4 is degraded by the E3 ligase FBXW7. In colorectal cancer, FBXW7 mutations prevent HDAC4 degradation, leading to oxaliplatin resistance. Forced degradation of HDAC4 using a PROTAC compound restores drug sensitivity by resetting the super‐enhancer landscape, reprogramming the epigenetic state of FBXW7‐mutated cells to resemble oxaliplatin‐responsive profiles.

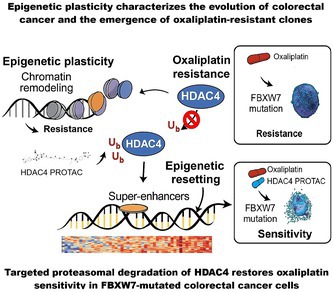

**Abbreviations**
CRC: colorectal cancer
DSBs: double‐strand breaks
FOLFOX: Folinic acid, fluorouracil 5‐FU, oxaliplatin
GI: growth inhibition
GSEA: gene set enrichment analysis
HDAC: histone deacetylase
HR: homologous recombination
HRR: homologous‐recombination repair
KO: knockout
LOF: loss‐of‐function
NHEJ: nonhomologous end joining
OXPHOS: oxidative phosphorylation
OXPT: oxaliplatin
PDOs: patient‐derived organoids
PI: propidium iodide
PROTAC: proteolysis‐targeting Chimera
SE: super‐enhancer
STS: staurosporine
TAD: topological‐associated domain
TMA: tissue microarray
TMRM: tetramethylrhodamine methyl ester perchlorate
TV: tumor volumes
UPS: ubiquitin–proteosome system
VHL: Von Hippel–Lindau
WT: wild‐type

## 1 Introduction

Oxaliplatin (OXPT)‐based chemotherapy and oxaliplatin‐containing chemotherapy are recommended adjuvant treatments and first‐line treatments, respectively, for stage III colorectal cancer (CRC) [1, 2] and metastatic CRC [3]. Resistance mechanism to OXPT include limited drug uptake, increased drug efflux or detoxification, increased DNA repair, and decreased activation of the apoptotic cascade [4]. The lack of a clear genetic link with the acquisition of resistance to OXPT suggests the existence of an epigenetic mechanism underlying it [5, 6, 7, 8]. Importantly, nonselective HDAC inhibitors [9, 10, 11] proved effective in resensitizing resistant CRC cells to OXPT, suggesting a possible role played by histone deacetylases in mediating platinum sensitivity.

Class IIa HDACs are catalytically inactive acetyl‐lysine readers that supervise the acetylation state of histones by recruiting protein complexes involved in chromatin remodeling [12, 13] on selected chromatin domains [12, 14]. Recently, specific activities of these deacetylases have been reported in the regulation of enhancer clusters known as super‐enhancers (SEs), controlling the transcriptional circuits activated by master transcription factors [14, 15, 16, 17, 18, 19].

Recurrent somatic changing of chromatin accessibility [20], mainly involving a remodeling of the enhancerome [21], have been reported in CRC and successfully distinguish between malignant and benign lesions [20]. Despite this important evidence, epigenome modulation in chemoresistant CRC subclones is still poorly characterized.

Here, we identified FBXW7 as the E3 ligase responsible for HDAC4 degradation in OXPT‐treated tumor cells. Loss‐of‐function (LOF) mutations of FBXW7 are frequent in CRC [22] and correlate with the development of resistance to OXPT [22, 23, 24, 25, 26, 27]. In this manuscript, we investigated whether epigenetic modifications at the chromatin level support OXPT resistance in FBXW7‐depleted or mutated CRC models and how their resetting with a PROTAC compound aimed at degrading class IIa HDACs was functional in restoring sensitivity.

## 2 Materials and methods

### 2.1 Cell culture and treatments

SK‐LMS‐1 (RRID:CVCL_0628), SW837 (RRID:CVCL_1729), LoVo (RRID:CVCL_0399), SW620 (CVCL_0547), HT‐29 (RRID:CVCL_0320), NIH‐3T3 (RRID:CVCL_0594), Phoenix‐Ampho (RRID:CVCL_H716), and HEK293 (RRID:CVCL_0045) were from ATCC (Virginia, USA). HCT‐116 (RRID:CVCL_0291) WT or *FBXW7* ^−/−^ were obtained from Ubigene (Guangzhou, China) and validated by Sanger sequencing. U2OS (RRID:CVCL_0042) EJ5 and TRI‐DR [28], MEF (RRID:CVCL_C1M8) WT or GSK3β^−/−^ [29], were previously characterized and grown in DMEM (Euroclone, Milan, Italy) supplemented with 10% FBS, penicillin/streptomycin, and 2 mm l‐glutamine (Euroclone) at 37 °C, 5% CO_2_. Parental cell lines were STR profiled and authenticated for this paper (Eurofins, Ebersberg, Germany). Cells were routinely screened for mycoplasma contamination, and all experiments were performed with mycoplasma‐free cells. For GI_50_ calculation, cells were seeded in 96‐well plates and treated 24 h later with increasing concentrations of the compounds under evaluation. After 72 h, cell viability was assessed using the resazurin assay [15], and the conversion of resazurin to resorufin was measured using a BioTek plate reader (US).

The following chemicals were used: 1 μm Doxycycline, 2.5 μm MG132, 20 μm RI‐1, 1 μm staurosporine, LiCl, iodoacetamide, Berzosertib M6620 (all from Sigma‐Aldrich, Darmstadt, Germany), 1 μm PS‐341 (LC Laboratories, Woburn, MA, USA), 20 μm Scr7 and 20 μm Mirin (Tocris, Bristol, UK), Oxaliplatin and 5‐Fluorouracil (CliniSciences, Rome, Italy) at the indicated concentrations. PROTAC #11 targeting HDAC4 [30] was kindly provided by Dr Celia Dominguez and used at the indicated concentrations. In all the experiments, the condition defined as ‘untreated’ referred to cells or PDOs receiving quantities comparable to solvent treatments in which the molecules whose activity is being tested were resuspended: water for OXPT, DMSO for PROTAC #11, and STS.

### 2.2 CRC organoids culture

Patient‐derived CRC organoids PDM96 (HCM‐CSHL‐0143‐C20) and PDM257 (HCM‐CSHL‐0248‐C19) were from ATCC (Virginia, USA). Organoids were embedded in a 1 : 1 mixture of Cultrex (R&D Systems, Minneapolis, MN, USA) and ECM Gel (Sigma‐Aldrich) and grown in Advanced DMEM/F12 (Gibco, USA) supplemented with 2 mm l‐glutamine (Euroclone), 10 mm Hepes (Thermo Fisher Scientific, Waltham, MA, USA), 1× B‐27 supplement (Gibco, New York, NY, USA), 10 mm Nicotinamide (Sigma‐Aldrich), 1.25 mm *N*‐acetylcysteine (Sigma‐Aldrich), 50 ng·mL^−1^ EGF, 100 ng·mL^−1^ Noggin, and 500 ng·mL^−1^ R‐spondin recombinant proteins (all from PeproTech, London, UK), 3 μm SB202190 (Sigma‐Aldrich). Y27632 analogue Pro‐survival Compound (Sigma‐Aldrich) was added for the first days of organoids culture after thawing.

### 2.3 Plasmid construction, transfection, retroviral and lentiviral infection, silencing

pRK5 HA Ubiquitin WT (#17608), pRK5 HA Ubiquitin K48R (#17605), pLIX 403 (#41395), pQCXIP X2 DEST (w270‐1) (#17474), pDONR223 FBXW7 WT (#81795), pDONR223 FBXW7 p.R505C (#81507), pCMV4a‐Flag‐c‐Myc (#102625), pLS‐mP (#81225), and pLS‐SV40‐mP‐EGFP (#137724) were obtained from Addgene (Watertown, MA, USA).

pLKO Puro shHDAC4 1 (TRCN0000314667) and 2 (TRCN000004832), pLKO Puro shAMFR (TRCN0000003375), pLKO Puro shFAF2 (TRCN0000004405), pLKO Puro shFBXW7 1 (TRCN0000006555), pLKO Puro shMARCH5 (TRCN0000037015), pLKO Puro shMARCH6 (TRCN0000232719), pLKO Puro shRNF185 (TRCN0000034054), pLKO Puro shMGRN1 (TRCN0000033790), pLKO Puro shSMURF2 (TRCN0000010792), pLKO Puro shTRIM29 (TRCN0000016350), pLKO Puro shRFFL (TRCN0000034077), pLKO Puro shSPOP (TRCN0000139181), pLKO Puro shFBXO28 (TRCN0000180818), pLKO Puro shFBXO38 (TRCN0000133912), pLKO Puro shRHOBTB3 (TRCN0000048644), pLKO Puro shGSK3β (TRCN0000010552), pLKO Puro shBRCA1_1 (TRCN0000010305), and pLKO Puro shBRCA1_2 (TRCN0000009823) were obtained from Sigma‐Aldrich.

pFLAG expressing HDAC4 WT, HDAC4 S298D, and HDAC4 deletions were previously described [12]. HDAC4 S215 and S219 mutants were generated by site‐directed mutagenesis (Agilent, Santa Clara, CA, USA) starting from pFLAG HDAC4. pEGFP C1 FBXW7 WT or R505C mutants were obtained by subcloning FBXW7 ORF restricted BglII/EcoRI from pDONR223 into pEGFP C1 linearized with BglII/EcoRI. pLIX 403 FBXW7 WT and pQCXIP X2 DEST FBXW7 R505C constructs were obtained by L/R recombination following the manufacturer instructions (Gateway LR Clonase II Enzyme mix, Thermo Fisher Scientific), using pDONR223 FBXW7 WT or pDONR223 FBXW7 R505C as donor vectors and pLIX 403 or pQCXIP X2 DEST (w270‐1) as acceptors, respectively.

pLS‐NR4A2‐EGFP and pLS‐RNF43‐EGFP were generated by PCR‐restriction (XbaI, SbfI) and contain the regulative element of NR4A2 (hg38, chr2:156333669‐156334207), and RNF43 (hg38, chr17:58402341‐58402769), respectively.

Transfections, viral infections, and siRNA delivery were done as previously described [31].

For the infection of PDM‐96 and PDM‐257 organoids, single cell suspensions were obtained by mechanical dissociation of the extracellular matrix and subsequent digestion with trypsin. Infection was performed in suspension by mixing 1 : 1 medium containing virion with basal Advanced DMEM/F12 at 37 °C, 5% CO_2_. After 6 h of infection, cells were embedded in extracellular matrix; after 48 h, a second round of infection of the same cells was performed by repeating the same procedure.

For silencing, cells were seeded in a 6‐well plate and transfected with 148 pmol of siRNA or 46 pmol of esiRNA and 5.5 μL per well of Lipofectamine 3000 (Life Technologies, Carlsbad, CA, USA). The following siRNAs were used: FBXW7 (ACAGGACAGUGUUUACAAA), MARCH5 (GGUUGUAGGCCAUAAAGAA), MARCH6 (UCAUAGAUCUCGUCGCUUA), RNF185 (GUGGCUUCCAGAUGUCUUU), all from Sigma‐Aldrich.

The following esiRNA was used to minimize off‐targets and confirm data obtained with siRNAs: HDAC4 (EHU136981), RAD51 (EHU045521), RAD52 (EHU139731), FBXW7 (EHU075951), Control (EHURLUC), all from Sigma‐Aldrich.

### 2.4 RNA extraction, quantitative qRT‐PCR, and RNA‐seq

RNA extraction and quantitative qRT‐PCR were performed as previously described [31]. Data were analyzed by comparative threshold cycle (delta delta Ct) using HPRT and GAPDH as normalizers. The used primers are listed in Table S7.

For RNA‐seq, total RNA was treated with DNAse I (NEB, Ipswich, MA, USA) and purified with RNA Clean & Concentrator (Zymo Research, Irvine, CA, USA). RNA‐seq library preparation and sequencing were performed at Azenta Genewiz (Leipzig, Germany) following Illumina specifications for polyA‐enriched mRNA. The analysis of the RNA‐seq data was performed as previously described [32]. Briefly, quality control for raw sequencing reads was performed with programs fastqc (v0.11.9) and multiqc (v1.09). Transcript quantification was conducted with salmon (v1.4.0) on human transcriptome GRCh38 Ensembl version 100 (gene set patch level 13). Transcript quantifications were imported into r (v4.0.3) running bioconductor (v3.11) for downstream analysis with tximeta (v1.6.3) and summarized at the gene level. Principal component analysis was carried out with the plotPCA function from the deseq2 package (v1.28.1). Genes with raw count means < 64 between each condition replicates were removed from the analysis. Differential expression analysis was performed using deseq2 with Wald test for significance. We adjusted for multiple hypothesis testing by employing Benjamini–Hochberg correction at a false discovery rate (FDR) of 0.05. Genes reported significantly by deseq2 with an absolute fold change > 1.5 were considered as differentially expressed. Genes were annotated with package annotationhub (v2.20.2) utilizing Ensembl annotation 100 data. Plots were generated with ggplot2 (v3.3.3). Venn diagrams were created with venndiagram (v1.6.20). Functional annotation was performed on KEGG, Reactome slimGO and Gene Ontology databases with clusterprofiler (v3.16.1), reactomepa (v1.32.0), and enrichr [33].

### 2.5 Immunofluorescence, time‐lapse videomicroscopy and immunoblotting

For 3D culture immunofluorescence, CRC organoids were seeded into 8‐well chambered coverslips (Ibidi, Grafelfing, Germany) and treated as indicated after 3 days from seeding. Organoids were fixed with 3% paraformaldehyde for 20 min and permeabilized with 0.3% Triton X‐100 for 5 min. Actin was labeled with phalloidin‐AF546 (Molecular Probes, Eugene, OR, USA), incubated for 30 min at 37 °C in a humidified chamber, and nuclei were stained with 50 μg·mL^−1^ Hoechst 33342 (Sigma‐Aldrich, Darmstadt, Germany). For Tetramethylrhodamine Methyl Ester Perchlorate (TMRM) live staining, PDOs were incubated with 50 nm TMRM for 30′ at 37 °C (Thermo Fisher Scientific) and imaged in an environmental chamber at 37 °C and 5% CO_2_. Images were taken with a Leica TCS SP8 X confocal microscope. For time‐lapse videomicroscopy, cells or PDOs in Ibidi chamber slides were treated with indicated compounds and housed in a stage‐top live cell imaging chamber (Okolab, Rovereto, Italy) on a Leica AFLX6000 station, maintained in a humidified atmosphere at 37 °C and 5% CO_2_, and imaged every 20 min. Image analysis was performed with las x software (quantification mode with manual definition of ROI) by computing the GFP fluorescent of at least 24 individual cells and 9 PDOs per each biological replicate.

Cell lysates immunoblotting was performed as previously described [32]. Briefly, for the generation of lysates starting from organoids, CRC PDOs were collected and washed in cold PBS to allow removal of the extracellular matrix, and the pellet was resuspended with 2× Laemmli sample buffer (4% SDS, 20% glycerol, 0.004% bromophenol blue, 0.125 m Tris‐Cl pH 6.8, 10% 2‐mercaptoethanol).

The following primary antibodies were used: HDAC1 (Upstate, Lake placid, NY, USA, 06‐720), HDAC2 (Millipore, Darmstadt, Germany, 3F3), HDAC3 (CST, Danvers, MA, USA, 60538), HDAC8 (CST, 66042), HDAC4 [12], HDAC5 [12], HDAC7 [15], HDAC9 [12], GFP [31], FLAG M‐2 (Sigma‐Aldrich), γH2AX (CST, 9718), H3 (Sigma‐Aldrich), Actin (CST, D2O1K), anti‐pSPP (CST, 14390), GAPDH (Sigma‐Aldrich, 71.1), RAD51 (GeneTex, Irvine, CA, USA, GTX70230), RAD52 (Santa Cruz Biotechnology, Dallas, TX, USA, H‐300), Ubiquitin (Covance, Princeton, NJ, USA, P4D1), GSK3β (Santa Cruz Biotechnology, E‐11), FBXW7 (Thermo Fisher Scientific, A301721A; Bethyl Laboratories, Montogmery, AL, USA, OTI4C11; Merck, Darmstadt, Germany, MABS1118; Abcam, Cambridge, UK, ab109617), c‐Myc (CST, D84C12), TP53 [12], and Caspase 3 (CST, 9662). Original images used for the composition of the immunoblot panels are provided as Figs S8 and S9.

### 2.6 Chromatin immunoprecipitation and sequencing

Chromatin was obtained from HCT‐116 WT or *FBXW7* ^−/−^ cells. Chromatin immunoprecipitation was performed as previously described [12] with 3 μg of anti‐H3K27ac (Abcam, Cambridge, UK, ab4729) or 4 μg of anti‐H3K4me1 (Diagenode, Seraing, Belgium, C15410194). For each sample, 5 ng of total DNA was used to prepare ChIP‐seq libraries, according to the TruSeq ChIP Sample Preparation guide (Illumina, San Diego, CA, USA). Two biological replicates of each sample were subjected to library preparation and sequencing. PE150 with at least 30 m reads per samples were obtained after NGS for each library at Genewiz (Leipzig, Germany).

The ChIP‐seq data analysis was performed as previously described [12, 34]. Libraries were sequenced on the Illumina HiSeq 2000 sequencer. A minimum overlap of 30% of peaks between independent replicates was obtained and verified before the pulling. Read quality was assessed using fastqc. Raw reads were then trimmed to remove sequencing adapters and low‐quality reads using trimmomatic in paired‐end mode [35]. Reads were then aligned to the reference human genome (hg38) using bowtie2 [36]. Duplicate reads were removed using samtools rmdup [37]. Peak calling was performed using macs2 (‘sharp’ mode). The number of reads in each peak was counted and normalized using DiffBind to account for differences in sequencing depth and distribution [38]. Differential binding sites between groups were identified using edger with the following design formula ‘~ Replicate + Condition’ to account for batch effect between replicas. Finally, differential peaks (|FC| > 1.5, *P* < 0.05) were extracted by comparing the different groups [39]. gplots, biomaRt, and Gviz R/Bioconductor packages and the deepTools suite were used to generate peak heatmaps and for the visualization of genomic loci. The CHIPIN normalization algorithm was adopted for inter‐sample normalization of H3K27ac levels [40]. The ROSE algorithm was used to create stitched enhancers, separating super‐enhancers from typical enhancers. Only SE defined by copresence of H3K27ac and H3K4me1 were considered. Finally, the bedtools toolset was used to identify overlaps of at least one nucleotide between ROSE‐defined super‐enhancers and HCT‐116 defined topologically associated domains. HDAC4‐associated SEs were defined by intersecting identified SEs with HDAC4 peaks [31]. For motif enrichment analysis, CentriMo (local motif enrichment analysis) was used by interrogating the .bed files of the identified regulative regions, as previously described [12].

### 2.7 DR‐GFP and EJ5‐GFP reporter system

5 × 10^6^ TRI‐DR‐GFP and EJ5‐GFP U2OS cells [31] were seeded in 6‐well plates and transfected or not with 1 μg of I‐SceI endonuclease and 3 μL of Lipofectamine 2000 per well. After 16 h, cells were washed and treated with 20 μm Scr7, 20 μm Mirin, or 20 μm RI‐1 for 24 h. HR and NHEJ efficiency were determined by quantifying GFP‐positive cells by flow cytometry, as previously reported [31].

### 2.8 Flow cytometry and PI staining

For flow cytometry analysis, dead and alive cells were collected by trypsinization, resuspended in PBS, and filtered through 70 μm cell strainers. For propidium iodide (PI) incorporation, 10 μg·mL^−1^ PI (Sigma‐Aldrich) was added to the single‐cell suspension after filtration. GFP‐ or PI‐positive cells were acquired on a BD FACSCalibur cytometer and data were analyzed with flowjo software (BD, San Jose, CA, USA).

### 2.9 Co‐immunoprecipitation

Cells were lysed in a hypotonic buffer (20 mm Tris/HCl, pH 7.5; 10 mm MgCl_2_; 10 mm KCl; and 1% Triton X‐100) supplemented with protease inhibitors. For each immunoprecipitation, 1 μg of antibody was used. For the IP of polyubiquitylated proteins, the lysis buffer was supplemented with 1 mm NaVo_4_, 1 mm NaF, 100 mm iodoacetamide, and 5 μm MG132. Immunocomplexes were collected with 8 μL Dynabeads protein A/G (Thermo Fisher Scientific) and resolved by SDS/PAGE.

### 2.10 *In vitro* phosphorylation and ubiquitylation assay

FLAG‐tagged HDAC4 and its mutants were isolated from 293 cells. Cells were lysed with hypotonic low‐salt buffer (200 mm Tris/HCl pH 7.5, 1% Triton X‐100, 10 mm MgCl_2_, 10 mm KCl, 1% glycerol) for 15 min on ice. About 150 mm NaCl was added to stop the lysis. FLAG‐HDAC4 was recovered with α‐FLAG M‐2 antibody (Sigma‐Aldrich) and Dynabeads protein G (Thermo Fisher Scientific) for 5 h in a rotator mixer at 4 °C. For the *in vitro* phosphorylation, FLAG‐HDAC4 and its mutants were incubated with 150 ng of recombinant GSK3β (Cell Signalling Technology, PV3365) in 50 μL of kinase buffer (25 mm Tris/HCl pH 7.5, 10 mm MgCl_2_) completed freshly with 1 mm DTT, 1 mm NaVO_4_, 5 mm NaF, 200 μm ATP, and PIC, 30 min at 30 °C while shacking in a thermomixer. *In vitro* phosphorylated HDAC4 was washed once with ubiquitin reaction buffer (50 mm Tris/HCl pH 7.5, 5 mm MgCl_2_). Samples were incubated with ubiquitin reaction buffer added with 2 mm NaF, 200 μm ATP, 1 mm DTT, 30 μm MG132, PIC, 4 μg·mL^−1^ ubiquitin (Sigma‐Aldrich), 38 ng·μL^−1^ UBE1 E1 complex (EMD Millipore Corporation) (96 ng per reaction), 106 ng·μL^−1^ UbcH5a E2 complex (EMD Millipore Corporation) (2.7 μg per reaction), and 34 ng·μL^−1^ SCF^Fbxw7^ E3 ligase complex (EMD Millipore Corporation) (85 ng per reaction), as indicated in 25 μL volume. The reactions were incubated 30 min at 37 °C while shaking in a thermomixer. Samples were washed once with ubiquitin reaction buffer, then 2× Laemmli buffer was added, samples were heated 5 min at 70 °C and subjected to SDS/PAGE and immunoblotting.

### 2.11 Colony formation assay

2 × 10^4^ HCT‐116 cells per well were seeded in a 96‐well plate and treated with OXPT and/or PROTAC #11 at the indicated concentrations. After 24 h of treatment, cells were diluted 1 : 9 and seeded in a 60 mm plate; cells were left to grow for 8 days and medium was added every 2–3 days. Colonies were stained with methylene blue (1% in 50% ethanol) and rinsed with water. The imagej Colony Counter plugin was used for colonies quantification.

### 2.12 Caspase assay

The caspase activity was evaluated with the Apo‐ONE Homogeneous Caspase‐3/7 Assay (Promega, Madison, WI, USA). Cells were seeded in a 96‐well plate and silenced or not for FBXW7 and/or treated with OXPT and/or PROTAC #11 or STS (this latter for the last 16 h), as indicated. The assay was performed following the manufacturer instructions, and the fluorescence intensity of the substrate cleaved by caspase 3/7 was recorded every 5 min for 1 h. Caspase 3/7 activity reported in the graph corresponds to the point of *V*_max_ of fluorescence increase.

### 2.13 CRC PDOs ATP quantification

To assess CRC organoids' viability, ATP levels were measured with the CellTiter‐Glo 2.0 assay (Promega, USA). Organoids were seeded in a 96‐well plate and treated after 72 h with OXPT and/or PROTAC #11 at the indicated concentrations. 72 h later, ATP levels were assessed following the manufacturer instructions, and luminescence was recorded at 0–5–20 min. The quantification reports the mean of the three reads of three replicates, after normalization.

### 2.14 HDAC assay

Cells or PDOs were lysed with lysis buffer (0.5% Triton X‐100, 300 mm NaCl in PBS) supplemented freshly with PIC, 1 mm PMSF, and 1 mm NaVo_4_. Cells were lysed on ice for 10 min, and samples were centrifuged 10 min at 10 400 ***g*** at 4 °C. The protein content of the supernatant was quantified with Bradford assay (Euroclone). About 20 μg of lysates were used for each condition and diluted in a final volume of 100 μL of PBS supplemented freshly with PIC, 1 mm PMSF, and 1 mm NaVo_4_. Lysates were mixed 1 : 1 with ADS buffer (20 mm HEPES, 5.5 mm glucose, 0.8 mm NaH_2_PO_4_, 5.4 mm KCl, 0.8 mm MgSO_4_, 116 mm NaCl). As a control, scalar amounts of recombinant HDAC4 were used. About 200 μL of samples were aliquoted in a 96‐well black bottom plate, and 1 μL of 100 μm TMP195 was added in the selected wells to block class IIa HDAC activity, while 1 μL of 300 μm apicidin was added in the wells tested for class IIb HDACs activity to block class I activity. After incubation for 30 min at 37 °C, 5 μL of 1 mm Boc‐Lys‐(Tfa)‐AMC or Boc‐Lys‐(Ac)‐AMC substrates (Bachem AG, Bubendorf, Switzerland) were added to evaluate the activity of class IIa or class I and class IIb HDACs, respectively. The plate was incubated 1 h at 37 °C. About 50 μL of developer/stop solution (1.5% Triton X‐100, 3 μm TSA, 0.75 mg·mL^−1^ trypsin in PBS) were added in each well, and the plate was incubated for 20 min at 37 °C. The fluorescent signal was recorded every 10 min for 1 h with a BioTek plate reader (excitation/emission filters of 360/460 nm).

### 2.15 Xenograft mouse model

Athymic Nude‐*Foxn1nu* female mice (Envigo, Indianapolis, IN, USA), 6 weeks old, were housed in appropriate cages (IVC system, Sealsafe Plus GM500 cage Green line; Tecniplast, Buguggiate, Italy) and were kept in a controlled environment with controlled temperature (20–24 °C) and 50–60% humidity, with a light/dark cycle of 12 h. All animals were subjected to the same environmental conditions. Animals were delivered to the animal facility 14 days prior to tumor cell inoculation to ensure acclimatization of the animals for the study. Mice were injected subcutaneously in the right flank with 10^6^ HCT‐116 *FBXW7* ^−/−^ cells. Tumors were allowed to grow until they reached ~ 100 mm^3^ and mice were then randomized to the following treatment groups: vehicle (65% PBS, 35% H_2_O, 1.25% DMSO), oxaliplatin 5 mg·kg^−1^ + vehicle, oxaliplatin 5 mg·kg^−1^ + PROTAC #11 5 and 10 mg·kg^−1^, or PROTAC #11 alone. Each study group contained the same ratio of smaller and larger tumors. Cages were clearly labeled with an ID card indicating study number, group, gender, and treatment schedule. Treatments were administered intraperitoneally in a volume of 200 μL twice per week. Tumor volumes (TV) were estimated by measuring the width (*W*) and length (*L*) of the tumor using a digital caliper and calculated based on the following formula: TV = *W* ^2^ *L*/2. Mouse body weights and tumor volumes were measured twice per week over the course of each study. At the end of the experimental protocol, mice were euthanized, the tumors explanted and processed to evaluate the effectiveness of the treatment with molecular biology experiments. Furthermore, all internal organs were evaluated and inspected for any toxicities that were not obvious. Mice were euthanized by cervical dislocation. *In vivo* experiments were carried out at MTTLab (Trieste, Italy) and Biogem (Ariano Irpino, AV, Italy) (Ministerial Authorization no. 257/2023‐PR). The experiments were performed in accordance with national legislation (Ministerial Authorization n. 257/2023‐PR and n. 695/2025‐PR). Raw data are available in Table S8. Ethical documentation is provided as File S1.

### 2.16 Tissue microarray (TMA) and immunohistochemical analyses

Paraffin‐embedded samples from colon adenocarcinoma were available from 15 patients (grade II/III CRC). About 2 mm core sections of tumor and matched normal adjacent tissue are available for each patient (Hcol‐ade030PG‐01, Table 3.1, US Biomax, Derwood, MD, USA). Antigen retrieval was performed with citrate buffer at pH 6. IHC was performed to detect HDAC4 (with the antibody characterized previously [41], 1 : 100). As a negative control, sections were stained without adding the primary antibody. TMA‐stained slides (HE, HDAC4) were scanned at 40x magnification (Aperio AT2, Leica, Nubloch, Germany). Scanned images were processed with qupath software. qupath's segmentation feature was used to detect single cells, and a training session assisted by a pathologist was used to assist the identification by the software of tumor cells. Quantification of HDAC4 positivity was obtained by applying the script previously used by the Tavora group [42] with the Positive Cell Detection command within the software. Mean intensities and percentages of duplicate cores were used for the final analysis.

### 2.17 Statistical analysis

For experimental data, Student's *t*‐test was employed. Mann–Whitney test was applied when normality could not be assumed. *P* < 0.05 was chosen as the statistical limit of significance. For comparisons between more than two samples, the ANOVA test was applied coupled to Kruskal–Wallis and Dunn's multiple comparison test. Where indicated, Bonferroni correction for multiple testing was applied. For correlation between two variables, Spearman correlation was calculated. Excel and graphpad prism were used for routine analysis, R/Bioconductor packages for large data analysis and heatmap generation. We marked with *P < 0.05, **P < 0.01, ***P < 0.001. Unless otherwise indicated, all the data in the figures were represented as arithmetic means ± the standard deviations from at least three independent experiments.

## 3 Results

### 3.1 Inhibition of homologous‐recombination repair (HRR) triggers HDAC4 degradation

**Fig. 1:**
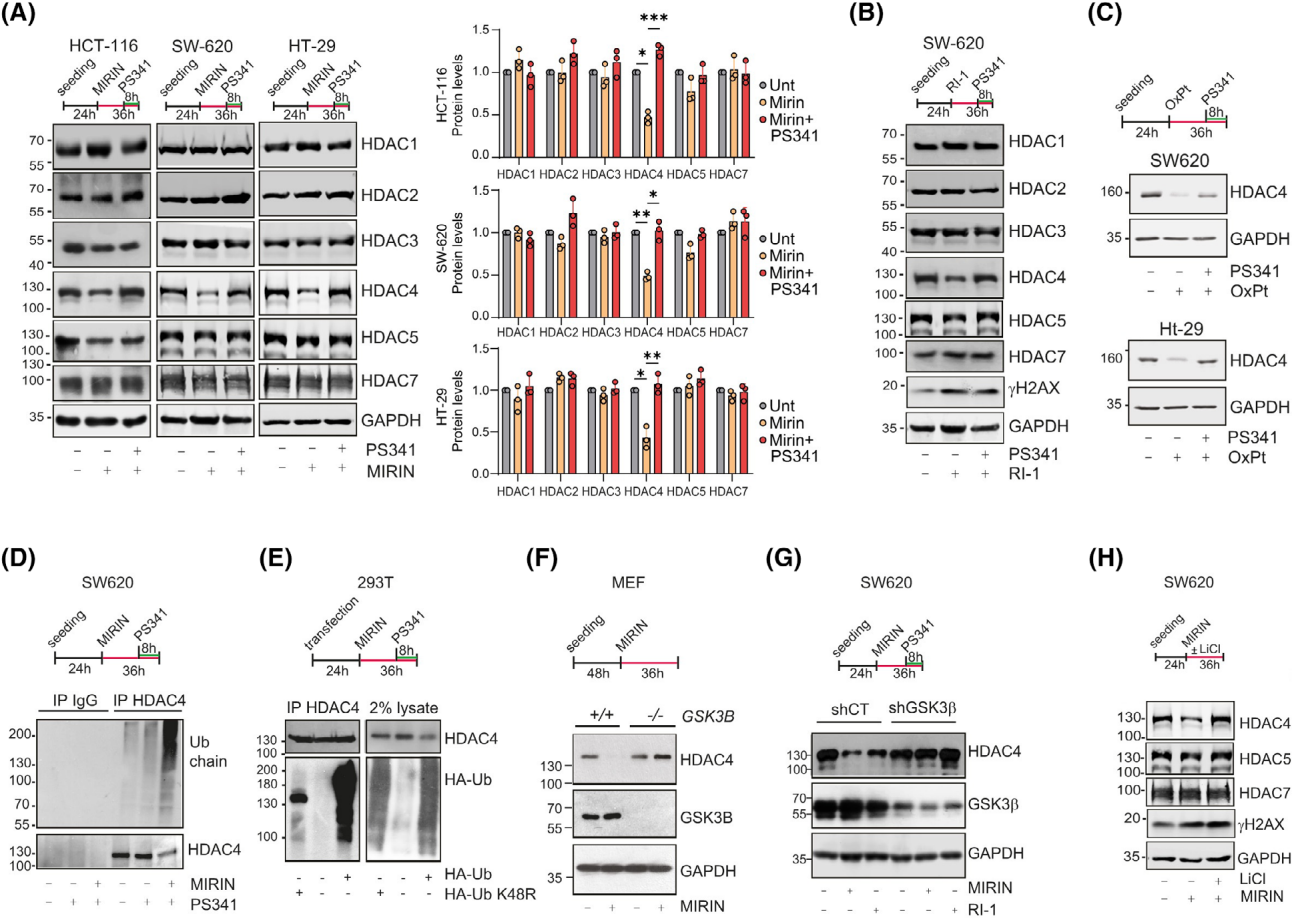
Inhibition of homologous‐recombination repair triggers HDAC4 degradation. (A) Immunoblot analysis of the indicated proteins in various CRC cell lines treated as specified (Mirin 20 μm, PS‐341 1 μm). Quantification is shown in the accompanying histogram. Data are presented as mean ± standard deviation, *n* = 3. Statistical significance was assessed using Tukey's multiple comparison test. (B) Immunoblot analysis in SW620 cells treated as indicated (RI‐1 20 μm, PS‐341 1 μm). *n* = 3. (C) Immunoblot analysis of the indicated proteins in SW620 or HT‐29 cells treated as indicated (OXPT 20 μm, PS‐341 1 μm). *n* = 3. (D) Polyubiquitylation assay on HDAC4 immunopurified from SW620 cells treated or not as indicated with Mirin 20 μm and PS‐341 1 μm. *n* = 3. (E) Polyubiquitylation assay on HDAC4 immunopurified from 293T cells transfected with 5 μg FLAG‐HDAC4 and 5 μg HA‐Ub WT or K48R HA‐Ub mutant and treated with Mirin 20 μm and PS‐341 1 μm, as indicated. *n* = 3. (F) Immunoblot analysis of the indicated proteins in MEF cells WT or *GSK3β* ^−/−^ and treated or not with Mirin 20 μm, as indicated. *n* = 3. (G) Immunoblot analysis of the indicated proteins in SW620 cells stably silenced for GSK3β, as indicated, and treated as indicated (Mirin 20 μm, RI‐1 20 μm). *n* = 3. (H) Immunoblot analysis of the indicated proteins in SW620 cells treated as specified (Mirin 20 μm, LiCl 10 mm). Recent findings [12] indicate that this concentration of LiCl is adequate to effectively inhibit GSK3β enzymatic activity. *n* = 3.

Defects in HRR have been correlated to increased sensitivity to OXPT in different cancer types, including CRC [43, 44]. Importantly, HDAC inhibitors have been reported to impair HRR [45, 46], while scattered information is available about the mutual influences of HRR players and HDACs [45, 47]. HDAC4 has been reported to be proteasomal degraded under impaired HRR cellular conditions, such as cellular quiescence [48] and senescence [12]. We therefore decided to investigate the effects of HRR inhibition on the expression of class I and IIa HDACs. We confirmed that the protein levels of HDAC4 (Fig. S1A), but not that of HDAC7 or the tested class I HDACs, were decreased in SK‐LMS‐1 cells treated with the HRR inhibitor Mirin [49]. HDAC5 was slightly downregulated by Mirin (Fig. S1A), while HDAC9 was not expressed in these cells [12]. These data demonstrate that among the HDACs tested, HDAC4 is the one most downregulated by HRR blockade. Proteasomal inhibition with Bortezomib, hereafter referred to as PS‐341, restored HDAC4 protein levels (Fig. S1A), suggesting that the ubiquitin–proteosome system (UPS) is responsible for HDAC4 instability under these HRR‐blocking conditions. HDAC4 protein levels (Fig. S1B), but not mRNA (Fig. S1C), were also decreased in cells silenced for RAD51 and RAD52—two key executor of HR repair [35]—with an effect similar to Mirin both in terms of HDAC4 destabilization and accumulation of the phosphorylated and ubiquitylated forms of H2AX (Fig. S1B). To further support this concept, we treated three HR‐proficient colorectal cancer cell lines [50]—HT‐29, SW620, and HCT‐116—with Mirin and the RAD51 inhibitor RI‐1 [51]. Both compounds were applied at a concentration of 20 μm, which selectively inhibits the HR pathway without interfering with nonhomologous end joining (NHEJ), as shown in Fig. S1C. The NHEJ inhibitor Scr7 [52] was used for comparison (Fig. S1C). As reported in Fig. 1A, Mirin induced a reduction in HDAC4 protein levels across all three CRC cell lines. In all cases, cotreatment with PS‐341 restored HDAC4 protein levels, indicating that Mirin induces HDAC4 degradation via a proteasome‐dependent mechanism (Fig. 1A). The expression of other HDACs remained largely unaffected, except for HDAC5, which showed a modest, nonsignificant reduction following Mirin treatment, and a partial recovery upon PS‐341 cotreatment in both SW620 and HCT‐116 cells (Fig. 1A). HDAC4 is also degraded following treatment with RI‐1 in SW620 cells (Fig. 1B), indicating that this effect is not exclusive to Mirin.

OXPT was reported to inhibit the expression of key HRR players [53], therefore, its effect on HDAC4 stability was tested in CRC cells. A panel of HR‐proficient CRC cell lines was treated with increasing concentrations of OXPT, and the growth inhibition [54] (GI₅₀) values were determined (Fig. S1E). Subsequently, SW620 and HT‐29 cells were treated with 20 μm OXPT, which produced comparable effects in both cell lines in terms of cellular fitness and accumulation of DNA double‐strand breaks (DSBs) (Fig. S1F). OXPT treatment decreased HDAC4 protein levels in both CRC cell lines; proteasomal inhibition restored almost completely HDAC4 levels (Fig. 1C). As further confirmation, HR repair was disrupted by silencing BRCA1 expression using two distinct shRNAs (Fig. S1G), resulting in a reduction of HDAC4 protein levels in BRCA1‐deficient cells—both under basal conditions and, more markedly, following treatment with OXPT. Finally, HDAC4 protein levels were also reduced following treatment with 100 nm of the ATR inhibitor Berzosertib (hereafter referred to as M6620, [55]) with near‐complete restoration upon cotreatment with PS‐341 (Fig. S1H).

To confirm that HDAC4 is degraded via the UPS after HRR blockade, HDAC4 was immunoprecipitated from SW620 cells treated or not with Mirin in the presence or absence of proteasome inhibitor PS‐341. Accumulation of HDAC4 polyubiquitylated species was evident after Mirin and PS‐341 cotreatment (Fig. 1D), confirming the activation of a UPS degradation process. Assisted degradation of HDAC4 after HRR inhibition required its polyubiquitylation with K48‐linked polyubiquitin chains, since transfection of K48R ubiquitin abolished HDAC4 polyubiquitylation in Mirin‐treated 293 cells (Fig. 1E).

In quiescent and senescent cells, the phosphorylation of HDAC4 by GSK3β was reported to prime its UPS degradation [12, 48]. GSK3β was required to stimulate HDAC4 degradation even in HRR‐blocked cells, since GSK3β^−/−^ murine fibroblasts (Fig. 1F) and GSK3β‐depleted SW620 cells (Fig. 1G) treated with Mirin failed to degrade HDAC4, unlike WT or control cells. Moreover, treatment with LiCl, a GSK3β inhibitor [56], restored HDAC4 protein levels in the presence of Mirin (Fig. 1H).

Altogether, GSK3β was found to be required for the UPS‐driven degradation of HDAC4 in conditions of HRR blockage.

### 3.2 Identification of FBXW7 as the E3‐ligase responsible for HDAC4 degradation in HRR‐impaired cells

An *in silico* screening was performed to identify the E3‐ligase responsible for HDAC4 degradation. The anticorrelation between the protein levels of HDAC4 and 365 E3 ligases was assessed by computing the mass spectrometry data available for 375 cancer cell lines of the Cancer Cell Line Encyclopedia [57] (Table S1). Fourteen E3‐ligases were found to be negatively correlated with HDAC4 and HDAC5 (the class IIa HDACs most abundant across these samples) (Fig. S2A). After secondary screening (Fig. S2B–D), we selected four E3‐ligases (FBXW7, MARCH5, MARCH6, RNF185) whose silencing induced the accumulation of HDAC4 after treatment with Mirin (Fig. S2E). A tertiary screening was conducted on tumor cells silenced for these four E3 ligases and treated with Mirin (Fig. S2F) or depleted of BRCA1 (Fig. S2G). In both these conditions, the knockdown of the SCF E3‐ligase FBXW7 was found to stabilize HDAC4 (Fig. S1F,G). Knockdown of RNF185 had only a minimal effect, while the depletion of the mitochondrial E3‐ligases MARCH5 and MARCH6 under these conditions led to the accumulation of high‐molecular weight forms of HDAC4, which will be investigated in future work (Fig. S2F,G). Overall, this screening identified FBXW7 among the E3‐ligases most likely responsible for HDAC4 polyubiquitylation under HRR‐blocking conditions.

### 3.3 FBXW7 controls the ubiquitylation of HDAC4 in OXPT‐treated cells

**Fig. 2:**
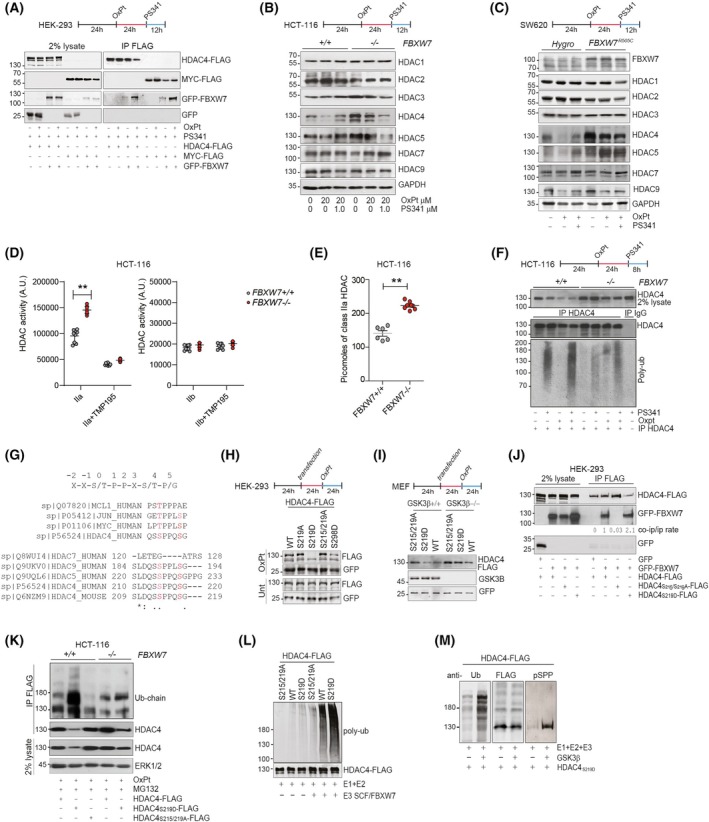
FBXW7 controls the ubiquitylation of HDAC4 in OXPT‐treated cells. (A) About 293 were transfected with 5 μg pFLAG‐HDAC4 or MYC‐FLAG and pEGFPC1 or pEGFPC1‐FBXW7. After 24 h, cells were treated or not with OXPT 20 μm and for the last 12 h with PS‐341 1 μm. About 1 μg anti‐FLAG was used for immunoprecipitation, anti‐GFP to detect GFP and GFP‐FBXW7. 2% lysate was used as input. *n* = 3. (B) Immunoblot analysis of the indicated proteins in HCT‐116 WT or *FBXW7* ^−/−^ cells treated or not as indicated with OXPT 20 μm and proteasome inhibitor PS‐341 1 μm. *n* = 3. (C) Immunoblot analysis of the indicated proteins in the indicated SW620 cells treated or not as indicated with OXPT 20 μm and PS‐341 1 μm. *n* = 3. (D, E) Class IIa and class IIb HDAC assay in lysates obtained from WT or *FBXW7* ^−/−^ HCT‐116 cells. TMP195 was used *in vitro* to block class IIa HDAC activity as a control. Data are represented as means ± standard deviation, *n* = 8 (in D), 6 (in E). In (E) HDAC assay was used to estimate class IIa HDAC quantity by using scaling doses of recombinant HDAC4 as standard. (F) Polyubiquitylation assay on endogenous HDAC4 immunopurified from WT or *FBXW7* ^−/−^ HCT‐116 cells, treated or not as indicated with OXPT 20 μm and PS‐341 1 μm. 2% lysate was used as input. *n* = 3. (G) Alignments of putative FBXW7 phosphodegrons in class IIa HDACs in respect to known FBXW7 substrates. (H, I) Immunoblot analysis in 293 (H) and MEF (I) transfected with the indicated pFLAG‐HDAC4 mutants (2 μg) and pEGFPC1 (200 ng), as a reference. *n* = 3. (J) About 293 were transfected with 5 μg of the indicated pFLAG‐HDAC4 mutants and pEGFPC1 or pEGFPC1‐FBXW7. After 24 h, cells were treated or not with OXPT 20 μm and for the last 12 h with PS‐341 1 μm. About 1 μg anti‐FLAG was used for immunoprecipitation, anti‐GFP to detect GFP and GFP‐FBXW7. 2% lysate was used as input. *n* = 3. (K) Poly‐ubiquitylation assay on HDAC4‐FLAG immunopurified from WT or *FBXW7* ^−/−^ HCT‐116 cells lipofected with 2.5 μg of the indicated pFLAG‐HDAC4 plasmids. After 36 h, cells were treated for 36 h with OXPT 20 μm and for the last 12 h with PS‐341 1 μm, as indicated. Anti‐Ub was used to detect endogenous ubiquitylation. 2% lysate was used as input. *n* = 3. (L) *In vitro* polyubiquitylation assay on indicated HDAC4‐FLAG mutants immunopurified from transfected 293 cells incubated with E1, E2, and E3 complex containing FBXW7, as indicated. Anti‐FLAG was used to detect HDAC4‐FLAG, anti‐Ub was used to detect polyubiquitylation. *n* = 3. (M) *In vitro* polyubiquitylation assay on indicated HDAC4 S219D‐FLAG immunopurified from transfected 293 cells incubated firstly with GSK3β and then with E1, E2, and E3 complex containing FBXW7, as indicated. Anti‐FLAG was used to detect HDAC4‐FLAG, anti‐Ub was used to detect polyubiquitylation, anti‐pSPP (specific for Ser‐Pro‐Pro) was used to detect GSK3β phosphorylation of Ser 215. *n* = 3.

FBXW7 is an SCF (SKP1‐CUL1‐F‐box) E3‐ligase described to control the GSK3β‐dependent degradation of well‐known proto‐oncogenes [58]. Here, we investigated the role played by FBXW7 in controlling the polyubiquitylation of HDAC4 in OXPT‐treated cells. First, 293 cells were cotransfected with HDAC4‐FLAG or MYC‐FLAG and GFP or GFP‐FBXW7 and treated for 36 h with OXPT in the presence of the proteasome inhibitor PS‐341 for the last 12 h (Fig. 2A). GFP‐FBXW7 was successfully copurified both with HDAC4‐FLAG and MYC‐FLAG. In both cases, the interaction was stronger after OXPT treatment (Fig. 2A). Then, we generated *FBXW7* ^−/−^ HCT‐116 cells which were validated by Sanger sequencing (Fig. S3A). We used a panel of commercially available antibodies directed against FBXW7 to validate the knockout (KO) (Fig. S3B). Although the considerable number of nonspecific signals severely limited their use in applications such as immunopurification or immunostaining, two of them proved to be of sufficient quality to be used in immunoblots identifying a band in the WT lysate that is not visible in the KO (Fig. S3B). Then, *FBXW7* ^+/+^ and *FBXW7* ^−/−^ HCT‐116 cells were treated for 36 h with OXPT in the presence or absence of PS‐341 for the last 12 h and subjected to immunoblot evaluation of the protein levels of class I and IIa HDACs. The protein levels of HDAC1, 2, 3, 7, and 9 were minimally affected by OXPT treatment and minimally different between WT and KO cells (Fig. 2B). HDAC4 protein levels were increased in KO cells in respect to WT, and OXPT did not significantly affect its expression in KO cells, while it more than halved HDAC4 levels in WT cells (Fig. 2B and Fig. S3C). PS‐341 treatment completely rescued HDAC4 levels in WT cells (Fig. 2B and Fig. S3C). Interestingly, in KO cells, cotreatment with OXPT and PS‐341 or MG132 leads to a reduction in both HDAC4 transcript (Fig. S3C) and protein levels (Fig. 2B and Fig. S3C,K). The pattern of HDAC5 protein levels was very similar to that of HDAC4, although the regulation by OXPT was attenuated, probably for compensatory increase in HDAC5 mRNA (Fig. 2B and Fig. S3C,D).

Three FBXW7 hotspot missense mutations (R465, R479, and R505) were identified in CRC, all of them falling within the substrate recognizing WD40 domain [59] (Fig. S3E,F). These LOF mutations prevent the ubiquitylation and degradation of certain FBXW7 targets, thus exerting a selective dominant‐negative effect when present in heterozygosity with the WT allele [58, 59]. We generated a plasmid encoding GFP‐FBXW7^R505C^, and we evaluated its capability to act as a dominant‐negative allele of FBXW7 toward MYC [58]. We confirmed that FBXW7^R505C^ acted as a dominant negative toward FLAG‐MYC (Fig. S3G). Then, we expressed FBXW7^R505C^ in SW620 CRC cells (Fig. 2C). As a control, the same cells were infected with the same empty lentiviral plasmid (indicated as Hygro in the figure). Similarly to what observed in HCT‐116 *FBXW7* ^−/−^ cells, the expression of FBXW7^R505C^ in SW620 led to increased protein levels of HDAC4 and HDAC5 and made them stable even in the presence of OXPT (Fig. 2C and Fig. S3H). The other HDACs tested were not significantly altered by FBXW7^R505C^ expression or OXPT treatment, except for HDAC9 whose protein levels partially decreased after OXPT treatment (Fig. 2C). Importantly, FBXW7^R505C^ did not interact with HDAC4, differently from WT (Fig. S3I). As further confirmation, HDAC4 was not found significantly degraded in CRC cells carrying FBXW7 loss‐of‐function mutations (SW837: *FBXW7* ^L403,wt^*; LoVo: *FBXW7* ^*R505C,wt*^) after treatment with OXPT (Fig. S3J). As the downregulation of HDAC4 turned out to be prevalent, we further investigated its degradation.

Importantly, class IIa HDACs' activity, but not class IIb, was increased in *FBXW7* ^−/−^ HCT‐116 cells in respect to WT ones (Fig. 2D). For further confirmation, we performed the class IIa HDAC assay on lysates from WT and *FBXW7* ^−/−^ HCT‐116 cells compared to defined amounts of recombinant HDAC4 as a standard. We estimated that approximately 210 picomoles of class IIa HDAC were present in 30 μg of lysates in *FBXW7* ^−/−^ cells compared to approximately 140 picomoles in WT cells (Fig. 2E).

FBXW7 depletion was found to strongly impair the accumulation of polyubiquitylated HDAC4 in untreated and to an even greater extent in OXPT‐treated cells (Fig. 2F), while no alterations in TP53 levels were found when comparing WT and KO cells (Fig. S3K). F‐box proteins recognize and bind to phosphodegrons in their substrates [60]. Proline‐directed kinases phosphorylate FBXW7 substrates within the phosphodegron Ser/Thr‐Pro‐Pro‐X‐Ser/Thr [61] controlling their recognition and binding by WD40 domains [62]. However, some deviations from the canonical consensus are tolerated [61] (Fig. 2G) and dimerization of FBXW7 was shown to promote the turnover of weakly associated/weak substrates (e.g., Cyclin E and Sic1) *in vivo* [62, 63, 64]. A consensus site of FBXW7 is located in HDAC4 at amino acids 215–219 (Fig. 2G). These two phosphorylation sites are conserved between murine and human HDAC4 and between all members of class IIa HDACs, except HDAC7, the most divergent and the only one lacking a glutamine‐rich region at the N‐terminus [65]. Alanine substitutions of the phosphorylation sites were reported to abrogate FBXW7 recognition, while phosphomimicking (Ser/Thr‐ > Asp/Glu) mutants were tolerated at the +4 position [61]. We therefore constructed phospho‐dead (S219A, S215/S219A) and phosphomimicking (S219D) mutants of HDAC4‐FLAG and tested their stability in 293 cells treated or not with OXPT, compared to a mutant of HDAC4 (S298D) that we previously characterized to be unstable [12, 31]. As a control, we cotransfected the pEGFPC1 plasmid. HDAC4^S219D^ was found to be unstable in OXPT‐treated condition, while phospho‐dead mutants displayed increased stability (Fig. 2H). Interestingly, also the S298D mutant showed partial instability, as previously observed [48], probably controlling HDAC4 degradation in a FBXW7‐independent manner. We repeated the same experiment in *GSK3β* ^+/+^ and *GSK3β* ^−/−^ MEF and confirmed the increased instability of the phospho‐dead mutant (S215/S219A) in respect to the WT and the phosphomimicking (S219D) mutant of HDAC4 in *GSK3β* ^+/+^ MEF (Fig. 2I). These data support the notion that serine 219 serves as a +4 priming site [66] for GSK3β‐mediated phosphorylation of serine 215 and the subsequent recognition by FBXW7. In support of this, only the WT form of HDAC4 and to a greater extent HDAC4^S219D^ interact with FBXW7, but not HDAC4^S215/219A^ (Fig. 2J). Moreover, OXPT treatment induced a strong ubiquitylation of HDAC4^S219D^, but not of HDAC4^S215/S219A^, only in *FBXW7* ^+/+^ HCT‐116 cells, but not in *FBXW7* ^−/−^ ones (Fig. 2K). Finally, FBXW7 was found to trigger the *in vitro* polyubiquitylation of HDAC4 in a GSK3β‐dependent manner. A strong *in vitro* polyubiquitylation was observed on WT and, to a greater extent, on HDAC4^S219D^‐FLAG immunopurified from 293 cells incubated with E1 + E2 + E3 FBXW7‐SCF complex (Fig. 2L); this activity was busted by pre‐incubating HDAC4^S219D^ with recombinant GSK3β that triggers HDAC4 phosphorylation (Fig. 2M). Phosphorylation was detected with anti‐pSPP [67]. Since this antibody has serine 215 as its only possible substrate in HDAC4, we concluded that GSK3β catalyzes its phosphorylation.

Altogether, GSK3β controls FBXW7‐dependent degradation of HDAC4 in OXPT‐treated cells.

### 3.4 HDAC4 expression is increased in CRC

**Fig. 3:**
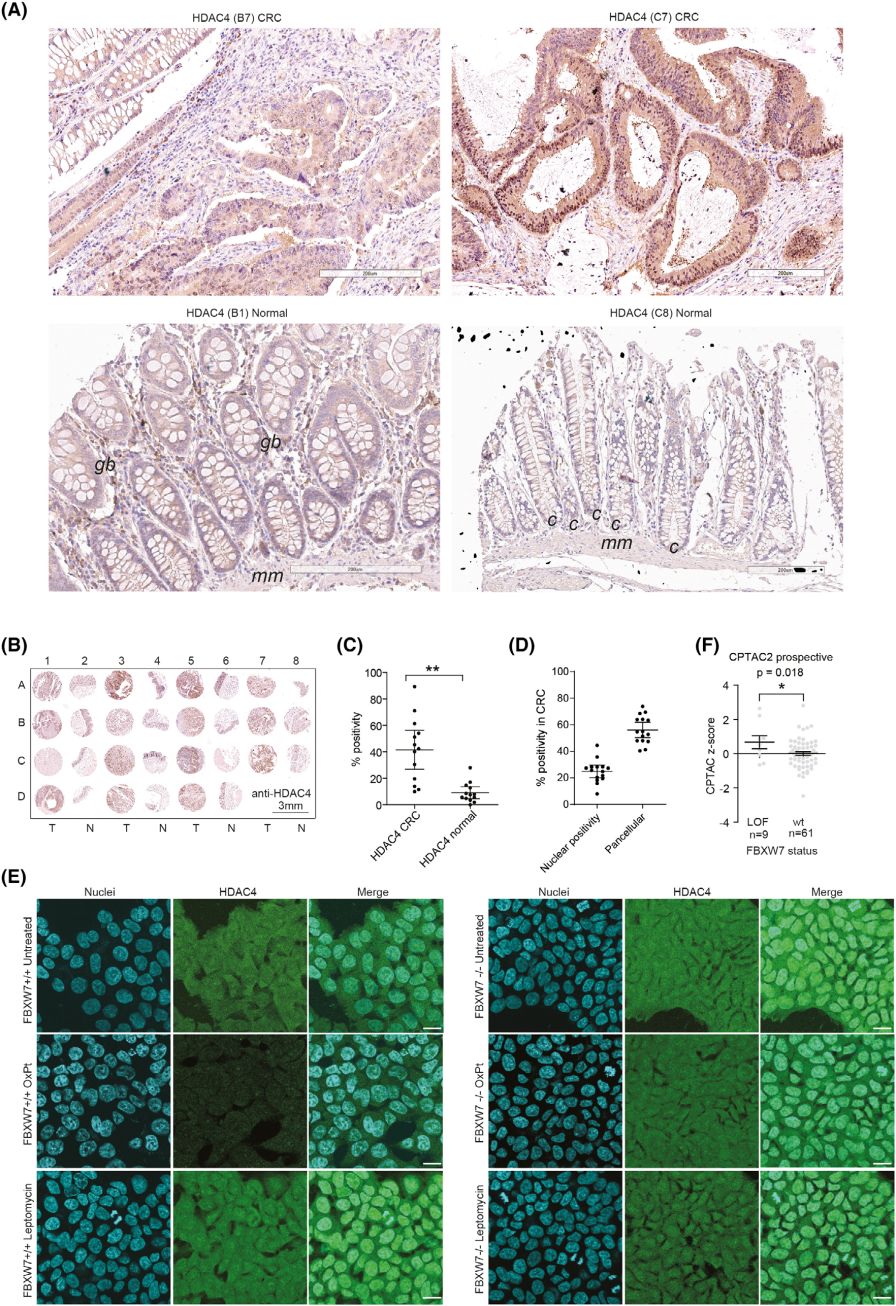
HDAC4 expression is increased in CRC. (A) Exemplary images of HDAC4 immunostaining after the development of the peroxidase reaction in the indicated core cases of CRC. Scale bar = 200 μm. gb = Goblet cells, mm = muscularis mucosae, c = stem cell crypt. (B) HDAC4 immunostaining after the development of the peroxidase reaction in the described TMA. For each patient, the neoplastic biopsy sample is indicated by an odd number, while the healthy sample is immediately adjacent and indicated by an even consecutive number. Scale bar = 3 mm. (C) Tukey box plot illustrating the percentage of cells positive for HDAC4 after their immunodetection in TMA slides of 15 grade II‐III CRC biopsies and the matched normal tissue. Wilcoxon test was used to quantify the significance of comparisons. Wilcoxon rank‐sum test. Mean and 1st and 3rd quartile are indicated. (D) Tukey box plot illustrating the percentage of cells positive for HDAC4 with nuclear staining or diffuse pancellular staining in CRC biopsies. Wilcoxon test was used to quantify the significance of comparisons. Wilcoxon rank‐sum test. Mean and 1st and 3rd quartile are indicated. (E) Representative confocal images of HDAC4 immunostaining in WT or *FBXW7* ^−/−^ HCT‐116 cells, treated or not with OXPT 20 μm for 30 h or for 2 h with Leptomycin B (50 μg·mL^−1^), to block HDAC4 nuclear export. Hoechst staining was used for nuclei. Scale bar = 15 μm. (F) Tukey box plot illustrating the absolute quantification of HDAC4 in CRC patients divided into two groups in relation to the mutational status of FBXW7, as indicated. Data are expressed as z‐score and were retrieved through cBioPortal from CPTAC2 consortium [70]. Wilcoxon rank‐sum test. Mean and 1st and 3rd quartile are indicated.

FBXW7 is reported to be downregulated or mutated in a relevant proportion of CRC patients [24, 59] (Fig. S3E,F) and FBXW7 LOF mutations or low FBXW7 mRNA expression predicted poor overall survival [59]. HDAC4 expression was found to be elevated in human small intestinal crypts [68] and in CRC [69] and was reported to support the proliferation [68] and dedifferentiation [69] of CRC cells. To confirm the relevance of HDAC4 in CRC, we performed IHC evaluation of HDAC4 protein levels in a tissue microarray consisting of 15 grade II/III colon adenocarcinoma biopsies and 15 normal adjacent tissues (Table S2, Fig. 3A,B). Increased positivity to HDAC4 was found in cancer tissues compared to normal ones (Fig. 3B,C). In normal tissue, HDAC4 was detected only in the stem cell niche of crypts (indicated as *c* in sample C8) and in Goblet cells (indicated as *gb* in sample B1) and in *muscularis mucosae* (*mm* in Fig. 3A), in confirmation of what previously reported [68]. In CRC, HDAC4 staining was mainly pancellular in all the samples, with few exceptions displaying nuclear localization (Fig. 3D). A similar pancellular localization of HDAC4 was observed in HCT‐116 CRC cells, and a decrease in both the nuclear and cytoplasmic signals of HDAC4 was evident after OXPT treatment in *FBXW7* ^+/+^ but not *FBXW7* ^−/−^ cells (Fig. 3E).

The lack of good commercial antibodies prevents the detection of FBXW7 in IHC. Therefore, to correlate HDAC4 protein levels to the mutational status of FBXW7, we interrogated the data obtained by the first proteogenomic study on CRC [70]. Despite the asymmetry of the patient cohort containing few samples with LOF of FBXW7 (LOF *n* = 9, wt *n* = 61), significant increased HDAC4 protein levels were found in samples characterized by FBXW7 LOF (Fig. 3F).

In summary, HDAC4 is expressed *in vivo* in colon adenocarcinoma specimens and its elevated levels compared to normal tissue designate it as a putative therapeutic target for CRC.

### 3.5 HDAC4 silencing or forced degradation with compound #11 restores OXPT sensitivity in FBXW7 depleted or mutated CRC cells and in a murine xenograft model of CRC

**Fig. 4:**
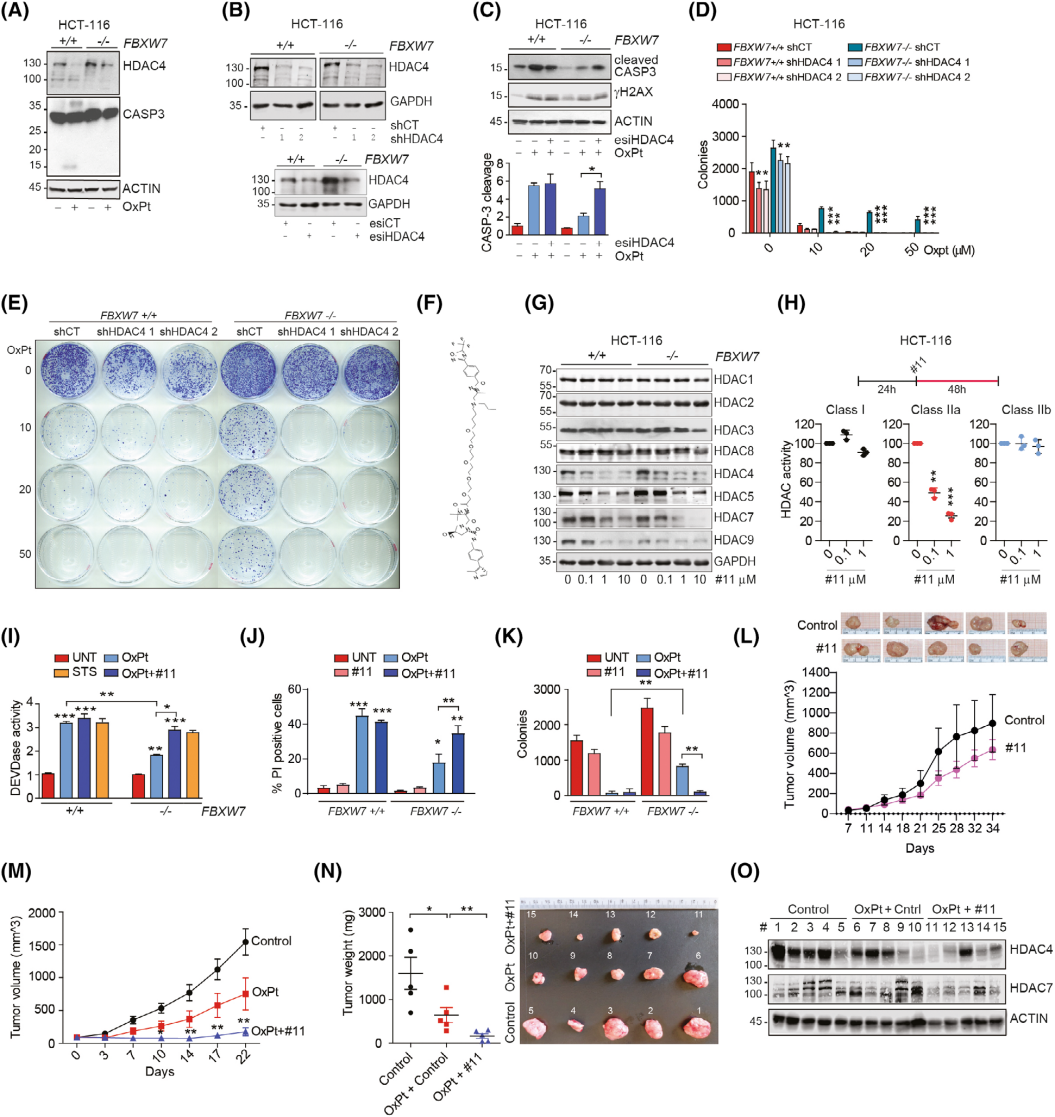
HDAC4 silencing or forced degradation with PROTAC compound #11 restores OXPT sensitivity in FBXW7 depleted or mutated CRC cells and in a murine xenograft model of CRC. (A) Immunoblot analysis of the indicated proteins in HCT‐116 WT or *FBXW7* ^−/−^ cells treated with 50 μm OXPT for 60 h. *n* = 3. (B) Immunoblot analysis of the indicated proteins in HCT‐116 WT or *FBXW7* ^−/−^ cells, transiently (48 h, with esiRNA) or stably silenced for HDAC4, as indicated. Two shRNAs were used as indicated. *n* = 3. (C) Immunoblot analysis of the indicated proteins and quantification of caspase cleavage in HCT‐116 WT or *FBXW7* ^−/−^ cells, transfected with HDAC4 esiRNAs (or control) and treated for 48 h with 50 μm OXPT. *n* = 3. (D, E). Quantification and representative images of the colony formation assay in HCT‐116 WT or *FBXW7* ^−/−^ cells, stably expressing the indicated shRNA for HDAC4 or shCT and treated or not for 24 h with 50 μm OXPT and kept growing for other 7 days. Significance in respect to untreated condition. *n* = 3. (F) Structural formula of HDAC4‐specific PROTAC degrader (# 11). (G) Immunoblot analysis of the indicated proteins in HCT‐116 WT or *FBXW7* ^−/−^ cells treated for 48 h with PROTAC degrader # 11, as indicated. *n* = 3. (H) Class I, IIa, IIb HDAC assay performed on lysates obtained from the indicated cells after 48 h treatment with the indicated amount of #11. Significance in respect to untreated condition. *n* = 3. (I) DEVDase activity evaluated by Apo‐ONE assay in HCT‐116 WT or *FBXW7* ^−/−^ cells, treated as indicated (OXPT 50 μm 40 h, #11 1 μm 40 h or Staurosporine (STS) 1 μm 16 h). Indicated pairwise comparisons or significance in respect to UNT conditions. *n* = 3. (J) Percentage of propidium iodide (PI) positive HCT‐116 WT or *FBXW7* ^−/−^ cells, treated for 72 h as indicated. Indicated pairwise comparisons or significance in respect to UNT conditions. *n* = 3. (K) Quantification of the colony formation assay in HCT‐116 WT or *FBXW7* ^−/−^ cells, treated as indicated for 24 h and kept growing for 7 days. Indicated pairwise comparisons. *n* = 3. (L, M, N) Quantification of tumor volumes (L, M) and tumor weights and picture (N) of HCT‐116 *FBXW7* ^−/−^ xenograft model. Nude mice were subcutaneously injected with 10^6^ HCT‐116 *FBXW7* ^−/−^ cells. When the tumor volumes reached a mean of 100 mm^3^, mice were randomized in groups and treated twice per week with vehicle, #11 5 mg·kg^−1^, OXPT 5 mg·kg^−1^ + vehicle or OXPT 5 mg·kg^−1^ + #11 5 mg·kg^−1^. After indicated days, mice were sacrificed and tumors excised and weighed *n* = 5. ANOVA and Bonferroni post‐test. (O) Immunoblot analysis of the indicated proteins in excised tumors depicted in (N). The numbering of the samples corresponds to the explanted tumors. In C, D, H, I, J, K data are represented as means ± SD, *n* = 3. *t*‐test was used for pairwise comparison.

FBXW7 LOF mutations decreased the efficacy of OXPT‐based chemotherapy [2, 3]. We confirmed the reduced activation of caspase‐3 and compromised degradation of HDAC4 in HCT‐116 *FBXW7* ^−/−^ cells after the treatment with OXPT (Fig. 4A). To demonstrate that forced removal of HDAC4 resensitized *FBXW7* ^−/−^ HCT‐116 cells to OXPT, we silenced its expression transiently with esiRNAs or stably with two different shRNAs (Fig. 4B). HDAC4 depletion restored caspase‐3 cleavage in OXPT‐treated cells (Fig. 4C) and resulted in resensitization to OXPT (Fig. S4A,B). Depletion of HDAC4 almost completely impaired the formation of OXPT‐resistant colonies in *FBXW7* ^−/−^ HCT‐116 cells (Fig. 4D,E). It should be also noted that HDAC4 depletion causes a decrease in proliferation and colony formation in both WT and FBXW7 KO cells (Fig. 4D,E), as previously reported [68].

The poor catalytic activity of HDAC IIa makes them pharmacologically attractive but at the same time difficult to target [71]. Trifluoromethyloxadiazole‐based compounds [72] proved efficacy in boosting anticancer immunity in CRC [73], similarly to what reported by original developers in breast cancer [74], but had limited efficacy on tumor cells [34], as well as putative nonspecific effects [75]. Targeted protein degradation achieved with proteolysis‐targeting chimera (PROTAC) protein degraders is an expanding field in antineoplastic pharmacology, especially for targets that are difficult to inhibit with conventional approaches [76]. Recently, selective degradation of HDAC4 was achieved with a PROTAC compound (herein defined as #11, Fig. 4F) based on a Trifluoromethyloxadiazole‐ligand binder entity joined to a ligand for von Hippel–Lindau (VHL) protein [30]. We used this molecule (#11) to target HDAC4 and achieve OXPT resensitization in FBXW7‐mutated CRC. First, we demonstrated that #11 was effective in degrading HDAC4 in both WT and *FBXW7* ^−/−^ HCT‐116 cells, but it was not selective with respect to other class IIa HDACs (Fig. 4G). Moreover, compound #11 succeeded in restoring HDAC4 degradation in OXPT‐treated *FBXW7* ^−/−^ HCT‐116 cells (Fig. S4C). Importantly, #11 did not induce degradation of class I HDACs (Fig. 4G) and did not impair class I and IIb HDAC activity, while it led to degradation of all class IIa HDACs and strongly abrogated class IIa HDAC activity (Fig. 4H). Although the degradation of both HDAC4 and HDAC5 may be desirable given their regulation by OXPT, we would have preferred to adopt a pharmacological approach that excludes HDAC7, which is expressed at lower levels than HDAC4 and HDAC5 but is still present in CRC. However, due to the large sequence homology of the class IIa deacetylase domain, to date the inhibitors of class IIa HDACs are not or only slightly selective toward the different members [71].

From a biological point of view, #11 triggered caspase activation (Fig. 4I and Fig. S4D) and cell death (Fig. 4J) in OXPT‐treated HCT‐116 *FBXW7* ^−/−^ cells at levels comparable to WT cells, thus reaching almost complete resensitization. Staurosporine (STS) treatment was used as a control and was equally effective in WT and *FBXW7* ^−/−^ cells, demonstrating that the refractoriness to OXPT observed in KO cells was not due to a general apoptotic defect (Fig. 4I). To exclude the presence of FBXW7‐independent off‐target effects due to the KO generation with Cas9, FBXW7 expression was silenced with esiRNAs in HT‐29 cells (Fig. S4E). Again, cotreatment with OXPT and #11 boosted activation of apoptosis, which was more pronounced in cells in which FBXW7 expression was silenced (Fig. S4F). Thirdly and more importantly, treatment with #11 almost completely prevented the arising of OXPT‐resistant colonies in *FBXW7* ^−/−^ HCT‐116 cells (Fig. 4K and Fig. S3G).

To prove that this effect was not limited to HCT‐116 and HT‐29 cells, we stably expressed FBXW7^R505C^ in SW620 cells (Fig. 2C and Fig. S4H). FBXW7^R505C^ overexpression in SW620 cells decreased OXPT‐induced cytotoxicity (Fig. S4H,I) and #11 restored sensitivity to OXPT also in SW620 FBXW7^R505C^ (Fig. S4H,J).

Combined chemotherapy treatments were designed to overcome single‐agent induced resistance. In CRC, FOLFOX (Folinic acid, fluorouracil 5‐FU, and oxaliplatin) is routinely used is adjuvant or neoadjuvant regimen for advanced CRC [1]. FBXW7 deficiency was reported to decrease sensitivity both to 5‐FU and OXPT [77]. We confirmed the mild refractoriness of *FBXW7‐*depleted HCT‐116 cells to FOX (5‐FU + OXPT) treatment, that was overcome by #11 co‐treatment (Fig. S4K).

Finally, to demonstrate *in vivo* the successful resensitization to OXPT achieved with forced degradation of HDAC4, HCT‐116 *FBXW7* ^−/−^ were xenografted in nude mice. About 5 mg·kg^−1^ OXPT was administered twice a week with intraperitoneal injections in combination with 5 and 10 mg·kg^−1^ of #11. A strong and significant antitumor effect was already observed with the administration of 5 mg·kg^−1^ of #11; therefore, this dosage was used in following experiments (Fig. S4L). Treatment with #11 alone (Fig. 4L) or with OXPT alone (Fig. 4M) resulted in a modest reduction in subcutaneous tumor growth, while concomitant treatment with 5 mg·kg^−1^ OXPT and 5 mg·kg^−1^ of #11 resulted in a significant reduction in subcutaneous tumor size (Fig. 4M) and weight (Fig. 4N). It is important to note that #11 induced efficient degradation of HDAC4 *in vivo* (Fig. 4O). Furthermore, the two mice in which HDAC4 degradation was less effective (mice number 13 and 15) were those that showed residual growth of the tumor (Fig. 4O).

Overall, these data showed that HDAC4 depletion or class IIa HDACs forced degradation restored sensitivity to OXPT‐containing chemotherapy in FBXW7‐depleted or mutated CRC cells and in a subcutaneous murine xenograft model.

### 3.6 HDAC4 silencing or class IIa HDACs forced degradation with #11 in *FBXW7* ^−/−^ HCT‐116 cells restore the transcriptome of CRC responsive to OXPT

**Fig. 5:**
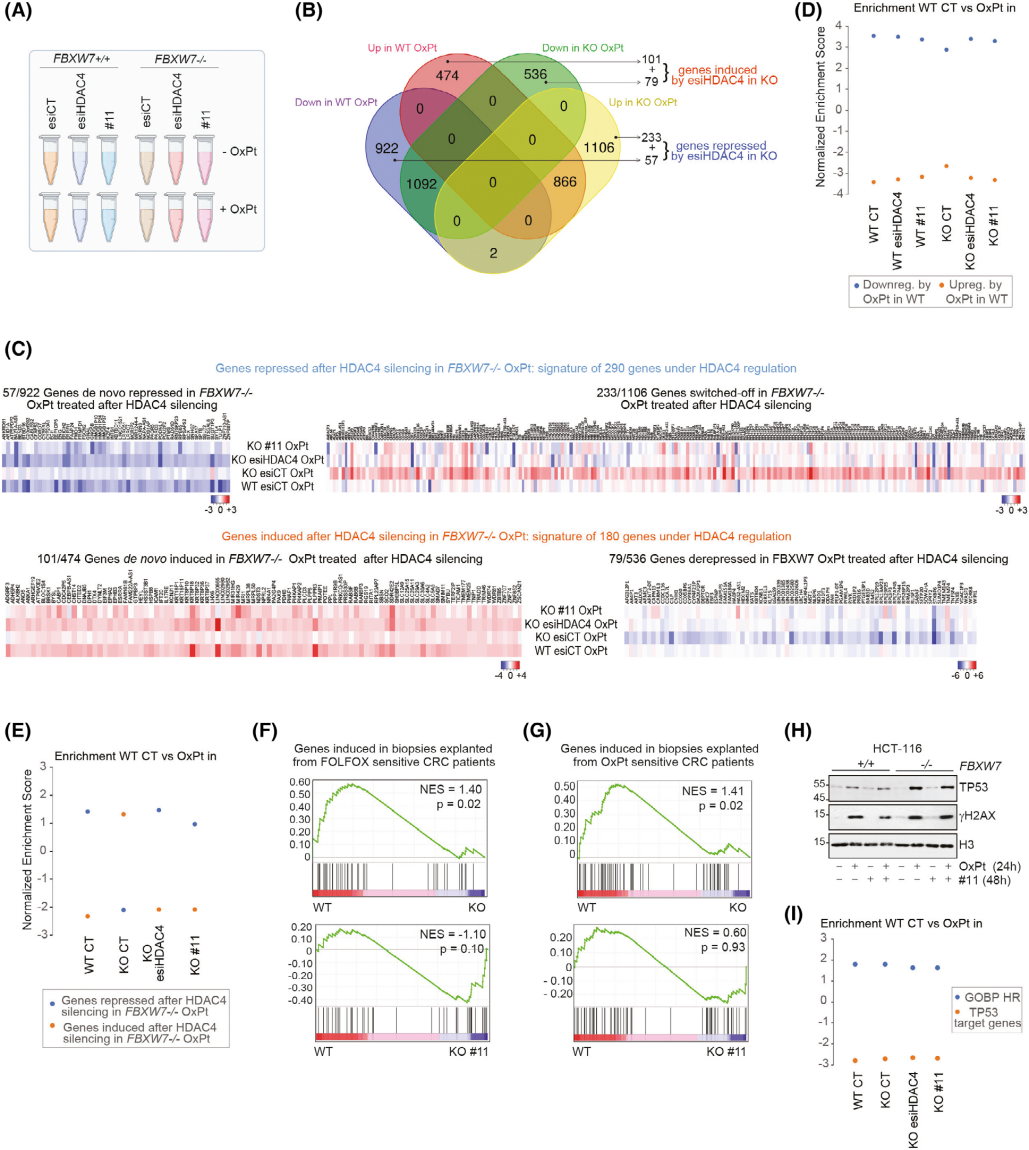
HDAC4 silencing or forced degradation with PROTAC compound #11 in *FBXW7* ^−/−^ HCT‐116 cells restore the transcriptome of cellular models and CRC patients responsive to OXPT. (A) Schematic representation of the samples and treatments subjected to RNA‐seq analysis. Cells were silenced for 48 h and then treated or not for 24 h with 50 μm OXPT. (B) Venn diagram reporting the number of differentially expressed genes in WT and *FBXW7* ^−/−^ HCT‐116 cells after 24 h of treatment with OXPT. The subgroup of genes perturbed by HDAC4 silencing are indicated. (C) Heatmap showing the relative expression levels (Log2Fc) of HDAC4 regulated genes in the indicated samples in respect to *FBXW7* ^−/−^ untreated cells. (D) Histogram reporting the NES of GSEA performed by comparing the WT untreated cells with the indicated OXPT‐treated samples and using as gene sets the genes repressed (blue) or induced (orange) by OXPT in WT cells. (E) Histogram reporting the NES of GSEA performed by comparing the WT untreated cells with the indicated OXPT‐treated cells (in *x*) and using as gene sets the genes repressed (blue) or induced (orange) by esiHDAC4 in OXPT‐treated *FBXW7* ^−/−^ cells. (F) GSEA plots obtained by using as gene set the signature of genes induced in biopsies explanted from FOLFOX sensitive CRC patients in respect to resistant ones (GSE72970). The loss of significance compared to the WT, which was achieved with the OPt+#11 cotreatment in FBXW7^−/−^ cells, indicates that the KO cells no longer differ significantly from the WT cells as a result of the cotreatment. (G) GSEA plots obtained by using as gene set the signature of genes induced in biopsies explanted from FOLFOX sensitive PDX in respect to resistant ones (GSE28691). The loss of significance compared to the WT, which was achieved with the OPt+#11 cotreatment in FBXW7^−/−^ cells, indicates that the KO cells no longer differ significantly from the WT cells as a result of the co‐treatment. (H) Immunoblot analysis in the indicated HCT‐116 cells, treated with 50 μm OXPT and 1 μm #11, as indicated. (I) Histogram reporting the NES of GSEA performed by comparing the WT untreated cells with the indicated OXPT‐treated cells and using as gene sets the GOBP_Homologous recombination (blue, M23395) and TP53 target genes (orange, M4391) in OXPT‐treated *FBXW7* ^−/−^ cells.

Analysis of the transcriptome of CRC models found to be refractory or responsive to chemotherapy reveals plastic adaptations and activation of death mechanisms or survival pathways [78]. RNA‐seq was performed on WT and *FBXW7* ^−/−^ HCT‐116 cells in which HDAC4 expression was suppressed, or not, with esiRNAs or #11 for 48 h and treated, or not, with OXPT for the last 24 h (Fig. 5A). RNA‐seq analysis of WT and *FBXW7* ^−/−^ HCT‐116 cells showed a profound difference in response to OXPT treatment (Fig. 5B). Of all the genes that were significantly dysregulated in WT and *FBXW7* ^−/−^ HCT‐116 cells, only 42.7% of the repressed genes and 35.4% of the induced genes were significantly regulated in both WT and *FBXW7* ^−/−^ cells (Fig. 5B). The differential transcriptional response of the two cell lines to OXPT treatment was confirmed by detection of clear transcriptional repression in the WT cells (60.1% of dysregulated genes were repressed by OXPT) and transcriptional induction in *FBXW7* ^−/−^ cells (55% of dysregulated genes were induced by OXPT) (Fig. 5B). Importantly, knocking down HDAC4 in *FBXW7* ^−/−^ cells significantly attenuated these differences (Fig. 5B). Specifically, the analysis revealed a signature of 470 genes under the direct influence of HDAC4, of which 290 were repressed and 180 were induced by HDAC4 silencing in *FBXW7* ^−/−^ OXPT‐treated cells (Fig. 5B and heatmap in Fig. 5C). GSEA analysis shown in Fig. 5D confirmed that the silencing of HDAC4 or its forced degradation was effective in attenuating the differences between the transcriptional responses to OXPT in WT cells compared to *FBXW7* ^−/−^ cells (Fig. 5D). In addition, GSEA evidenced that the signature of 470 genes under the influence of HDAC4 successfully segregated the transcriptional response to OXPT between WT and *FBXW7* ^−/−^ cells (Fig. 5E). Overall, this transcriptomic analysis suggests that the lack of degradation of HDAC4 in *FBXW7* ^−/−^ cells is one of the key factors for the dichotomous transcriptional response to OXPT between WT and *FBXW7* ^−/−^ cells as the knockdown of HDAC4 or its forced degradation achieved with #11 resulted in a resetting of the transcriptome of OXPT‐resistant HCT‐116 *FBXW7* ^−/−^ cells, making them closer to WT OXPT‐sensitive cells (Fig. 5D,E). Functional annotation of genes induced after HDAC4 silencing in *FBXW7* ^−/−^ cells evidenced the transcriptional activation of the apoptotic program (Fig. S5A,B). The transcriptional derepression achieved with HDAC4 silencing (Fig. S5C,D) or #11 treatment (Fig. S5B) of *PMAIP1* encoding NOXA and of *BBC3* encoding PUMA is relevant as these BH3‐only pro‐apoptotic proteins act indirectly by neutralizing anti‐apoptotic proteins and were reported to be induced after Vorinostat treatment thus sensitizing FBXW7 mutated cancers to pro‐apoptotic stimuli [79]. Functional annotation of genes repressed after HDAC4 silencing in *FBXW7* ^−/−^ cells evidenced the transcriptional repression of OXPHOS genes (Fig. S5E,F), counteracting the activation of the oxidative phosphorylation pathway previously reported in FBXW7 deficient tumor cells [27].

To understand whether the signatures obtained have clinical value, we applied GSEA to the transcriptome (GSE72970) of biopsies from CRC patients treated with FOLFOX and divided into resistant (*n* = 12) and sensitive (*n* = 20) cases. GSEA confirmed that the transcriptome of HCT116 *FBXW7* ^−/−^ cells resembled that of resistant patients, but this similarity was lost after treatment with #11 (Fig. 5F). By restricting the analysis to CRC PDX samples treated with OXPT alone (GSE28691), it was confirmed not only that the transcriptome of *FBXW7* ^−/−^ cells clearly resembled that of resistant PDX, but also that treatment with #11 restored a transcriptome similar to that of PDX sensitive to OXPT (Fig. 5G).

In summary, the signature describing transcriptional regulation controlled by HDAC4 may be important *in vivo* for predicting sensitivity to OXPT and in turning on the cell death program. It is important to note that the resistance of *FBXW7* ^−/−^ cells to OXPT, as well as their resensitization to platinum after forced degradation of HDAC4, did not depend on differences in DNA damage response (quantified by γH2AX levels, Fig. 5H) or differences in the immediate cellular responses to such damage (quantified by p53 levels in Fig. 5H and activation of p53 and homologous‐recombination signatures in Fig. 5I), suggesting that the epigenetic function of HDAC4 may be involved in regulating the survival and death response to OXPT.

### 3.7 The forced degradation of HDAC4 achieved with #11 restored sensitivity to OXPT in CRC PDOs bearing FBXW7 LOF

CRC PDOs responses to OXPT‐containing chemotherapy were reported to correlate with clinical responses in the case of resections of primary tumors [80, 81], while both accurate [82] and inaccurate [83] predictions of clinical response were reported in the case of metastatic disease.

To test the translational potential of our combined treatment (OXPT+#11), we selected two PDOs bearing FBXW7 LOF mutations and a heterogeneous genetic background: PDM‐96 (APC^R232*,E1397*^; KRAS^G12D,wt^; SMAD4^V350F,wt^; FBXW7^R505C,wt^) and PDM‐257 (APC^Q1378*,wt^; KRAS^G12S,wt^; TP53^C176Y,wt^; FBXW7^R222*,S596F^). PDM‐96 bears the monoallelic missense mutation (R505C) of FBXW7 which we demonstrated to have a dominant‐negative effect on HDAC4 degradation (Fig. 2C). PDM‐257 bears a monoallelic nonsense mutation of FBXW7 and a monoallelic variant of uncertain significance (S596F) of FBXW7. Co‐immunoprecipitation experiments evidenced that while the FBXW7^R505C^ mutation prevents the interaction between FBXW7 and HDAC4, the interaction between FBXW7^S596F^ and HDAC4 was equal to the WT (Fig. S6A). Interestingly, FBXW7^S596F^ migrates faster in SDS/PAGE than the WT, suggesting that serine 596 is likely an important phosphorylation site of FBXW7 (Fig. S6A). Further studies are required to understand the implications of this mutation and its putative effect on FBXW7 phosphorylation. These data suggest that: (a) FBXW7^S596F^ mutation has no dominant‐negative effects on HDAC4 degradation; (b) ectopic expression of FBXW7 WT in PDM‐257 should rescue the loss of function of FBXW7.

**Fig. 6:**
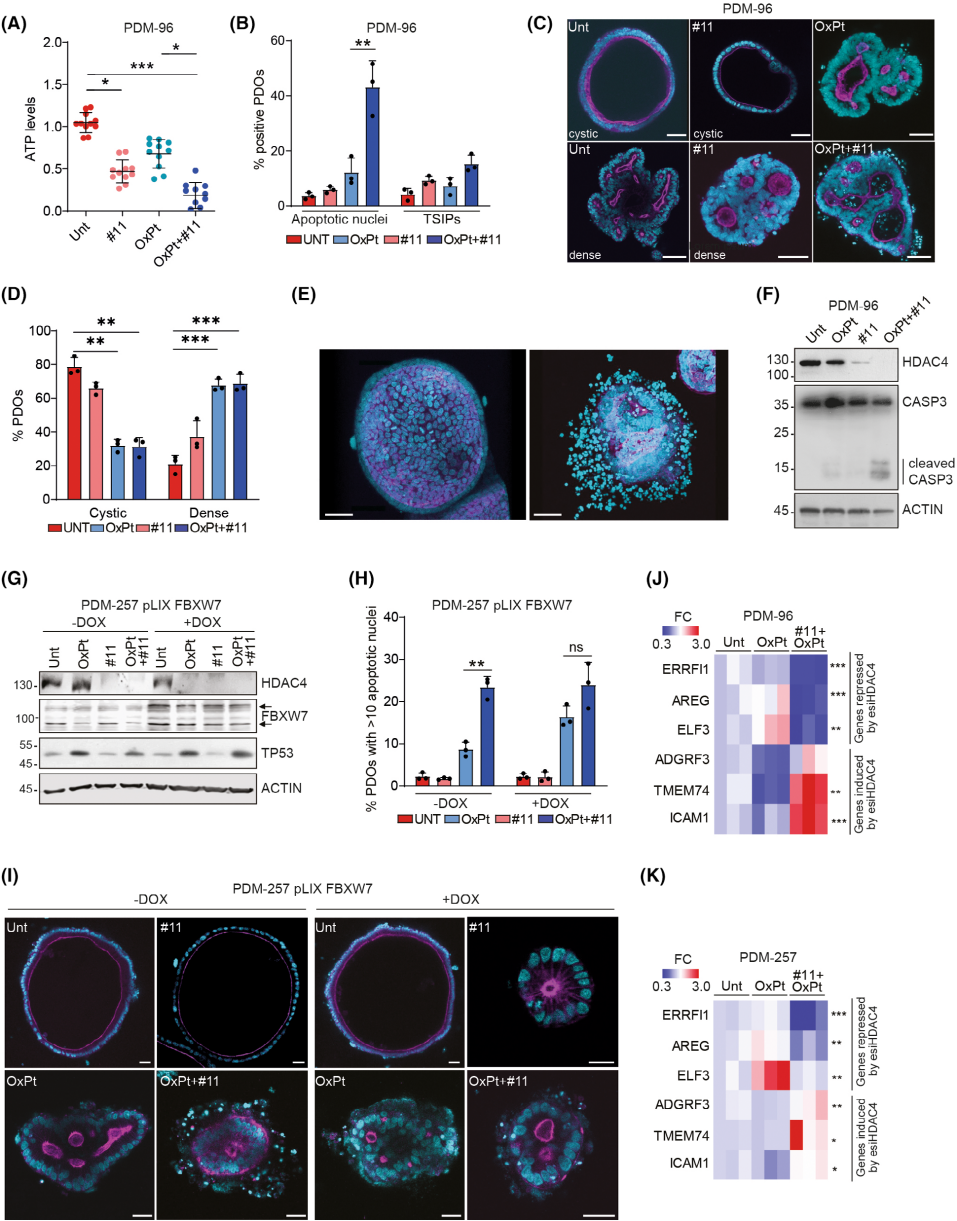
The forced degradation of HDAC4 achieved with PROTAC #11 restored sensitivity to OXPT in CRC PDOs bearing FBXW7 LOF. (A) Scatter plot reporting ATP relative levels in PDM‐96, treated with the indicated compounds for 96 h. Means, SD and statistics (Dunn's multiple comparison test) are indicated. *n* = 11. (B) Histogram reporting the percentage of PDOs showing apoptotic nuclei (> 10 per PDO) and inverted polarity (TSIP) after 96 h of treatment with the indicated compounds of PDM‐96. Data are represented as means ± standard deviation, *n* = 3 (for each biological replicate at least 21 PDOs were analyzed). Dunn's multiple comparison test. (C) Representative confocal images of equatorial sections of PDM‐96 treated for 96 h with the indicated compounds. Confocal images are shown in pseudocolors: cyan = DNA, magenta = actin cytoskeleton. Scale bar = 50 μm. (D) Histogram reporting the percentage of PDOs showing cystic or dense morphology with the indicated treatments. *n* = 3. (E) 3D reconstruction of the series of optical sections acquired by confocal microscopy in the PDM‐96, untreated (left) or treated OXPT+#11 (right). The combined treatment leads to the accumulation of apoptotic bodies. Scale bar = 65 μm. (F) Immunoblot analysis of the indicated proteins by using lysates generated from PDM‐96 grown in 3D for 3 days and treated as indicated for 96 h. Cleaved caspase 3 is indicated. *n* = 3. (G) Immunoblot analysis of the indicated proteins by using lysates generated from PDM‐257 induced (+DOX) or not (−DOX) to express FBXW7 during the 96 h of treatment with the indicated compounds. The two arrows indicate the two forms of FBXW7, the one encoded by the endogenous allele (596F, which runs faster) and the WT one expressed with the DOX inducible lentiviral plasmid. *n* = 3. (H) Histogram reporting the percentage of PDOs showing apoptotic nuclei (> 10 per PDO) after 96 h of treatment with the indicated compounds of PDM‐257. Data are represented as means ± standard deviation, *n* = 3 (for each biological replicate at least 15 PDOs were analyzed). ANOVA and Bonferroni post‐test. (I) Representative confocal images of equatorial sections of PDM‐257, induced to express FBXW7 WT (+DOX) or not (−DOX) and treated for 96 h with the indicated compounds. Scale bar = 50 μm. (J, K) Heatmap representing the relative mRNA expression levels of the indicated genes in PDM‐96 (J) and PDM‐257 (K), treated as explained in Fig. 6B. Statistics is indicated and is relative to comparison between OXPT and OXPT+#11 treated samples. *n* = 3. In B, D, H data are represented as means ± SD.

We first tested the effect of combined treatments versus single treatments in PDM‐96. OXPT+#11 co‐treatment impaired cell viability (Fig. 6A) and led to cell death (Fig. 6B). Microscopic inspection of treated PDOs (Fig. 6C) and 3D reconstructions (Fig. 6E) revealed minimal accumulation of apoptotic nuclei when treated with only OXPT or #11 and a significant accumulation of apoptotic bodies after co‐treatment (Fig. 6C). It is important to note that the combined treatment increased the percentage of PDOs displaying inverted polarity (TSIPs) (with the apical pole oriented outwards, Fig. S5B), although not significantly from a statistic point of view. This condition was previously reported to associate with chemoresistance [84, 85]. However, as highlighted in Fig. S5B, polarity reversal was also observed in organoids characterized by the accumulation of apoptotic nuclei after cotreatment with OXPT and #11 (Fig. S6B). Further studies will be required to decipher the signaling pathways controlling this cellular reorganization upon treatment. Moreover, OXPT treatment led to the acquisition by CRC PDOs of a predominant dense and compact, not cystic, morphology typical of poorly differentiated tumors [86], which was minimally altered by #11 co‐treatment (Fig. 6D). In parallel, immunoblot analysis showed the efficient degradation of HDAC4 obtained after treatment with #11 and the processing of caspase 3 in the OXPT+#11 treated sample (Fig. 6F). Mitochondrial fission and progressive dissipation of mitochondrial membrane potential (Δψm) confirmed the induction of apoptosis in PDM‐96 cotreated with OXPT and #11 (Fig. S6C).

Next, PDM‐257 was engineered to re‐express wild‐type FBXW7 (Fig. 6G) in an inducible manner after treatment with doxycycline (DOX). Immunoblot analysis confirmed the degradation of HDAC4 in OXPT‐treated samples following re‐expression of FBXW7 and provided further evidence that FBXW7^S596F^ migrates faster in SDS/PAGE compared to WT (Fig. 6G). OXPT treatment led to elevated p53 protein levels, consistent with its activation [87], and this effect was observed equally in both the absence and presence of DOX‐induced FBXW7 expression (Fig. 6G). Importantly, re‐expression of FBXW7 increased sensitivity to OXPT (Fig. 6H). In the absence of FBXW7 re‐expression, cotreatment with OXPT+ #11 restored sensitivity to OXPT by inducing apoptosis (Fig. 6H,I).

Finally, the resensitization to OXPT achieved by forced degradation of HDAC4 determined a change in the transcript levels of genes modulated by HDAC4 silencing in HCT‐116 *FBXW7* ^−/−^, confirming the consistency of the model (Fig. 6J,K).

Overall, forced degradation of HDAC4 successfully resensitized to OXPT two PDOs carrying mutations of FBXW7.

### 3.8 The resetting of H3K27acetylome and of SEs landscape control OXPT sensitivity

**Fig. 7:**
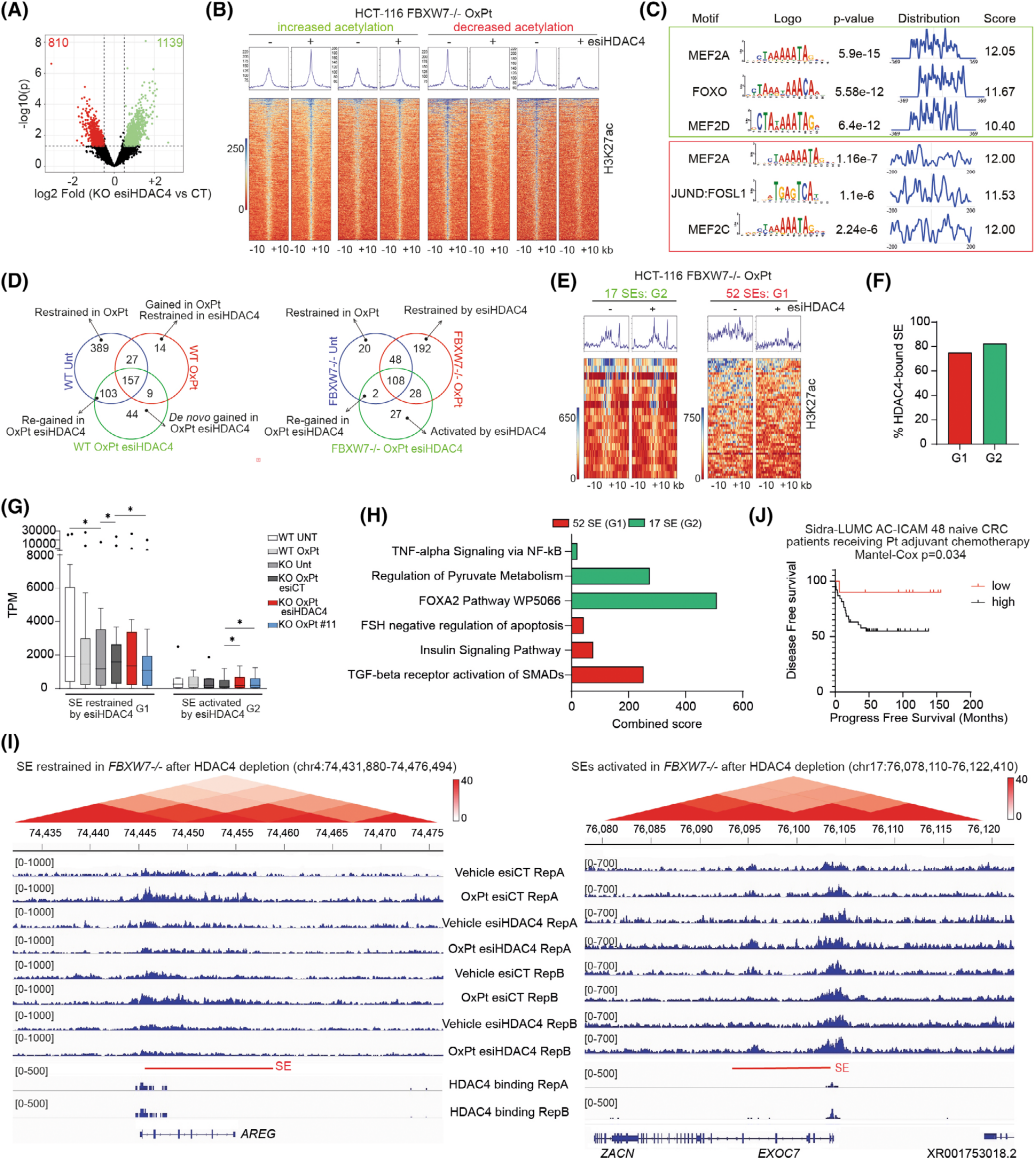
The resetting of H327 acetylome and of SEs landscape control OXPT sensitivity. (A) Volcano plot showing the differential H3K27 acetylated peaks by comparing esiHDAC4 and esiCT in *FBXW7* ^−/−^ HCT‐116 cells. *n* = 2. (B) Heatmap of H3K27ac reads distribution within 20 kb from the center of the H3K27ac peaks showing increased acetylation (indicated in green) or decreased acetylation (indicated in red) after HDAC4 silencing. (C) Motif enrichment analysis within genomic loci characterized by increased H3K27 acetylation (green box) or decreased acetylation (red box) after HDAC4 silencing in *FBXW7* ^−/−^ cells. (D) Venn diagram reporting the number of SEs identified in WT and *FBXW7* ^−/−^ HCT‐116 cells, silenced or not for HDAC4 and treated or not for 24 h with 50 μm OXPT. *n* = 2. (E) Heatmap of H3K27ac reads distribution within 20 kb from the center of the H3K27ac peaks in G1 and G2 groups of *bona fide* SEs. (F) Histogram reporting the percentage of HDAC4‐bound G1 and G2 SEs. (G) Tukey boxplots of the absolute expression levels of the mRNAs associated with G1 and G2 SEs. ANOVA and Dunn's multiple comparison test. Mean and 1st and 3rd quartile are indicated. (H) Histogram reporting the combined scores of the functional categories found enriched in the genes associated with G1 and G2 SEs. (I) ChIP‐seq H3K27ac levels in two representative genomic loci containing SEs belonging to G1 (left) and G2 (right) groups. Hi‐C data were from HCT‐116 cells [89]. HDAC4 peaks falling within SEs were previously identified [31]. (J) Kaplan–Meier curve of progression‐free survival based on the expression levels of the G1 signature of genes described in Fig. 7G in biopsies obtained from CRC patients from the Sidra‐LUMC AC‐ICAM cohort.

Integration of transcriptomic and epigenomic profiling in CRC has been shown to be effective in establishing predictive models for clonal evolution [21] and chemoresistance [88]. Class IIa HDACs control the cellular epigenetic state by organizing SEs under stress conditions and supporting the transcriptional response to counteract cell death stimuli [12, 31]. To obtain a picture of the epigenetic state of the response to OXPT, we profiled H3K27ac genome‐wide in HCT‐116 WT and *FBXW7* ^−/−^ cells treated or not treated with OXPT and in which HDAC4 expression was repressed or not with esiRNAs (characterized in Fig. 4B). The H3K27 acetylome from two biological replicates was compared and CHIPIN was used for normalization [40]. Despite the profound transcriptional switch observed in the OXPT‐treated samples, no overall changes in H3K27ac levels were detected under the six conditions compared (Fig. S7A). However, PCA analysis based on H3K27 acetylome clearly identified the different treatment groups and clustered WT and *FBXW7* ^−/−^ samples (Fig. S7B). This suggested that local and nonglobal epigenetic differences in H3K27ac were responsible for the observed phenotypes. To identify the H3K27 acetylome under the control of HDAC4, we applied DiffBind to *FBXW7* ^−/−^ OXPT‐treated samples silenced of not for HDAC4. DiffBind identified 1949 genomic loci with significant altered HK27ac levels after HDAC4 knockdown (|Fc| > 1.5, *P* < 0.05, Fig. 7A,B). In supporting the role of class IIa HDACs as an acetyl‐lysine reader [12, 13], these regions are divided more or less equally between deacetylated and hyperacetylated regions after HDAC4 knockdown, making it plausible that HDAC4 supervises the acetylation status of the identified genomic regions. As an indirect confirmation, analysis of motif enrichment identified well‐known partners (MEF2, AP1, FOXOs) [31, 90] of HDAC4 among the binding sites found enriched in these 1949 genomic regions (Fig. 7C).

Clusters of enhancers defined as super‐enhancers (SE) were reported to control cell fate [91] and were previously identified as a privileged docking site for HDAC4 [12]. To identify active enhancers, we mapped H3K4me1 genome‐wide (Fig. S7C) in HCT‐116 WT and *FBXW7* ^−/−^ cells treated or not treated with OXPT and in which HDAC4 expression was repressed or not with esiRNAs, and we intersected H3K27ac enriched peaks with H3K4me1 peaks. We then applied the Rank Ordering of Super‐Enhancers (ROSE) algorithm [91] to rank the identified enhancers accordingly to H3K27ac signals and stitch H3K27ac peaks together within 12.5 kb to identify *bona fide* SEs [92] (Fig. S7D). Only *bona fide* SEs identified in both the biological replicates were considered (Fig. S7E). OXPT caused a decrease in the number of SEs in WT cells and a significant increase of SEs in *FBXW7* ^−/−^ cells (Fig. 7D, Fig. S7D and Table S3). Of the large number of SEs lost after OXPT treatment in HCT‐116 FBXW7 WT cells, only a minimal proportion of them was restored by HDAC4 silencing, confirming that OXPT‐induced HDAC4 degradation weakened its epigenetic regulatory effect (Fig. 7D). In contrast, silencing of HDAC4 restrained many SEs in OXPT‐treated *FBXW7* ^−/−^ cells and activated some of them (Fig. 7D). Fifty‐two genomic regions (defined as group 1, G1) and 17 genomic regions (defined as group 2, G2) corresponding to *bona fide* SEs were identified by DiffBind to be respectively significantly H3K27 deacetylated (group G1) and hyperacetylated (group G2) after HDAC4 knockdown in HCT‐116 *FBXW7* ^−/−^ cells treated with OXPT (Fig. 7E, Table S4 and their genomic distribution with respect to TSS in Fig. S7F). 75% and 82%, respectively, of G1 and G2 SEs were previously characterized by us [31] to be directly bound by HDAC4 (Fig. 7F). Available Hi‐C data from HCT‐116 cells [89] were interrogated with these two sets of SEs to isolate the relevant topological‐associated domains (TADs) and to identify by using GREAT 4.0 [93] the associated genes that fell within these TADs (Table S5). RNA‐seq data (Fig. 5) were interrogated to quantify the expression of these genes in the different experimental conditions tested. Integrated epigenomic and transcriptomic profiling revealed that silencing HDAC4, or inducing its degradation with compound #11 in OXPT‐treated *FBXW7* ^−/−^ cells, resulted in two distinct outcomes: (a) a reduction in mRNA levels of genes linked to SEs that become repressed following HDAC4 silencing (G1); and (b) an upregulation of mRNA levels for genes associated with SEs that undergo hyperacetylation upon HDAC4 silencing (G2) (Fig. 7G). Furthermore, HDAC4 knockdown was shown to enhance H3K27ac deposition and activate genes with low basal expression (low TPM), while concurrently dampening H3K27ac levels and repressing genes with high basal expression (high TPM), consistent with our previous findings (Fig. 7G) [34]. Functional enrichment of genes associated with G1 and G2 SEs confirmed the activation of the apoptotic program after HDAC4 knockdown and the reprogramming of glucose metabolism (Fig. 7H), in accordance with RNA‐seq data. Four representative loci are shown in Fig. 7I, Figs S7G and S8A, this latter displaying the integration of H3K27ac and H3K4me1 signals. ChIP‐seq confirmed the epigenetic program controlling the transcriptional induction of pro‐apoptotic genes *PMAIP1/NOXA* and *BBC3/PUMA* (Fig. S5B) after HDAC4 knockdown (Fig. S8B).

To evaluate the clinical relevance of gene signatures associated with G1 and G2 SEs, we analyzed CRC biopsy data from the TCGA dataset. Patient samples were stratified based on FBXW7 status—loss‐of‐function (LOF) mutations versus wild‐type (WT)—and whether they had received OXPT‐based chemotherapy (Table S6). For each of the four resulting groups, the median expression (*z*‐score) of the gene signatures was plotted per patient (Fig. S8C). The analysis revealed a significant upregulation of genes linked to G1 SEs in FBXW7‐LOF patients compared to WT, with this effect further amplified by OXPT treatment. These findings highlight that the transcriptional response to OXPT mediated by G1 SEs is preserved in FBXW7‐LOF tumors.

Finally, we hypothesized that the absence of epigenetic regulation over G1‐associated super‐enhancers (SEs) could intrinsically influence sensitivity or resistance to platinum (Pt)‐based therapies, regardless of FBXW7 mutational status. To assess the prognostic relevance of this gene signature, we analyzed transcriptomic profiles and progression‐free survival data from colorectal cancer (CRC) patient biopsies in the Sidra‐LUMC AC‐ICAM cohort [94], collected postsurgical resection and prior to Pt‐based adjuvant treatment. As illustrated in Fig. 7J, patients exhibiting low expression of the G1 SE‐associated gene signature (below the first quartile) showed a more favorable response to platinum‐based therapy, highlighting the potential predictive value of this transcriptional program. These findings suggest that the HDAC4‐regulated epigenetic signature associated with G1 SEs may serve as a prognostic indicator.

### 3.9 Development of an epigenetic fluorescent lentiviral reporter to track the response to OXPT+#11 cotreatment

Lentiviral reporter plasmids have been successfully adopted to measure the function of candidate regulatory sequences [95].

**Fig. 8:**
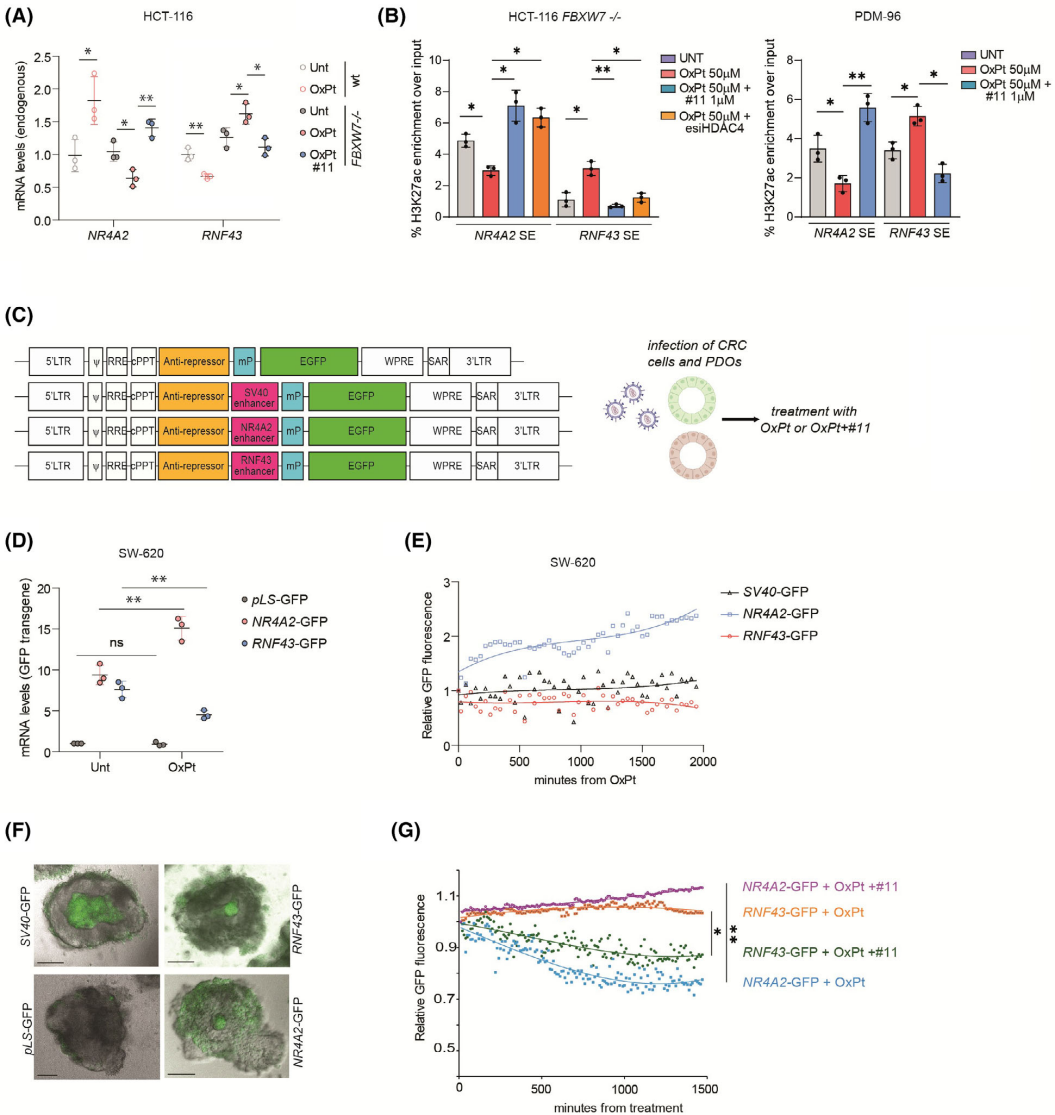
Characterization of an epigenetic fluorescent lentiviral reporter to track the response to OXPT + #11 combination treatment at the single cell level. (A) Dot plot of the mRNA expression levels of *NR4A2* and *RNF43* in the indicated cells and treatments. Mean and SD are indicated. Dunn's multiple comparison test. *n* = 3. (B) qPCR to test H3K27ac enrichment within the regulative regions selected for the two SEs investigated in the indicated cells or PDO, treated as indicated. *n* = 3. Data are represented as means ± SD. (C) Scheme of the lentiviral reporter generated to infect CRC cells and PDM‐96 and to score the activity of *NR4A2* and *RNF43* regulative regions within SE. (D) Dot plot of the EGFP mRNA expression levels driven by pLS or pLS‐RNF43 and pLS‐NR4A2 lentiviral plasmids in the indicated cells subjected or not for 24 h to treatment with OXPT 50 μm. Mean and SD are indicated. Dunn's multiple comparison test. *n* = 3. (E) Scatter dot plot showing the GFP fluorescence levels of SW620 cells expressing the indicated fluorescent reporters and treated for the indicated time with OXPT 50 μm. Third order polynomial regression curve is shown. *n* = 9. (F) Representative equatorial sections of PDM‐96 expressing the indicated fluorescent reporters. Brightfield and GFP fluorescence are overlayed. Scale bar = 37.5 μm. (G) Scatter dot plot showing the GFP fluorescence levels in PDM‐96 expressing the indicated fluorescent reporters and treated for the indicated time with OXPT 50 μm. Third order polynomial regression curve is shown. *t*‐test of pairwise comparison of last time point. *n* = 9.

Here we developed fluorescent lentiviral reporters to measure the activity of the two groups of HDAC4‐controlled SEs in CRC samples treated with OXPT and OXPT+#11, to: (a) quantify the activity of these regulatory elements; (b) test the function of these regulatory regions as markers of response to OXPT and OXPT+#11. We selected the SEs controlling the expression of *RNF43* (belonging to group 1 of SEs restrained by HDAC4 inhibition, Fig. 7E) and *NR4A2* (belonging to group 2 of SEs activated by HDAC4 inhibition, Fig. 7E). These two loci were selected as both are important for the biology of CRC: *RNF43* encodes an E3‐ligase involved in the ubiquitylation of Frizzled and blockage of Wnt [96] and indirectly inhibits p53 signaling [97]; *NR4A2* encodes a transcription factor of the Orphan Nuclear Receptor 4A family reported to exhibit oncogenic properties when it accumulates in the nucleus [98] but has possible pro‐apoptotic functions outside the nucleus [99]. *RNF43* was suppressed by OXPT in *FBXW7* ^+/+^ HCT‐116 cells, whereas it was induced by OXPT in *FBXW7* ^−/−^ cells and HDAC4 forced degradation blocked its induction (Fig. 8A). *NR4A2* exhibited the opposite regulation: it was induced by OXPT in WT cells, repressed in KO cells, and derepressed by inhibition of HDAC4 (Fig. 8A). We isolated the core region of these bona fide SEs, each corresponding to the region found to be bound by HDAC4 [31]. Using qChIP, we confirmed H3K27 deacetylation of the core region controlling *RNF43* expression after HDAC4 silencing or degradation in HCT‐116 *FBXW7* ^−/−^ cells and in PDM‐96 (Fig. 8B). Similarly, we confirmed the increased acetylation of the regulatory element of *NR4A2* after HDAC4 inhibition (Fig. 8B). Lentiviral reporter plasmids were created by cloning these core enhancer elements between the antirepressor element #40 (which works as an enhancer‐blocking element) [100, 101] and a minimal promoter (mP) driving the expression of EGFP. As a positive control, the enhancer of SV40 was used. Lentiviral particles were then used to infect SW620 cells (*FBXW7* ^+/+^, which were selected in place of HCT‐116 as these latter have a biallelic loss‐of‐function mutation of RNF43 [96]) and PDM‐96 (the CRC PDO with *FBXW7* ^*505C*^ mutation, described in Fig. 6), which were then treated with OXPT and OXPT+#11 (Fig. 8C). The regulatory elements selected for *NR4A2* and *RNF43* behaved as enhancers as they triggered the expression of EGFP in SW620 cells (Fig. 8D). Moreover, OXPT increased the activity of *NR4A2* enhancer while it decreased that of *RNF43* (Fig. 8D,E), similarly to what was observed by measuring endogenous mRNA in HCT‐116 cells (Fig. 8A). In PDM‐96, the expression of EGFP driven by *RNF43* and *NR4A2* enhancers was strong in the cells surrounding the lumen or in direct contact with matrix, in contrast to the SV40 enhancer which induced strong expression only in the cells surrounding the lumen (Fig. 8F). Finally, time‐lapse video microscopy of single cells showed that cotreatment with OXPT+#11 increased the activity of *NR4A2* and decreased the activity of *RNF43* enhancers (Fig. 8G and Videos S1–S4), restoring a similar epigenetic behavior as in *FBXW7* ^+/+^ samples. This is consistent with the ChIP‐seq data shown in Fig. 7 and the qChIP data shown in Fig. 8B.

In summary, these data show that these regulatory elements act as enhancers and that the epigenetic regulation observed in cells and organoids at endogenous loci is maintained on these reporters, which can thus serve as sensors to monitor the efficacy of the proposed cotreatments at the single cell level.

In conclusion, our work has uncovered a novel epigenetic mechanism underlying resistance to OXPT in CRC samples and patients. In addition, a signature with prognostic value has been identified that can be used to select *a priori* patients who will respond to adjuvant Pt‐based treatments.

## 4 Discussion

Overcoming innate or acquired chemoresistance is the main goal of precision oncology [102, 103]. Preventive genetic profiling of cancer patients based on association studies has significantly improved patient response to administered therapy [104, 105]. However, spatial transcriptomics has opened a new frontier by showing that the heterogeneity of tumor subclones and their evolution is not captured by the analysis of their genetic polymorphism, but rather by the analysis of their complex epigenetic and transcriptional plasticity [20, 21, 106]. These findings underscore the urgent need for epigenetic profiling of patients. Our work fits into this framework. Here, we identified impaired proteasomal degradation of the epigenetic regulator HDAC4 as one of the causes of refractoriness to OXPT in CRC models with *FBXW7* LOF. In this context, the accumulation of HDAC4 determined the reversal of the SE profile, which correlated with the acquisition of resistance to OXPT. Forced degradation of HDAC4 with a PROTAC compound restored the SE landscape of sensitive cells and led to successful resensitization to OXPT *in vitro* and *in vivo*.

FBXW7 is a tumor suppressor in CRC, with clearly defined mutational hotspots that correlate with the overexpression of proto‐oncogenes [22, 24, 26]. Given the promiscuity of FBXW7, it is unlikely that the accumulation of HDAC4 in mutated samples is the sole determinant of OXPT resistance. Indeed, several resistance mechanisms have been reported to be activated in *FBXW7* ^−/−^ samples, including metabolic reprogramming and epigenetic mechanisms leading to resistance to apoptosis [22, 25, 27, 59, 77]. However, knocking down or forcing degradation of HDAC4 is sufficient to induce resensitization to OXPT, demonstrating that HDAC4 plays a critical role. Partial degradation of HDAC5 was also observed and it is conceivable that dimerization of FBXW7 mediates the degradation of the two targets, HDAC4 and HDAC5, and enhances ubiquitylation by acting on two suboptimal substrates, as reported before for other targets [62, 63, 64]. The tendency of HDAC4 and HDAC5 to aggregate complicates these studies and the evaluation of their homo/heterodimerization [107]. The development of suitable reagents for HDAC5 will allow us to study its degradation in the future. Importantly, while further work needs to be done to identify the kinase responsible for priming phosphorylation on serine 219, phosphoproteomic studies have demonstrated the effective phosphorylation of HDAC4 on serine 215 [108]. According to our data, GSK3β is required to achieve the phosphorylation of HDAC4 at this site and allow its recognition by FBXW7.

The almost complete resensitization to OXPT achieved by #11 demonstrates that specific degradation of class IIa HDACs may be a good avenue to pursue. Off‐target degradation achieved with pomalidomide‐based PROTAC compounds has been reported, and new strategies have been developed to curb it [109]. Although the use of VHL‐based E3 ligase adaptor ligand (as in the case of compound #11) appears to minimize off‐target degradation, it cannot be completely ruled out [110]. Here, the good overlap between the epigenetic perturbation by HDAC4 silencing and its degradation with the PROTAC compound suggests that any off‐target effects of #11 do not play a key role in controlling this important resensitization phenotype. This strategy of forcing the degradation of class IIa HDACs requires optimization to ensure selective degradation of individual members; this is made more complex by the natural tendency of class IIa HDACs to aggregate [111] and heterodimerize [107].

Importantly, our work has identified an epigenetic program supervised by HDAC4 and controlling the expression of the pro‐apoptotic genes *PMAIP1* and *BBC3*, as well as two groups of SEs controlling the response to platinum. Moreover, our data have shown that HDAC4 keeps the turnover of H3K27ac under control, controlling acetylation/deacetylation rates in analogy to what has been previously reported for class I HDACs [14, 17, 18]. HDAC4 has been reported to form heterogeneous protein complexes [31, 112] and recent biochemical characterizations have shown that they are highly dynamic [113, 114]. The data obtained in this work highlight the need to characterize the different complexes that form on HDAC4 over time to understand the dynamics by which H3K27 acetylation turnover is regulated.

Finally, loss of epigenetic control of a limited number of SEs identified in this work (referred to as group 1 of SEs) successfully predicts the efficacy of Pt‐containing adjuvant therapies in patients, regardless of FBXW7 mutational status. Our future work will involve validating through spatial transcriptomic and epigenomic approaches whether this HDAC‐controlled signature is also effective as a marker for the emergence of subclones refractory to OXPT in susceptible patients.

## 5 Conclusion

These findings establish HDAC4 as a central epigenetic regulator of oxaliplatin resistance in FBXW7‐mutated colorectal cancer and demonstrate that targeted degradation of class IIa HDACs can reprogram the chromatin landscape, restoring drug sensitivity through super‐enhancer remodeling.

## Conflict of interest

The authors declare no conflict of interest.

## Author contributions

VT: investigation, formal analysis, writing – original draft. NG: investigation, formal analysis (Bioinformatics), writing – original draft. MC: investigation (IHC), formal analysis. RP: investigation, formal analysis (Bioinformatics). YC: investigation. FDE and FA: resources. MG: investigation (*in vivo* experiments). WWH: resources. GT: resources. CD: resources, investigation (*in vivo* experiments). CB: resources, writing – review and editing. LEX: resources, data curation, writing – review and editing. EDG: conceptualization, formal analysis, validation, funding acquisition, data curation, writing – review and editing.

## Supporting information


**Fig. S1.** Inhibition of homologous recombination (HR) repair leads to a reduction in HDAC4 protein levels across various cellular contexts.
**Fig. S2.** Identification of the E3 ligases involved in HDAC4 degradation through *in silico* and *in vitro* screenings.
**Fig. S3.** Characterization of FBXW7^−/−^ cells and FBXW7 R505C.
**Fig. S4.** HDAC4 forced degradation or silencing increased OXPT cytotoxicity.
**Fig. S5.** Identification of a signature of genes under the control of HDAC4.
**Fig. S6.** Characterization of PDOs.
**Fig. S7.** Characterization of the epigenetic response driven by HDAC4.
**Fig. S8.** Dissection of the epigenetic response driven by HDAC4.
**Fig. S9.** Original images used for the composition of the immunoblot panels in the main figures.
**Fig. S10.** Original images used for the composition of the immunoblot panels in the supplementary figures.
**Table S1.** Protein expression levels (*z*‐score) of HDAC4, HDAC5 and 365 E3 ligases available for the indicated 375 cancer cell lines of the Cancer Cell Line Encyclopedia.
**Table S2.** Characteristics of CRC patients whose biopsies were used for the TMA.
**Table S3.** .bed files of the SEs identified in HCT‐116 cells.
**Table S4.** .bed files of the SEs belonging to group 1 and 2 and those directly bound by HDAC4.
**Table S5.** Minimal signature of 116 genes associated to group 1 and 2 of SEs.
**Table S6.** TCGA sample ID of CRC patients bearing FBXW7 LOF.
**Table S7.** List and sequences of primers used for this study.
**Table S8.** Raw data for *in vivo* experiments.
**Video S1.** Time‐lapse video microscopy of PDM‐96 expressing pLS‐mP‐NR4A2‐EGFP treated with OXPT 20 μm at time 0.
**Video S2.** Time‐lapse video microscopy of PDM‐96 expressing pLS‐mP‐NR4A2‐EGFP treated with OXPT 20 μm + #11 1 μm at time 0.
**Video S3.** Time‐lapse video microscopy of PDM‐96 expressing pLS‐mP‐RNF43‐EGFP treated with OXPT 20 μm at time 0.
**Video S4.** Time‐lapse video microscopy of PDM‐96 expressing pLS‐mP‐RNF43‐EGFP treated with OXPT 20 μm + #11 1 μm at time 0.
**File S1.** Ethical documentation.

## Data Availability

All unique reagents generated in this study are available with a completed Materials Transfer Agreement. Raw data corresponding to RNA‐seq are uploaded with GEO accession GSE256188. Raw data corresponding to ChIP‐seq experiments are uploaded with GEO accession GSE270840 and GSE256187.
