# Supplementary material for: Class IIa HDACs forced degradation allows resensitization of oxaliplatin‐resistant FBXW7‐mutated colorectal cancer
Source: Mol Oncol. 2025 Oct 31;20(3):637–67. doi: 10.1002/1878-0261.70152 (PMC13042376; doi:10.1002/1878-0261.70152)
Supplement: Supplementary file 1 — Fig. S1. Inhibition of homologous recombination (HR) repair leads to a reduction in HDAC4 protein levels across various cellular contexts. Fig. S2. Identification of the E3 ligases involved in HDAC4 degradation through in silico and in vitro screenings. Fig. S3. Characterization of FBXW7−/− cells and FBXW7 R505C. Fig. S4. HDAC4 forced degradation or silencing increased OXPT cytotoxicity. Fig. S5. Identification of a signature of genes under the control of HDAC4. Fig. S6. Characterization of PDOs. Fig. S7. Characterization of the epigenetic response driven by HDAC4. Fig. S8. Dissection of the epigenetic response driven by HDAC4. Fig. S9. Original images used for the composition of the immunoblot panels in the main figures. Fig. S10. Original images used for the composition of the immunoblot panels in the supplementary figures. Table S1. Protein expression levels (z‐score) of HDAC4, HDAC5 and 365 E3 ligases available for the indicated 375 cancer cell lines of the Cancer Cell Line Encyclopedia. Table S2. Characteristics of CRC patients whose biopsies were used for the TMA. Table S3. .bed files of the SEs identified in HCT‐116 cells. Table S4. .bed files of the SEs belonging to group 1 and 2 and those directly bound by HDAC4. Table S5. Minimal signature of 116 genes associated to group 1 and 2 of SEs. Table S6. TCGA sample ID of CRC patients bearing FBXW7 LOF. Table S7. List and sequences of primers used for this study. Table S8. Raw data for in vivo experiments. Video S1. Time‐lapse video microscopy of PDM‐96 expressing pLS‐mP‐NR4A2‐EGFP treated with OXPT 20 μm at time 0. Video S2. Time‐lapse video microscopy of PDM‐96 expressing pLS‐mP‐NR4A2‐EGFP treated with OXPT 20 μm + #11 1 μm at time 0. Video S3. Time‐lapse video microscopy of PDM‐96 expressing pLS‐mP‐RNF43‐EGFP treated with OXPT 20 μm at time 0. Video S4. Time‐lapse video microscopy of PDM‐96 expressing pLS‐mP‐RNF43‐EGFP treated with OXPT 20 μm + #11 1 μm at time 0. File S1. Ethical documentation. [file MOL2-20-637-s001.zip › mol270152-sup-0023-DataS1.docx]

**Supplemental data**

**Class IIa HDACs forced degradation allows re-sensitization of oxaliplatin-resistant FBXW7-mutated colorectal cancer**

**Vanessa Tolotto^1#^, Nicolò Gualandi^1#^, Ylenia Cortolezzis^1^, Raffaella Picco^1^, Monica Colitti^2^, Francesca D’Este^1^, Mariachiara Gani^1^, Wayne W. Hancock^3^, Giovanni Terrosu^4^, Cristina Degrassi^5^, Francesca Agostini^1^, Claudio Brancolini^6^, Luigi Xodo^1^, Eros Di Giorgio^1*^**

*Running title: Epigenetic treatment of FBXW7 mutated colorectal cancer*

^1^Laboratory of Biochemistry, Department of Medicine, Università degli Studi di Udine, p.le Kolbe 4, 33100 Udine, Italy

^2^Dipartimento di Scienze agroalimentari, ambientali e animali, Università degli Studi di Udine, Udine, Italy

^3^Division of Transplant Immunology, Department of Pathology and Laboratory Medicine, Children’s Hospital of Philadelphia and Perelman School of Medicine, University of Pennsylvania, Philadelphia, Pennsylvania, USA

^4^Department of Medicine, Università degli Studi di Udine, Institute for Biomedicine, P.le Kolbe 4, Udine 33100, Italy

^5^MTTLab, Via Gallina 5 - 34122 Trieste - Italy

^6^Laboratory of Epigenomics, Department of Medicine, Università degli Studi di Udine, p.le Kolbe 4, 33100 Udine, Italy

# Co-first authorship

Correspondence: [eros.digiorgio@uniud.it](mailto:eros.digiorgio@uniud.it)

Lead contact: [eros.digiorgio@uniud.it](mailto:eros.digiorgio@uniud.it)

**Supplementary Tables**

**Supplementary Table 1.** Protein expression levels (z-score) of HDAC4, HDAC5 and 365 E3 ligases available for the indicated 375 cancer cell lines of the Cancer Cell Line Encyclopedia.

**Supplementary Table 2.** Characteristics of CRC patients whose biopsies were used for the TMA.

**Supplementary Table 3.** .bed files of the SEs identified in HCT-116 cells:

CRC1: WT Untreated esiCT

CRC2: WT Untreated esiCT + OXPT

CRC3: WT Untreated esiHDAC4

CRC4: WT Untreated esiHDAC4 + OXPT

CRC5: *FBXW7^-/-^* Untreated esiCT

CRC6: *FBXW7^-/-^* Untreated esiCT + OXPT

CRC7: *FBXW7^-/-^* Untreated esiHDAC4

CRC8: *FBXW7^-/-^* Untreated esiHDAC4 + OXPT

**Supplementary Table 4.** .bed files of the SEs belonging to group1 and 2 and those directly bound by HDAC4.

**Supplementary Table 5.** minimal signature of 116 genes associated to group1 and 2 of SEs.

**Supplementary Table 6.** TCGA sample ID of CRC patients bearing FBXW7 LOF.

**Supplementary Table 7.** List and sequences of primers used for this study.

**Supplementary Table 8.** Raw data for *in vivo* experiments.

**Supplementary Video 1.** Time-lapse video microscopy of PDM-96 expressing pLS-mP-NR4A2-EGFP treated with OXPT 20 µM at time 0.

**Supplementary Video 2.** Time-lapse video microscopy of PDM-96 expressing pLS-mP-NR4A2-EGFP treated with OXPT 20 µM + #11 1 µM at time 0.

**Supplementary Video 3.** Time-lapse video microscopy of PDM-96 expressing pLS-mP-RNF43-EGFP treated with OXPT 20 µM at time 0.

**Supplementary Video 4.** Time-lapse video microscopy of PDM-96 expressing pLS-mP-RNF43-EGFP treated with OXPT 20 µM + #11 1 µM at time 0.

**Supplementary File 1.** Ethical documentation.

**Supplementary Figures**

**
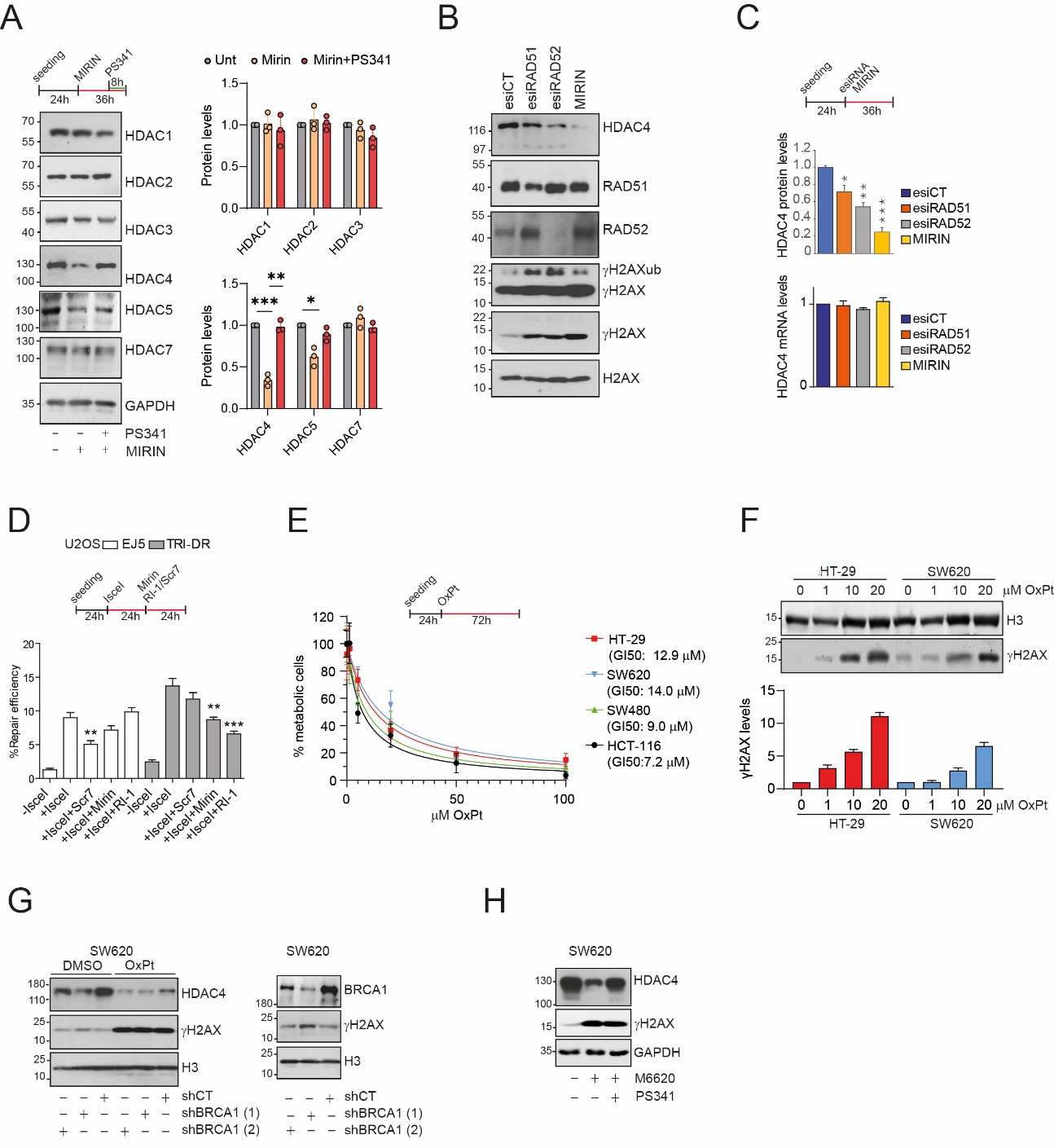
**

**Supplementary Figure 1. Inhibition of homologous recombination (HR) repair leads to a reduction in HDAC4 protein levels across various cellular contexts.** **A.** Immunoblot analysis of the indicated proteins in SK-LMS-1 cells treated as indicated (Mirin 20µM, PS341 1µM). Histogram reporting quantification is provided. **B.** Immunoblot analysis in SK-LMS-1 cells silenced for RAD51 or RAD52 or treated with 20 µM Mirin for 36 h. **C.** Quantification of HDAC4 protein and mRNA levels, in respect to control condition, in cells treated as in Fig. S1B. Data are represented as means ± standard deviation, n=3. Comparisons are relative to esiCT. **D.** % of GFP+ cells which correlates with the efficiency of NHEJ repair in U2OS EJ5-GFP and HR repair in U2OS TriDR-GFP cells after the cleavage with the endonuclease I-SceI. Cells were treated with Scr7 20μM, Mirin 20μM or RI-1 20μM, as indicated. Scr7 significantly affects the repair by NHEJ in EJ5-GFP cells, while Mirin and RI-1 significantly affect homology-directed repair in TriDR-GFP cells. Data are represented as means ± standard deviation, n=3. **E.** GI50 was calculated in the indicated CRC cell lines treated with increasing doses of OXPT and assessed by resazurin assay 72 hours post-treatment. **F.** Immunoblot analysis of the indicated proteins in SW620 or HT-29 cells treated as indicated for 36h. **G.** SW620 cells were stably silenced for BRCA1 (using one control shRNA and two BRCA1-targeting shRNAs) and were treated or not for 30h with 20 μM OXPT. Lysates were prepared and subjected to immunoblotting and probed with the corresponding antibodies shown in the figure. **H.** SW620 cells were treated with 100 nM Berzosertib (M6620) for 36 hours, with the final 8 hours in the presence of 1 μM PS341. Lysates were prepared and subjected to immunoblotting and probed with the corresponding antibodies shown in the figure.


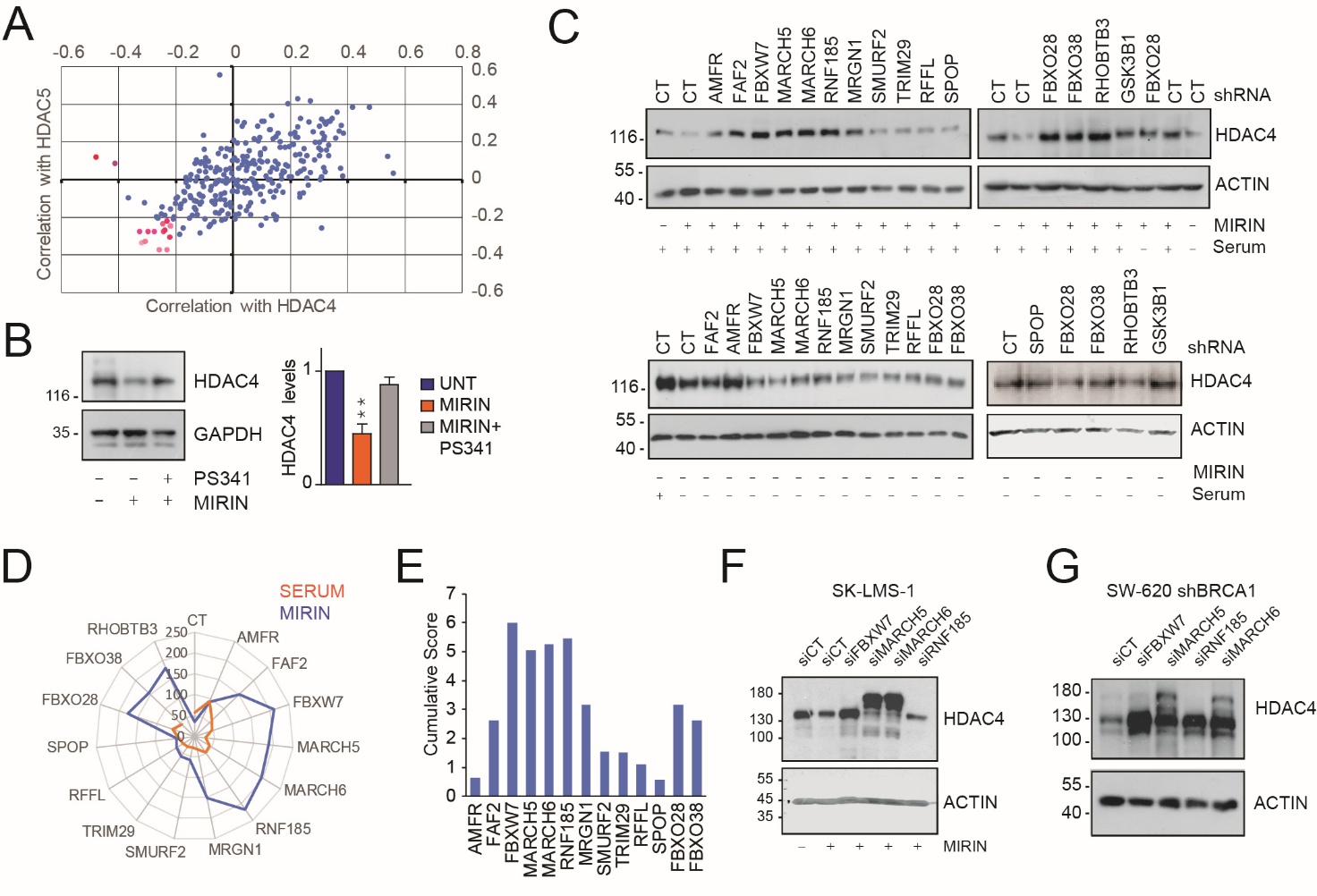


**Supplementary Figure 2. Identification of the E3 ligases involved in HDAC4 degradation through in silico and in vitro screenings.** **A.** In silico screening representing the correlation between HDAC4 and HDAC5 (the two most expressed class IIa HDACs) and 365 E3 ligases available for the indicated 375 cancer cell lines of the Cancer Cell Line Encyclopedia. 14 E3 ligases highlighted in pink were selected for being negatively correlated with HDAC4 and/or HDAC5 protein levels**. B.** Immunoblot analysis and quantification of HDAC4 protein levels in 293T cells treated with Mirin 20μM for 36h and PS341 1μM for the last 8h. **C.** Secondary screening: the expression of the 14 identified E3 ligases was knocked-down with lentiviral shRNAs in 293T cells. Silenced cells were treated with Mirin 20μM for 24h or cultivated in serum starvation conditions (0.2% FBS, 24h, a key determinant of HDAC4 degradation independently from DNA damage), then cells were harvested and the levels of HDAC4 were assessed by immunoblotting. **D.** Radar chart reporting the percentage of residual HDAC4 after Mirin treatment or serum starvation and the knock-down of the reported 14 E3-ligases in respect to shCT. **E.** Cumulative score expressed the ratio between HDAC4 protein levels in Mirin vs Serum starvation; a score above 3 was considered for the next step. **F, G**. Tertiary screening: Immunoblot analysis of HDAC4 in SK-LMS-1 cells silenced for the indicated E3-ligases and treated for 36h with Mirin (F) or in SW620 cells (G) knocked-down for BRCA1 and in which the expression of the indicated E3-ligases was silenced for 48h.


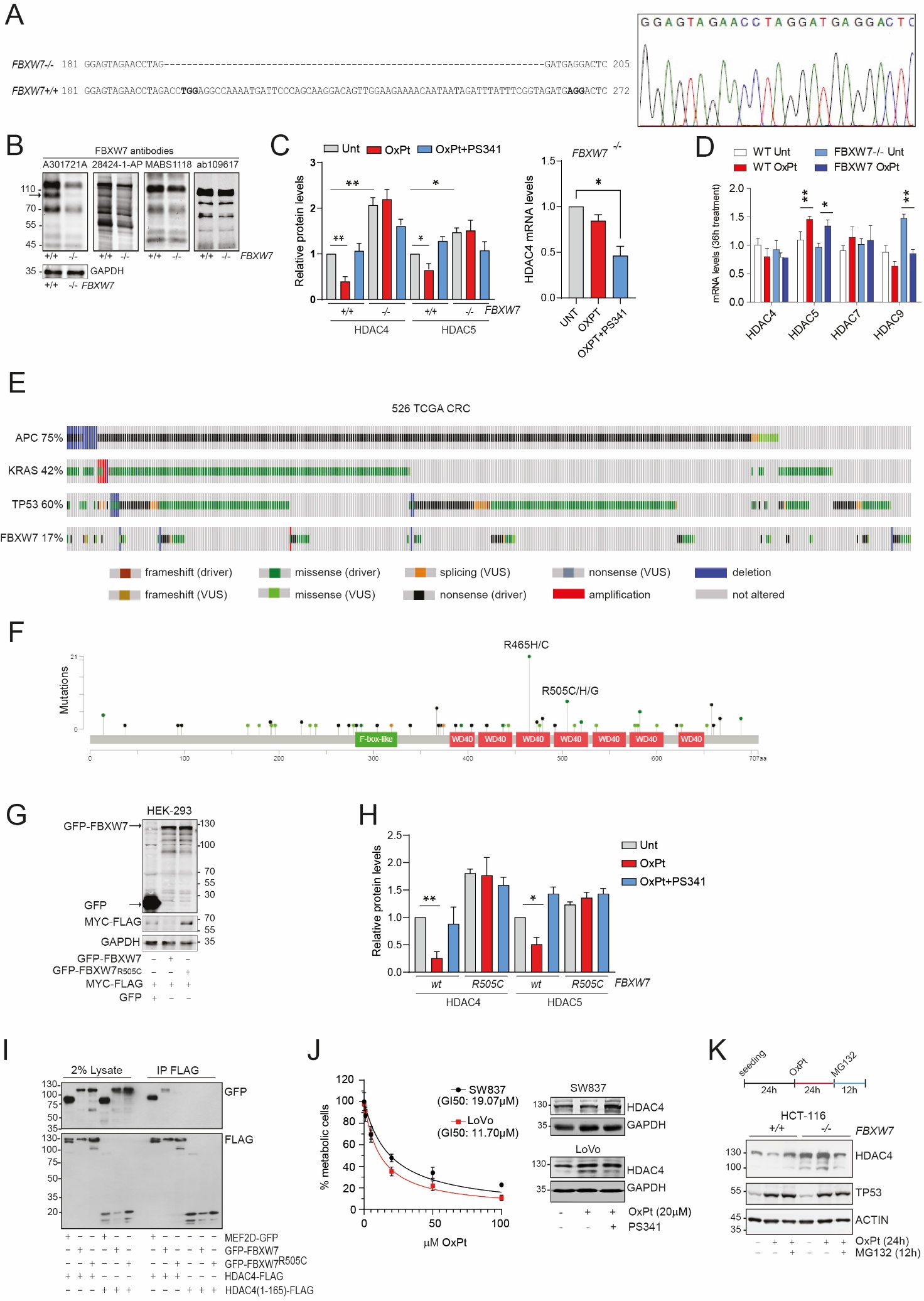


**Supplementary Figure 3. Characterization of FBXW7-/- cells and FBXW7 R505C. A.** Sanger sequencing of FBXW7 WT and KO cells. These latter are characterized by a deletion of 67 nucleotides. **B.** Immunoblot assay to evaluate FBXW7 protein levels by using the indicated commercial antibodies. **C.** Quantification of HDAC4 and HDAC5 protein levels (left) and HDAC4 mRNA levels (right) in the indicated HCT-116 cells treated as in Fig. 2B. n=3. **D.** Quantification of Class IIa HDACs mRNA levels in the indicated HCT-116 cells treated as in Fig. 2B. n=3. **E.** Oncoprint diagram of mutational profile of 526 TCGA CRC samples. **F.** FBXW7 Hot-spot mutations in TCGA CRC samples. **G.** 293 cells were transfected with 2µg of pEGFPC1-FBXW7, FBXW7 R505C or pEGFPC1 and 2µg of pCMV4a-FLAG-MYC. After 48h cells were harvested and subjected to immunoblotting. **H.** Quantification of HDAC4 and HDAC5 protein levels in the indicated SW620 cells treated as in Fig. 2C. n=3. **I.** Co-immunoprecipitation assay in 293 cells expressing HDAC4-FLAG or HDAC4 1-165-FLAG and MEF2D-GFP or GFP-FBXW7 or GFP-FBXW7 R505C. 1µg anti-FLAG antibody was used. FBXW7 but not FBXW7 R505C was co-immunoprecipitated by HDAC4-FLAG. **J.** SW837 and LoVo cells were treated with increasing concentrations of OXPT and GI50 was calculated through resazurin assay at 72h of treatment. Lysates were generated at 36h of treatment and Immunoblot analysis of the indicated proteins was performed (OXPT 20µM). **K.** Immunoblot analysis of the indicated proteins in HCT-116 cells treated as indicated.

**
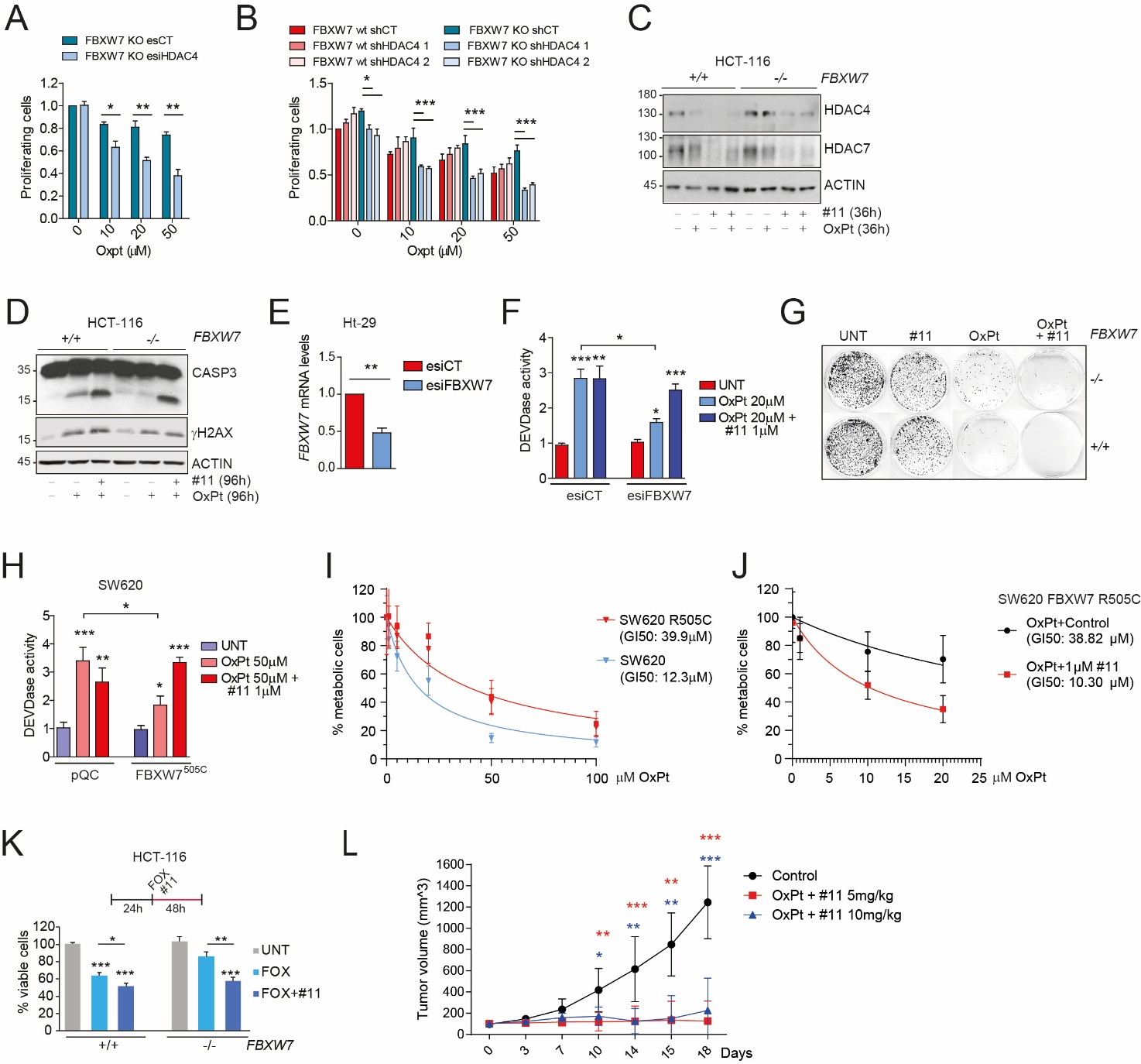
**

**Supplementary Figure 4. HDAC4 forced degradation or silencing increased OXPT cytotoxicity.** **A.** Fraction of proliferating HCT-116 FBXW7^-/-^ cells silenced or not for HDAC4 for 24h and then treated with the indicated concentrations of OXPT for 72h, evaluated by resazurin assay. n=3 **B.** Fraction of proliferating HCT-116 cells WT or FBXW7^-/-^ in which HDAC4 expression was stably knocked-down with the indicated shRNAs and treated with the indicated concentrations of OXPT for 72h, evaluated by resazurin assay. n=5. **C, D.** Immunoblot analysis of the indicated proteins in HCT-116 WT or FBXW7^-/-^ cells treated as indicated for 36h (in C) or 96h (in D). OXPT 50µM, #11 1µM. **E.** mRNA levels of FBXW7 from HT-29 cells transfected with esiCT and esiFBXW7 for 72h. n=3. **F.** DEVDase activity evaluated by Apo-ONE assay in HT-29 cells silenced or not with esiFBXW7 for 72 h and treated with OXPT 10μM or OXPT 10μM + #11 1μM for the last 48 h. Statistics are relative to untreated sample for each condition (esiCT and esiFBXW7). Pairwise comparison is indicated. n=3. **G.** Representative images of the colony formation assay in HCT-116 WT or FBXW7-/- cells, treated as indicate for 24h and kept growing for 7 days. **H.** DEVDase activity evaluated by Apo-ONE assay in SW620 cells overexpressing or not FBXW7 R505C, treated with OXPT 50μM or OXPT 50μM + #11 1μM for 48h. Statistics is relative to untreated sample for each condition (Control, indicated as pQC, and FBXW7 R505C). Pairwise comparison is indicated. n=3. **I, J.** SW620 and SW620 FBXW7^R505C^ cells were treated with increasing concentrations of OXPT and GI50 was calculated through resazurin assay at 72h of treatment. In J, SW620 FBXW7^R505C^ cells were treated with indicated concentrations of OXPT and 1µM #11 were indicated**. K.** Histogram showing the percentage of HCT-116 WT or FBXW7^-/-^ viable cells after 72h of the indicated treatments (FOX: 5-FU 20μM + OXPT 20μM; #11 1μM), evaluated by resazurin assay. n=3. **L.** Tumour volumes were measured in the HCT-116 FBXW7^⁻/⁻^ xenograft mouse model treated twice weekly with either vehicle (n=6), OXPT 5 mg/kg + PROTAC #11 5 mg/kg (n=6), or OXPT 5 mg/kg + PROTAC #11 10 mg/kg (n=6). Statistical significance was calculated at each time point relative to the control group and is indicated by red asterisks (comparison: Control vs. OXPT 5 mg/kg + PROTAC #11 5 mg/kg) or blue asterisks (comparison: Control vs. OXPT 5 mg/kg + PROTAC #11 10 mg/kg). In A, B, E, F, G, H, K data are reported as means ± st.dev. t-test for pairwise comparison, Dunn-multiple comparison test for multiple testing.


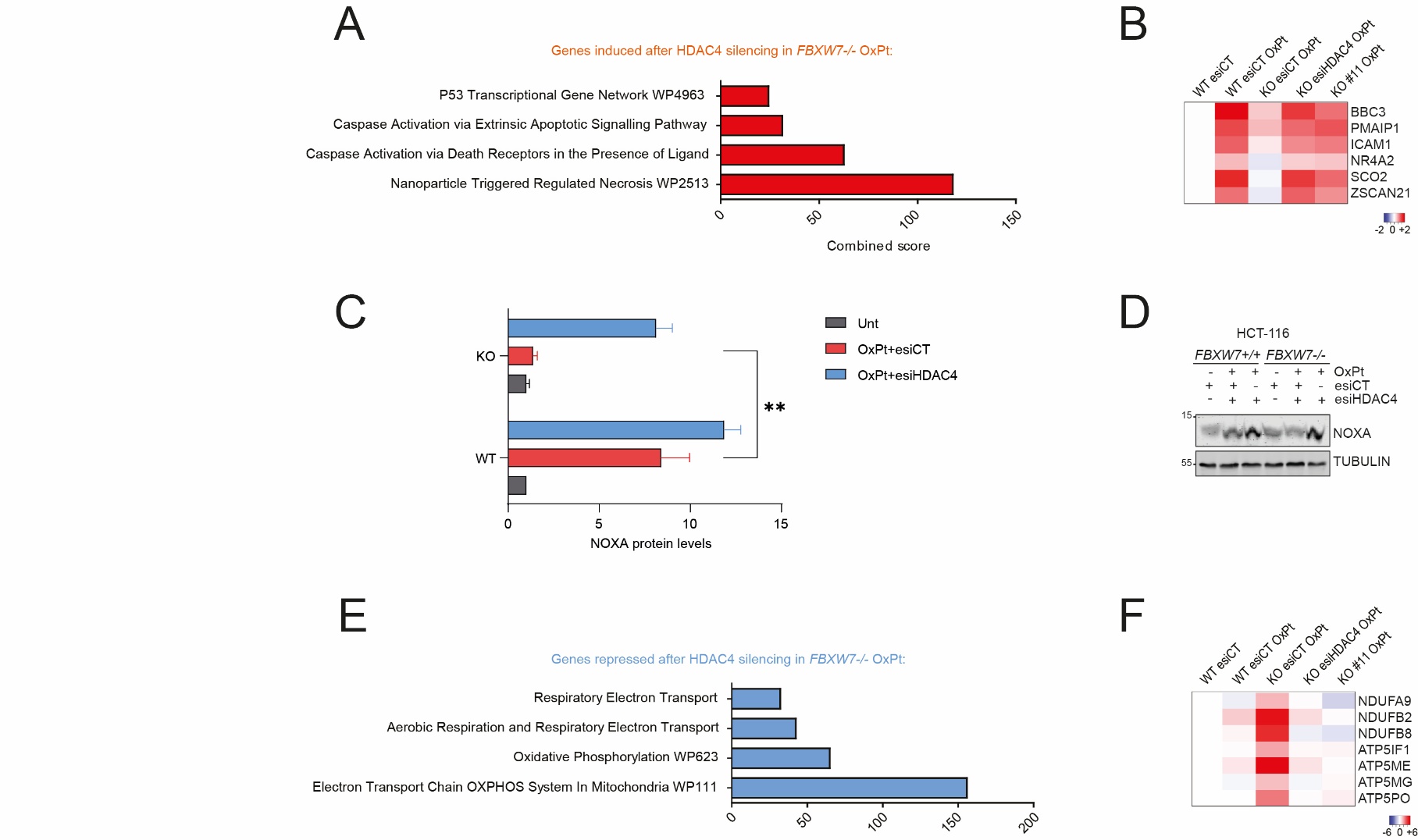


**Supplementary Figure 5. Identification of a signature of genes under the control of HDAC4. A.** Histogram reporting the combined scores of the functional categories found enriched in the genes induced after HDAC4 silencing in FBXW7-/- HCT-116 treated with OXPT. Functional enrichment was computed with Enrichr. **B.** Heatmaps showing the strong dependence on HDAC4 for the transcriptional control of the indicated genes belonging to “Caspase activation” categories. **C, D.** Immunoblot analysis and quantification analysis of NOXA protein levels in the indicated cells treated for 24h with OXPT 50 µM. **E.** Histogram reporting the combined scores of the functional categories found enriched in the genes induced after HDAC4 silencing in FBXW7-/- HCT-116 treated with OXPT. Functional enrichment was computed with Enrichr. **F.** Heatmaps showing the strong dependence on HDAC4 for the transcriptional control of the indicated genes belonging to “OXPHOS” categories.


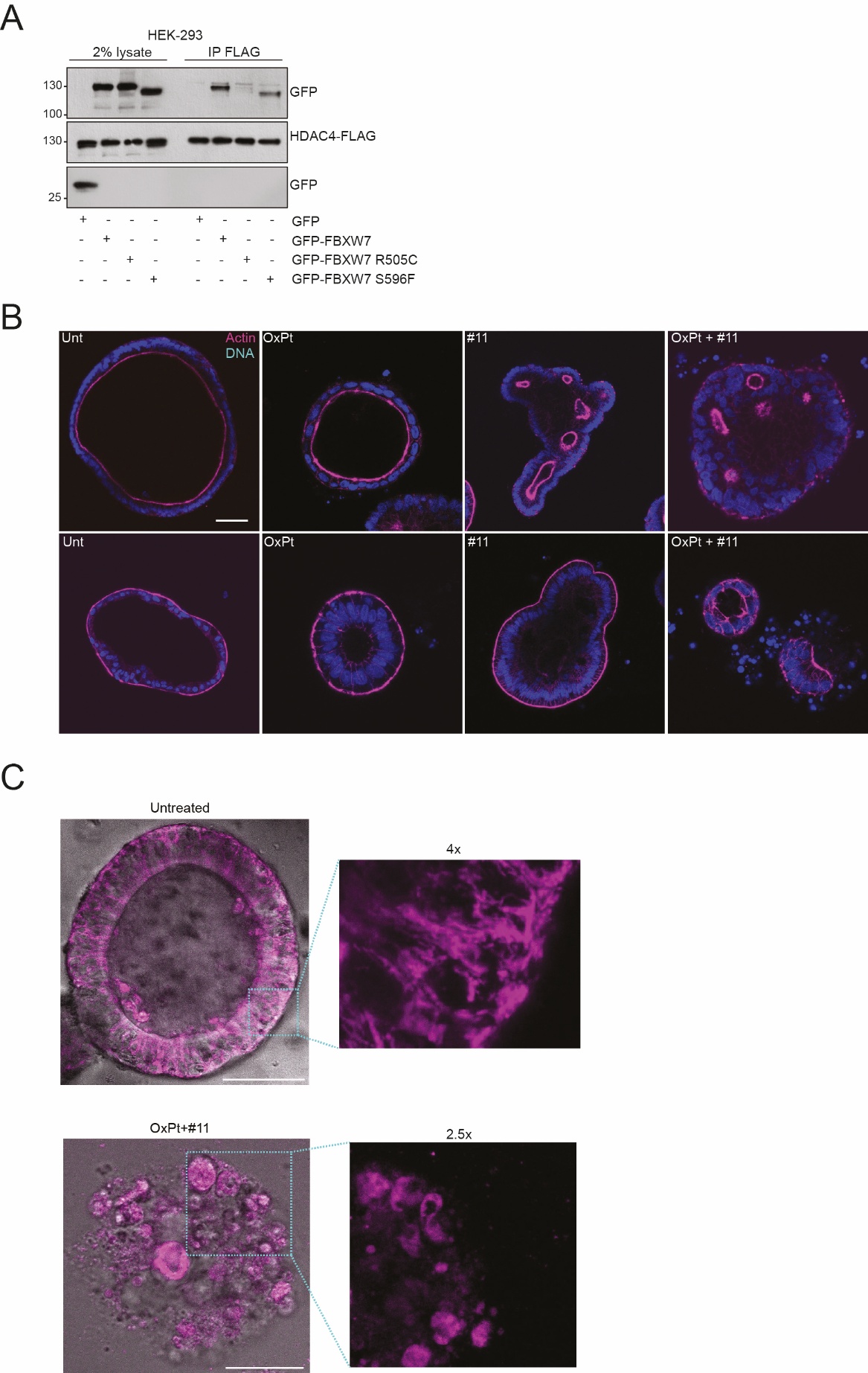


**Supplementary Figure 6. Characterization of PDOs. A.** 293 cells were transfected with GFP, GFP-FBXW7, GFP-FBXW7^R505C^ or GFP-FBXW7^S596F^ and HDAC4-FLAG and treated for 24h with 50µM OXPT and for the last 12h with 2.5µM MG132. FBXW7 and FBXW7^S596F^, but not FBXW7^505C^, were co-immunopurified by anti-FLAG. **B.** Representative confocal images of equatorial sections of PDM-96, treated as indicated and displaying normal polarity (above) or inverted polarity (TSIPs, quantified in Fig. 6B). Scale bar = 50 μm. **C.** PDM-96 PDOs, pre-treated for 96h with OXPT+#11 or left untreated, were loaded for 30’ with TMRM and imaged by confocal live-imaging. Mitochondrial fragmentation and dissipation of Δψm was evident in OXPT+#11 co-treatment. Scale bar = 50 μm.


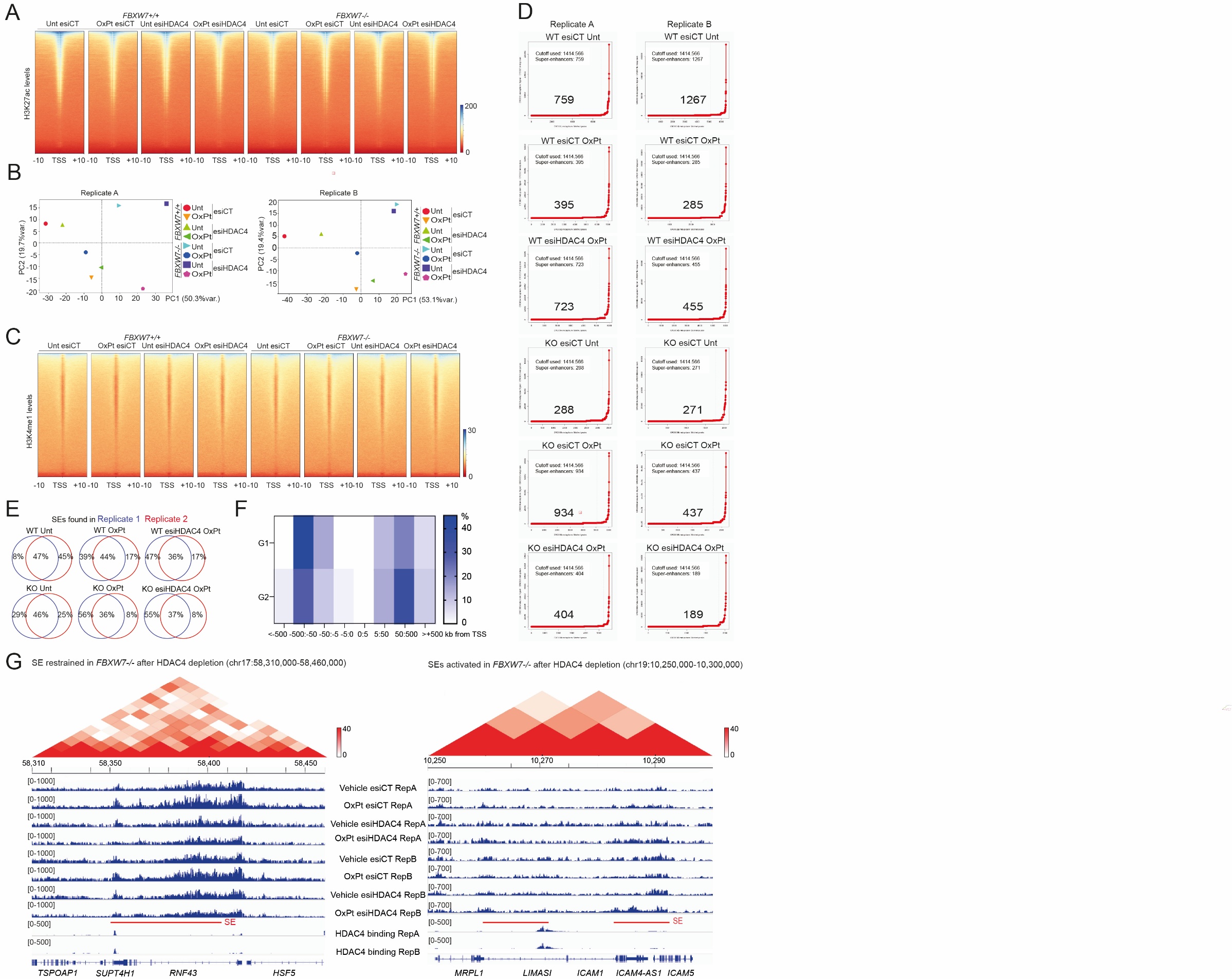


**Supplementary Figure 7. Characterization of the epigenetic response driven by HDAC4. A.** Heatmap of H3K27ac reads distribution within 20kb from the TSS in the indicated samples. **B.** PCA analysis based on H3K27ac peaks of the indicated samples. **C.** Heatmap of H3K4me1 reads distribution within 20kb from the TSS in the indicated samples. **D.** Plots representing the number of SEs identified by ROSE algorithm in the indicated samples, treated as explained in Fig. 7 and in the main text. **E.** Venn diagrams representing the overlap between the SEs identified in biological replicates A and B. **F.** Heatmap representing the distribution of H3K27ac peaks within bona fide SEs belonging to group 1 and 2 in respect to TSS. **G.** ChIP-seq H3K27ac levels in two representative genomic loci containing SEs belonging to G1 (left) and G2 (right) groups. Hi-C data were from HCT-116 cells [81]. HDAC4 peaks falling within SEs were previously identified [51].


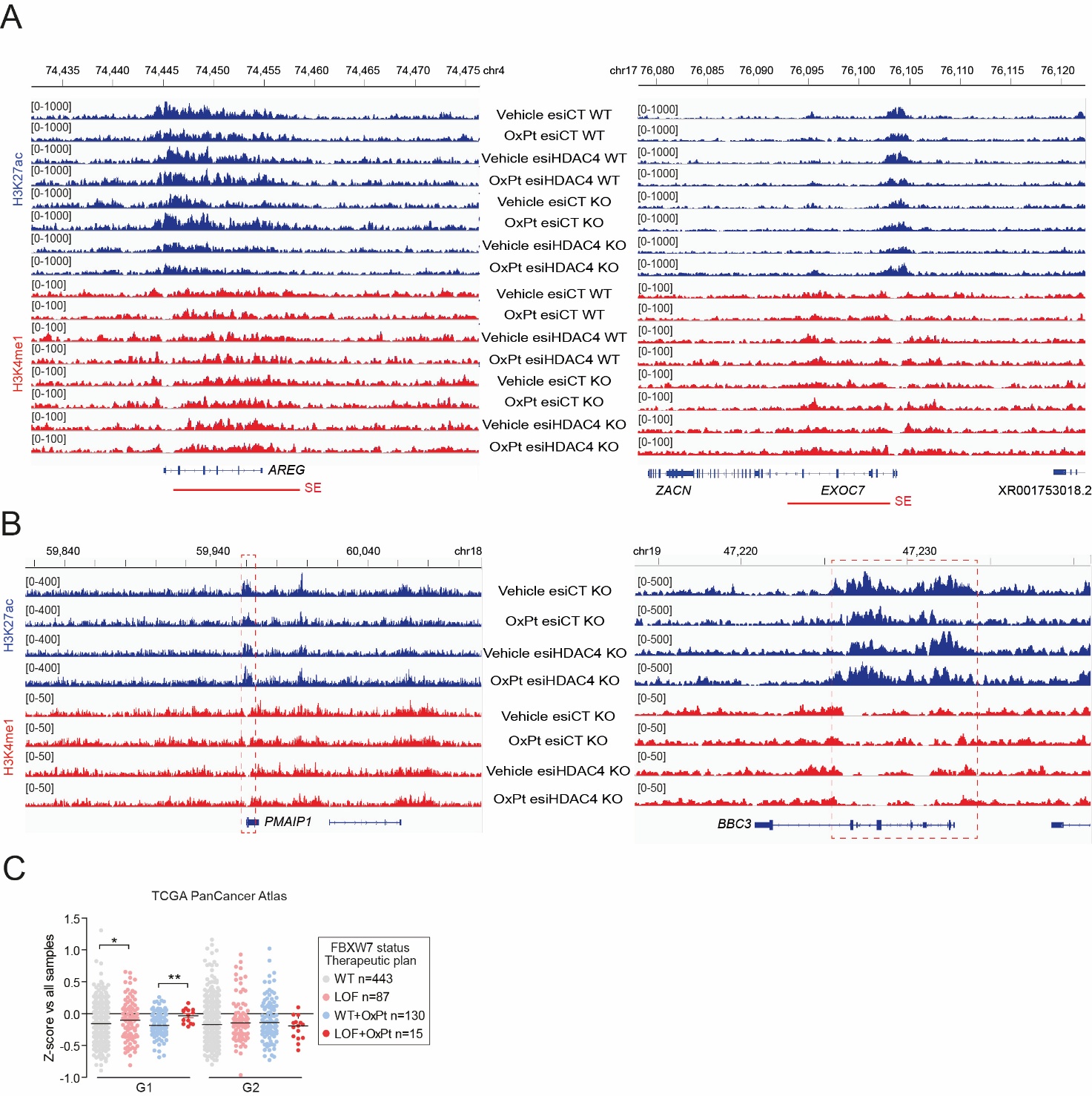


**Supplementary Figure 8. Dissection of the epigenetic response driven by HDAC4. A.** ChIP-seq H3K27ac and H3K4me1 levels in two representative genomic loci containing SEs belonging to G1 (left) and G2 (right) groups. **B.** ChIP-seq H3K27ac and H3K4me1 levels in two genomic loci controlling the expression of PMAIP1 and BBC3. The promoter (PMAIP1) and enhancer (BBC3) regions hyperacetylated after HDAC4 knock-down are highlighted. **C.** Histogram representing the z-scores of the expression levels of the genes associated to G1 and G2 SEs in the indicated TCGA samples. WT and LOF are relative to the status of FBXW7 (WT or LOF=loss-of-function), as reported in Table S6.


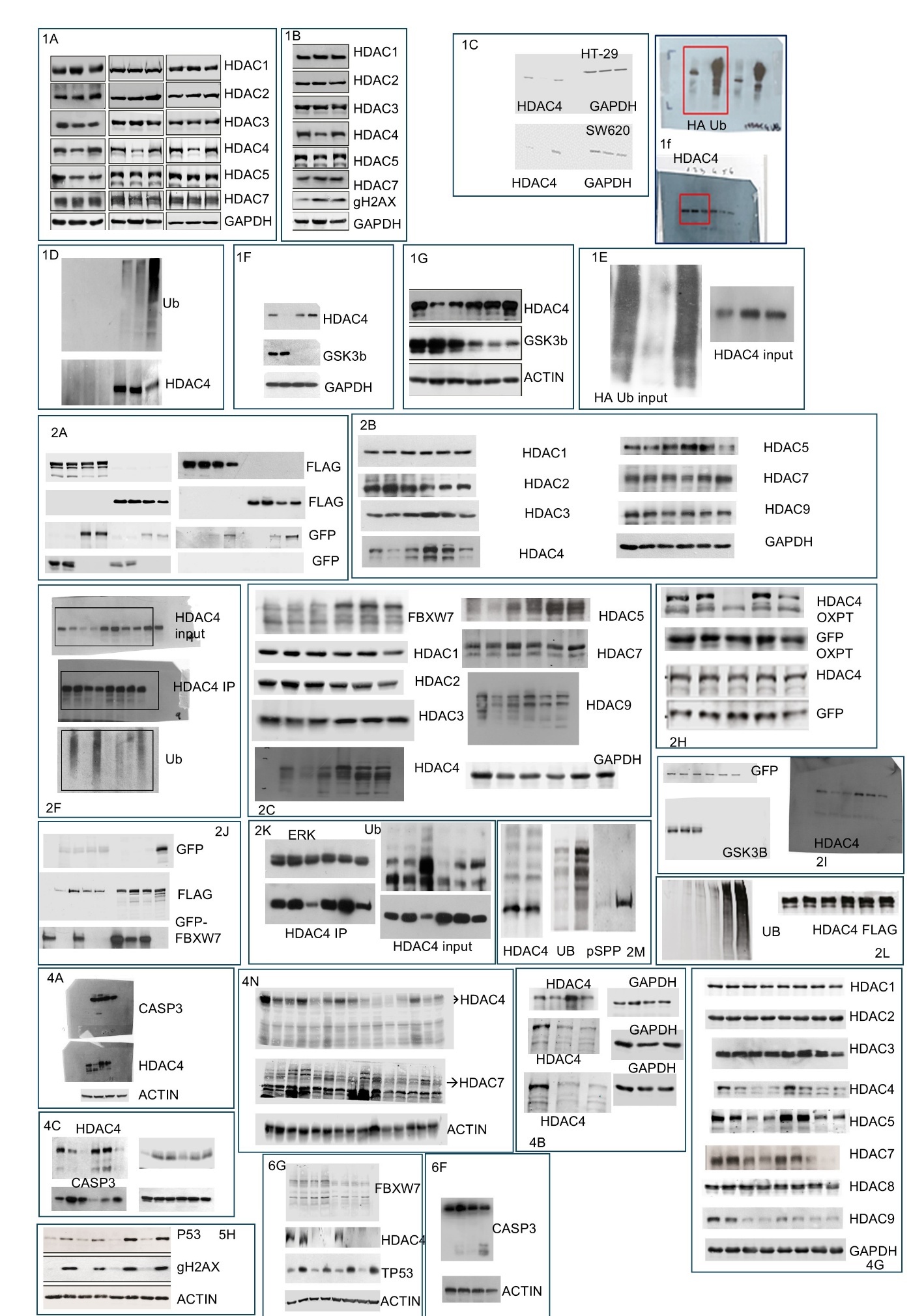


**Supplementary Figure 9.** Original images used for the composition of the immunoblot panels in the main figures.


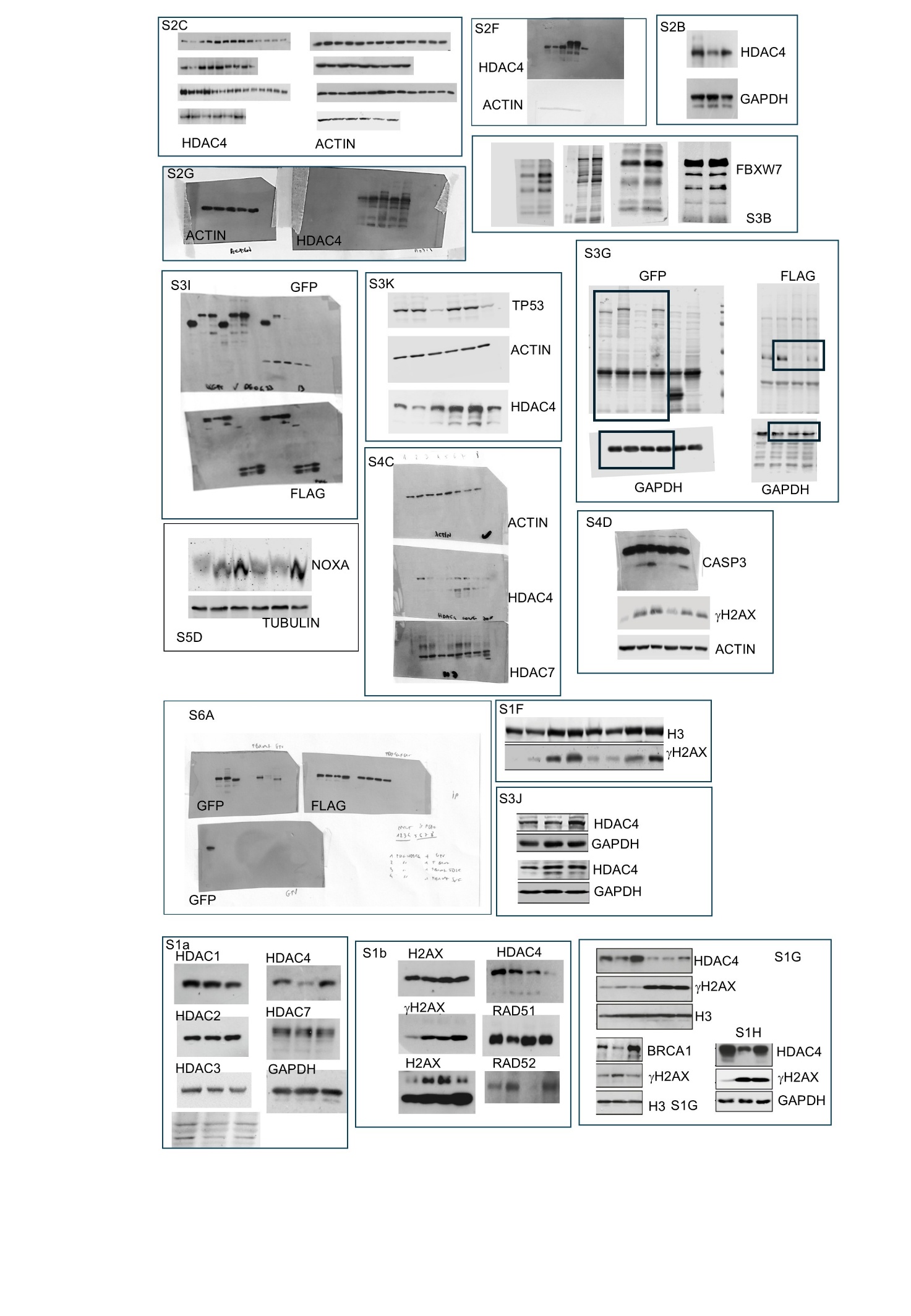


**Supplementary Figure 10.** Original images used for the composition of the immunoblot panels in the supplementary figures.
