# Supplementary material for: Class IIa HDACs forced degradation allows resensitization of oxaliplatin‐resistant FBXW7‐mutated colorectal cancer
Source: Mol Oncol. 2025 Oct 31;20(3):637–67. doi: 10.1002/1878-0261.70152 (PMC13042376; doi:10.1002/1878-0261.70152)
Supplement: Supplementary file 1 — Fig. S1. Inhibition of homologous recombination (HR) repair leads to a reduction in HDAC4 protein levels across various cellular contexts. Fig. S2. Identification of the E3 ligases involved in HDAC4 degradation through in silico and in vitro screenings. Fig. S3. Characterization of FBXW7−/− cells and FBXW7 R505C. Fig. S4. HDAC4 forced degradation or silencing increased OXPT cytotoxicity. Fig. S5. Identification of a signature of genes under the control of HDAC4. Fig. S6. Characterization of PDOs. Fig. S7. Characterization of the epigenetic response driven by HDAC4. Fig. S8. Dissection of the epigenetic response driven by HDAC4. Fig. S9. Original images used for the composition of the immunoblot panels in the main figures. Fig. S10. Original images used for the composition of the immunoblot panels in the supplementary figures. Table S1. Protein expression levels (z‐score) of HDAC4, HDAC5 and 365 E3 ligases available for the indicated 375 cancer cell lines of the Cancer Cell Line Encyclopedia. Table S2. Characteristics of CRC patients whose biopsies were used for the TMA. Table S3. .bed files of the SEs identified in HCT‐116 cells. Table S4. .bed files of the SEs belonging to group 1 and 2 and those directly bound by HDAC4. Table S5. Minimal signature of 116 genes associated to group 1 and 2 of SEs. Table S6. TCGA sample ID of CRC patients bearing FBXW7 LOF. Table S7. List and sequences of primers used for this study. Table S8. Raw data for in vivo experiments. Video S1. Time‐lapse video microscopy of PDM‐96 expressing pLS‐mP‐NR4A2‐EGFP treated with OXPT 20 μm at time 0. Video S2. Time‐lapse video microscopy of PDM‐96 expressing pLS‐mP‐NR4A2‐EGFP treated with OXPT 20 μm + #11 1 μm at time 0. Video S3. Time‐lapse video microscopy of PDM‐96 expressing pLS‐mP‐RNF43‐EGFP treated with OXPT 20 μm at time 0. Video S4. Time‐lapse video microscopy of PDM‐96 expressing pLS‐mP‐RNF43‐EGFP treated with OXPT 20 μm + #11 1 μm at time 0. File S1. Ethical documentation. [file MOL2-20-637-s001.zip › mol270152-sup-0024-DataS2.pdf]

PARERE DELL'ORGANISMO PREPOSTO AL BENESSERE ANIMALE<br>Ai sensi dell'Art. 26 del D.Lgs 26/2014		
**Componenti dell'Organismo preposto al benessere animale**	Responsabile benessere animale:	Dott. Francesco Cardile
	Medico Veterinario designato:	Dott. Luigi Marvasi
	Responsabile scientifico:	Dott.ssa Fabiana Colelli
**Contatti**	Luigi Marvasi: luigi.marvasi@biogem.it, 0825 8818 63<br>Francesco Cardile: francesco.cardile@biogem.it, 0825 8818 40<br>Fabiana Colelli: fabiana.colelli@biogem.it	
**Data di riunione dell'Organismo preposto al benessere animale**	24/01/2025	
**Titolo del progetto di ricerca**	Valutazione dell'efficacia di farmaci antitumorali per la messa a punto di una terapia personalizzata contro il colangiocarcinoma intraepatico (iCCA) e l'epatocarcinoma (HCC), mediante l'utilizzo di modelli murini di patient-derived xenograft (PDX), xenograft ortotopici e sottocutanei.	
**Durata del progetto di ricerca**	24 mesi	
**Obiettivi del progetto di ricerca**	Obiettivo del progetto è quello di individuare il bersaglio molecolare responsabile dello sviluppo di colangiocarcinoma intraepatico ed epatocarcinoma al fine di ottenere una terapia personalizzata.<br>Sulla base di dati riportati in letteratura, si prevede di utilizzare l'inibitore dell'enzima γ-secretasi (Crenigacestat) come inibitore del pathway di Notch1 per validare risultati osservati in vitro e in vivo in precedenti studi sul colangiocarcinoma intraepatico (iCCA). L'efficacia del Crenigacestat è stata osservata in linee over- o costitutivamente esprimenti l'antigene di membrana THY1/CD90, un regolatore chiave nell'interazione cellula-cellula e cellula-matrice, associato ad una prognosi più nefasta. questo farmaco si è dimostrato in grado di ridurre i livelli di espressione della proteina CD90.<br>Tra gli inibitori di epatocarcinoma (HCC), Erlotinib, Gefitinib e Lapatinib sono considerati farmaci d'elezione, mentre Sorafenib e Regorafenib come inibitori della proliferazione. Questi ultimi, seppure con risultati deludenti in termini di overall survival, in esperimenti in vitro hanno mostrato un potenziamento della loro attività farmacologica in presenza di proteine della matrice extracellulare come il proteoglicano 4 (PRG4).<br>A tale scopo si prevede di utilizzare modelli murini di xenograft ottenuti da impianto ortotopico intraepatico o sottocutaneo di cellule o frammenti di tumore di origine umana.	
**Oggetto della presente valutazione**	La presente valutazione ha lo scopo di esaminare la richiesta di utilizzo di 10 topi CD1-nude femmine previste nel progetto di ricerca autorizzato in data 28/03/2023 Codice Autorizzazione 257/2023-PR, per la valutazione dell'effetto della somministrazione dell'antitumorale PROTAC su un modello xenograft di cancro del colon che è solito dare metastasi al fegato..	

**Potenziali benefici del progetto e contributo apportato al progresso scientifico o tecnologico nell'interesse della salute dell'uomo o degli animali**	L'elevato tasso di incidenza di queste due forme di tumori epatici è fondamentale per delineare le possibili ricadute nell'ambito della salute pubblica. La rapidità evolutiva del iCCA e del HCC è tale da non permettere una diagnosi precoce della malattia e molte diagnosi arrivano quando la malattia è già in uno stadio avanzato e non è più possibile intervenire chirurgicamente. Ad oggi l'unica terapia d'elezione contro l'epatocarcinoma è la resezione chirurgica e la sopravvivenza postoperatoria è di soli pochi anni. Inoltre la corretta classificazione diagnostica dal punto di vista molecolare delle diverse variabili di questa malattia permetterebbe di individuare lo specifico pathway coinvolto nella progressione e punterebbe sullo spegnimento o sulla regolazione dello stesso, al fine di individuare nuovi e mirati trial clinici che perfettamente si inseriscono nel quadro di una medicina personalizzata.

**Sintesi delle attività sperimentali (procedure) cui sono sottoposti gli animali**
L'obiettivo dello studio sarà quello di somministrare per via intraperitoneale il farmaco HDAC4 CHDI Degrader 11 (PROTAC) agli animali arruolati e di monitorarne quotidianamente le condizioni cliniche al fine di escludere eventuali effetti tossici e valutare l'efficacia del farmaco sulle cellule HCT116.<br>Gli animali arruolati nello studio saranno sottoposti ad inoculo sottocutaneo di cellule tumorali linee: HCT116 (FBXW7-/-) (10*10^6cellule in 100ul). Lo sviluppo tumorale sarà valutato bisettimanalmente mediante calibro. Circa 7 giorni post-injection verranno avviati i trattamenti farmacologici per 4 settimane.<br>Durante tutto il periodo sperimentale gli animali saranno sottoposti a controlli giornalieri dello stato di salute e a misurazione del peso bisettimanale.
**Livello di sofferenza atteso**
Per la tipologia di studi il livello di sofferenza atteso è **grave**
**Misure adottate per ridurre la sofferenza degli animali**
Le procedure eseguite e/o i trattamenti farmacologici potranno in alcuni casi portare ad effetti avversi sugli animali che pertanto verranno monitorati giornalmente. Tra gli effetti più indicativi per la valutazione dello stato di salute dell'animale, troviamo la perdita di peso corporeo, che se superiore al 20% richiede il sacrificio dell'animale, la perdita di normali atteggiamenti come il grooming, un'accentuata letargia, ridotta mobilità, disidratazione; fino ad arrivare a quelli più gravi come: dispnea, prolasso, atassia, ematuria e, infine, immobilità. Per il controllo di infiammazione e dolore postchirurgico è prevista la somministrazione di analgesici. Per i casi più gravi si ricorre al sacrificio dell'animale.

Parere dell'Organismo preposto al benessere animale sul progetto di ricerca

- Considerato che il cancro del colon-retto (CRC) è uno dei tumori più diffusi al mondo, con una stima di 1,4 milioni di nuovi casi diagnosticati ogni anno, ed è descritto come una malattia "silenziosa", in quanto pochi pazienti con CRC mostrano sintomi clinici fino alle fasi avanzate della malattia, ovvero, quando il cancro ha metastatizzato ad altri organi del corpo, lo studio acquisisce una notevole valenza tecnico-scientifica;

- considerata la corretta applicazione delle 3Rs e la congruità a quanto richiesto dal decreto legislativo vigente;

- considerato che la valutazione degli effetti negativi si basa sugli endpoint umanitari e sul loro punteggio

- considerata la corretta applicazione dei protocolli di anestesia e analgesia quando necessaria;

- considerato che non esistono per questa tipologia di studi delle alternative *in vitro* in sostituzione dei modelli animali in vivo;

In virtù delle considerazioni sopra elencate si rilascia un parere favorevole.

**Esito della valutazione OPBA:**	**FAVOREVOLE**

Il Responsabile scientifico

Fabiano Cotelli

……………………………………………..

Il Veterinario Designato

Luigi Manzoni

……………………………………………..

Il Responsabile del Benessere animale

Francesco Cerolile

……………………………………………..

UNIVERSITA' DEGLI STUDI DI UDINE
ORGANISMO PREPOSTO AL BENESSERE DEGLI ANIMALI

Prot. N. 03/2025
Organismo Preposto al Benessere degli Animali

Gent.ma dott. Cristina Degrassi
MTTlab srl
via Gallina, 5
34122, Trieste
Email. degrassi@mttlab.eu

Alla c.a. del prof. Leonardo Alberto SECHI
Direttore del Dipartimento di Medicina (DMED) dell'Università degli Studi di Udine
Email: leonardo.sechi@uniud.it

Udine, lì 18 aprile 2025

Oggetto: Studio sperimentale – progetto di ricerca "**Valutazione dell'effetto anti-neoplastico di composti PROTAC per indurre la degradazione forzata di target terapeutici in xenotrapianti di cellule tumorali e valutazione dello stato metabolico in presenza di regolatori catabolici dell'arginina**" – Promotore: Dipartimento di Area Medica (DMED) dell'Università degli Studi di Udine – Sperimentatore Responsabile: dott. Cristina Degrassi.

Invio parere dell'O.P.B.A.

In riferimento allo studio in oggetto, si informa che l'Organismo Preposto al Benessere degli Animali, riunitosi in data 18 aprile 2025, ha valutato la documentazione trasmessa ed ha espresso le determinazioni di cui all'allegato.

Si rammenta che la presente lettera non costituisce autorizzazione all'avvio dello studio, presso lo stabilimento dell'Università degli Studi di Udine (via Faedis 46, 48, 50 33100 Udine (UD)), e che data autorizzazione sarà formalizzata con l'invio presso il Ministero della Salute.

Nel trasmettere copia della valutazione in oggetto si coglie l'occasione per porgere i migliori saluti.

Il presidente dell'O.P.B.A
Prof. Giuseppe Stradaioli

Giuseppe Stradaioli

UNIVERSITA' DEGLI STUDI DI UDINE
ORGANISMO PREPOSTO AL BENESSERE DEGLI ANIMALI

Verbale – Riunione del 18 aprile 2025

Parere N. 03/2025

Giorno 18 aprile 2025, alle ore 12:20 si è svolta la riunione dell'Organismo Preposto al Benessere egli Animali (O.P.B.A.) per via telematica.

Componenti del O.P.B.A.

Componente	Presente	Assente	A.G.
STRADAIOLI prof. Giuseppe, *Presidente*[1]	X		
PUCILLO Prof. Carlo Ennio Michele	X		
LIPPE prof. Giovanna [2]	X		
COLITTI prof. Monica	X		
TELL prof. Gianluca	X		
PESARO dott. Stefano	X		
FABRIS dott. Andrea	X		
Volpatti dott. Donatella *Segretario Verbalizzante*	X		

[1] Veterinario designato (art. 25 comma 2 D.Lgs. n. 26/2014);

[2] Responsabile del benessere animale dello Stabilimento sito in via Faedis 46, 48, 50 33100 Udine.

I sopraindicati Componenti dell'O.P.B.A. dichiarano di astenersi dal pronunciarsi sulle sperimentazioni per le quali sussiste conflitto di interesse di tipo diretto o indiretto.
L'O.P.B.A. è istituito in conformità alle disposizioni del Ministero della Salute tramite decreto Rettorale n. 742 del 06.10.2020.

1 – Studio sperimentale di ricerca di base.

2 – Studio sperimentale - progetto di ricerca "**Valutazione dell'effetto anti-neoplastico di composti PROTAC per indurre la degradazione forzata di target terapeutici in xenotrapianti di cellule tumorali e valutazione dello stato metabolico in presenza di regolatori catabolici dell'arginina**" – Promotore: Dipartimento di Medicina (DMED) dell'Università degli Studi di Udine – Sperimentatore Responsabile: dott. Cristina Degrassi. L'organismo dopo attenta lettura della documentazione relativa al progetto ha constatato che ha una buona rilevanza scientifica supportata da una letteratura adeguata.

Il progetto risponde in modo soddisfacente all'applicazione del principio della sostituzione, della riduzione e del perfezionamento e che l'uso degli animali e il numero di soggetti impiegati è adeguatamente motivato anche sulla scorta di una documentazione bibliografica appropriata e aggiornata.

La formazione e la congruità dei ruoli professionali del personale utilizzatore coinvolto nel progetto è di ottimo livello.
Le procedure attuate per il benessere animale da quelle messe in atto per la creazione di un ambiente che permetta la stabulazione dei topi a quelle di sorveglianza dei parametri di benessere, alle procedure sperimentali e agli approcci per la riduzione del dolore fino alla soppressione dell'animale sono atte a garantire il minimo o l'assenza di stress agli animali stabulati. Infatti, la stabulazione avverrà in locali che assicurano temperatura di 22°C ± 2°C con umidità relativa 55% ± 5 %, illuminati o oscurati per garantire un corretto ciclo di luce/buio nell'arco delle 24h. La stabulazione di animali avverrà in ambienti e gabbie IVC, rispettando i parametri relativi al rapporto superficie della gabbia/peso dell'animale come richiesto dalla normativa (Allegato III sezioni A, B e tabella 1.1 del D.Lgs 26 del 24 marzo 2014). Gli animali verranno alloggiati su lettiere che verranno cambiate una volta a settimana, alimentati ad libitum e abbeverati tramite erogatore a bottiglia nella quale l'acqua verrà cambiata una volta a settimana. Inoltre, gli animali saranno costantemente monitorati per parametri di benessere quali lo stato del mantello, alterazioni delle funzioni organiche, dimagrimento o lo stato di stress seguendo una Tabella a punteggi standardizzata per la valutazione della sofferenza negli animali da laboratorio, modificata da Wolfensohn e Lloyd (2003). Qualsiasi aspetto anomalo o patologico notato verrà segnalato dallo sperimentatore al responsabile dello Stabulario che provvederà ad informare il Medico Veterinario responsabile per l'allevamento affinché attui le procedure per salvaguardare il benessere e tutelare la salute degli animali stabulati. Le procedure che richiedono l'immobilizzazione degli animali saranno condotte previo addestramento al fine di ridurre ogni forma di stress connessa alle procedure stesse.

Per ridurre al minimo la sofferenza degli animali la loro soppressione ove necessaria avverrà mediante dislocazione cervicale, che risulta essere la meno dolorosa e più rapida per l'animale (allegato IV D.Lgs 26 del 24 marzo 2014).

Infine, valutata la conformità della documentazione con quella richiesta dal D.Lgs 26 del 24 marzo 2014 il Comitato esprime all'unanimità parere favorevole sul progetto presentato dal dott. Cristina Degrassi. Il progetto con la relativa documentazione dovrà essere inviato al Ministero della Salute per via telematica.

Segretario Verbalizzante
Dott. Donatella Volpatti

Il presidente dell' O.B.P.A
Prof. Giuseppe Stradaioli

1. UTILIZZATORE ***(persona fisica o giuridica ai sensi della lett. f) dell'articolo 3)***

Magnifico Rettore dell'Università degli Studi di Udine, sede legale in Via Palladio 8, 33100 Udine

(UD).

2. TITOLO DEL PROGETTO DI RICERCA

Valutazione dell'effetto anti-neoplastico di composti PROTAC per indurre la degradazione forzata di target terapeutici in xenotrapianti di cellule tumorali e valutazione dello stato metabolico in presenza di regolatori catabolici dell'arginina

3. PAROLE CHIAVE *(massimo 5 parole)*

3.1 Parole chiave: carcinoma colorettale (CRC)

3.2 Parole chiave: adenocarcinoma duttale del pancreas (PDAC)

3.3 Parole chiave: ADI-PEG20

3.4 Parole chiave: KRAS

3.5 Parole chiave: oxaliplatino

4. RESPONSABILE DEL PROGETTO DI RICERCA *(ai sensi della lett. g) dell'articolo 3)*

Cristina Degrassi
Dipartimento o Struttura di afferenza: MTTlab srl
Indirizzo: via Gallina, 5, 34122, Trieste
Recapiti telefono: 327 4080298 *e-mail:* degrassi@mttlab.eu

5. RESPONSABILE DELL'ESECUZIONE DEGLI ESPERIMENTI

Cristina Degrassi
Dipartimento o Struttura di afferenza: MTTlab srl
Indirizzo: via Gallina, 5, 34122, Trieste
Recapiti telefono: 327 4080298 *e-mail:* degrassi@mttlab.eu

6. STABILIMENTO UTILIZZATORE

Stabulario dell'Università di Udine.

Denominazione: Stabulario Dipartimento di Medicina (DMED), centro di Ricerche Cliniche e Traslazionali
Ubicazione: via Faedis 46, 48, 50 33100 Udine (UD).
Estremi autorizzazione decreto ministeriale: decreto n. 07/2024-UT rilasciato in data 08/07/2024.

7. RESPONSABILE DEL BENESSERE ANIMALE *(ai sensi della lett. h) dell'articolo 3)*

Stabulario dell'Università di Udine.

Prof.ssa Giovanna Lippe
Dipartimento o Struttura di afferenza: DMED, Università di Udine.
Indirizzo: Piazzale Massimiliano Kolbe 4, 33100 Udine (UD).
Recapiti telefono: 0432558139 *e-mail:* giovanna.lippe@uniud.it

8. MEDICO VETERINARIO DESIGNATO *(art. 24)*

Stabulario dell'Università di Udine.
prof. Giuseppe Stradaioli
Indirizzo: Dipartimento di Scienze AgroAlimentari, Ambientali ed Animali Via delle Scienze 206, 33100 Udine (UD).
Recapiti telefono: 0432 558580 *e-mail:* giuseppe.stradaioli@uniud.it

9. ELENCO E COMPETENZA DEL PERSONALE CHE PARTECIPA AL PROGETTO DI RICERCA *(ai sensi del comma 2 dell'articolo 23, allegato V)*

Nominativo	*Titolo di Studio o Qualifica*	*Ruolo nello staff*	*Formazione*
Degrassi Cristina	Laurea in Scienze Biologiche	**Funzione b** - Responsabile del Progetto<br><br>**Funzione a** - Esecutore delle procedure sugli animali	*In disciplina transitoria: autorizzazione ministeriale n°546/2019-PR*<br><br>*In disciplina transitoria: autorizzazione ministeriale n°918/2018-PR*
Calvillo Laura	Laurea e PhD	**Funzione a** - Esecutore delle procedure sugli animali	*In disciplina transitoria: autorizzazione ministeriale n°55/2019-PR*
Gianfranco Moretti		**Funzione c** – Cura degli animali	LEGISLAZIONE NAZIONALE ED ETICA LIVELLO 1, MODULI 1 E 2, DM 5 AGOSTO 2021 - 1^ Edizione IZSLER FAD 21/03/2024

		ETICA E CONCEZIONE DEI PROGETTI, MODULI 9, 10, 11, DM 5 AGOSTO 2021 - Edizione Unica IZSLER FAD 20/08/2024 BIOLOGIA E GESTIONE DEGLI ANIMALI DA LABORATORIO, MODULI 3.1, 4, 5, 6.1, 7. DM 5 AGOSTO 2021 RODITORI E LAGOMORFI - 1^ Edizione IZSLER FAD 20/08/2024<br><br>Workshop pratico sui Moduli 3.2, 6.2 e 8 per la formazione specifica per il personale coinvolto nella sperimentazione animale per fini scientifici. Fondazione Guido Bernardini 26/09/2024

10. SI PREVEDE DI UTILIZZARE IL SEGUENTE NUMERO COMPLESSIVO DI ANIMALI *(specificare se animali geneticamente modificati)*

Specie animale *Mus Musculus* n.: 260

11. SPECIFICARE SE ANIMALI GENETICAMENTE MODIFICATI ED EVENTUALI / NUMERI PARZIALI RIFERITI A DIVERSE SPECIE E/O CEPPI ANIMALI

Specie	Ceppo	Numero/ anno
Mus musculus	Nude mice	260

12. GLI ANIMALI SARANNO STABULATI NEI LOCALI

Stabulario dell'Università di Udine.
Denominazione: Stabulario Dipartimento di Area Medicina (DMED), centro di Ricerche Cliniche e Traslazionali
Ubicazione: via Faedis 46, 48, 50 33100 Udine (UD).
Estremi autorizzazione decreto ministeriale: decreto n. 07/2024-UT rilasciato in data 08/07/2024.

13. GLI ANIMALI SARANNO UTILIZZATI NEI LOCALI
Stabulario dell'Università di Udine.

Denominazione: Stabulario Dipartimento di Medicina (DMED), centro di Ricerche Cliniche e Traslazionali
Ubicazione: via Faedis 46, 48, 50 33100 Udine (UD).
Estremi autorizzazione decreto ministeriale: decreto n. 07/2024-UT rilasciato in data 08/07/2024.

14. PROVENIENZA DEGLI ANIMALI

☐ *Allevamento interno*

☐ *Allevatore*

Dati della ditta o altro che alleva gli animali

■ *Fornitore*

Dati della ditta o altro che fornisce gli animali

Envigo, Zona Industriale Azzida 57, 33049 San Pietro al Natisone (UD).

Charles River Laboratories Italia via Indipendenza 11, 23885 Calco (Lecco)

15. DURATA DEL PROGETTO DI RICERCA (massimo 60 mesi)

48 mesi

16. OBIETTIVI DEL PROGETTO DI RICERCA

Il presente progetto di ricerca prevede l’ottenimento di Xenotrapianto di cellule HCT-116 FBXW7 mutate, così come di altre cellule di carcinoma colorettale (CRC) quali SW480 e SW620. Le cellule verranno ingegnerizzazione con la tecnologia CRISPR/Cas9 per esprimere o meno geni che regolano il catabolismo dell’arginina come KRAS, ARG2, CKB, PADI1, PADI3. Le cellule verranno amplificate e iniettate nel sottocute di topi nudi atimici al fine di ottenere la crescita tumorale. Lo scopo sarà quello di confrontare il trattamento dei tumori con oxaliplatino, FOLOX (combinazione di oxaliplatano e 5-fluorouracile), gemcitabina e composti PROTAC #11 (HDAC4 CHDI Degrader 11, un degradatore potente e selettivo per HDAC4) e LC-2 (degradatore di KRAS G12C endogeno) ed infine ADI-PEG. Verranno misurati i volumi dei tumori durante la crescita tumorale ed il trattamento, infine i tumori verranno espiantati e sarà analizzato il trascrittoma ed il proteoma ex vivo. Verrà inoltre eseguita una valutazione metabolomica.

I modelli murini verranno utilizzati per complementare i risultati ottenuti in colture cellulare 2D e 3D. I trattamenti farmacologici previsti rientrano nell'ambito della routine traslazionale dell'oncologia molecolare.

17. Razionale dello studio

1. **Stato delle conoscenze** *(Giustificare lo studio con adeguati riferimenti bibliografici)*

Il cancro del colon-retto (CRC) è uno dei tumori più comuni e la terapia adiuvante che include l'oxaliplatino (OxPt) è considerata lo standard per il CRC avanzato (1,2). Il 17% dei pazienti con CRC reca mutazioni con perdita di funzione (LOF) in FBXW7, un gene oncosoppressore che codifica per un'E3 ligasi della famiglia SCF. FBXW7 LOF induce resistenza ai chemioterapici come OxPt, mentre il trattamento con Vorinostat, un inibitore aspecifico delle HDAC, sensibilizza le cellule tumorali mutate per FBXW7 alla chemioterapia (3-5).

Recentemente è stato identificato FBXW7 come l'E3 ligasi di HDAC4, un HDAC di classe IIa che è stato scoperto essere coinvolto nella riparazione delle rotture a doppio filamento (DSBs) per ricombinazione omologa (HR) (6; *NAR in revisione*). È stato visto che l'inibizione di HDAC4 ripristina la sensibilità a OxPt nelle cellule CRC mutate per FBXW7. Pertanto, l'ipotesi è che le cellule tumorali del colon mutate per FBXW7 abbiano livelli aumentati di HDAC4 per ripristinare il riparo per HR. Inoltre, in complesso con HDAC3, HDAC4 controlla l'acetilazione di H3K27 e regola la formazione di super-enhancer (SE), che possono essere utilizzati per prevedere la sensibilità e la resistenza a OxPt (*Tolotto et al., in preparazione*).

Le chimere per la proteolisi mirata (proteolysis-tatgeting chimeras - PROTACs) sono una classe di molecole degradatori di proteine eterobifunzionali che eliminano le proteine target sfruttando il complesso ubiquitina-proteasoma tramite l'azione delle ubiquitin-protein ligasi E3 e i loro interratori attraendo la proteina target stessa verso la ligasi E3 ed indirizzandola alla via proteolitica proteasomiale 26S (**Figura 1**) (7).

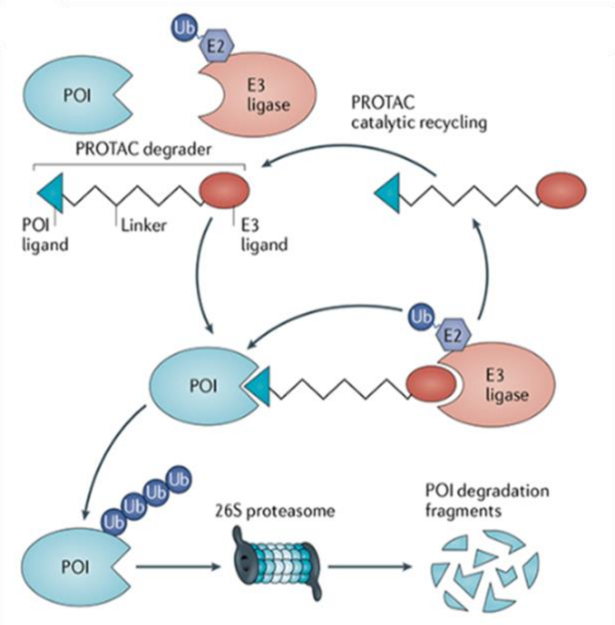

Figura 1: Schema generale di degradatori PROTAC. Proteina di interesse (protein of interest - POI) degradata tramite il sistema unibiquitina proteasona mediata da PROTAC il quale consiste di un ligando per la POI connesso ad un E3 ligasi tramite un connettore (linker). (7)

Il composto #11 è un composto PROTAC recentemente sintetizzato ed ottenuto in collaborazione con un gruppo di ricerca americano. E' già stato caratterizzato in vitro (8) e la sua seeding molecule è stata utilizzata in vivo senza dimostrare tossicità fino a 15mg/kg (9). In *Patient's Derived Organoids* (PDOs) di carcinoma colorettale FBXW7, il trattamento combinato con il composto #11 e l'oxaliplatino determina citotossicità e ri-sensibilizza le cellule tumorali refrattarie al trattamento al OxPt (**Figura 2**).

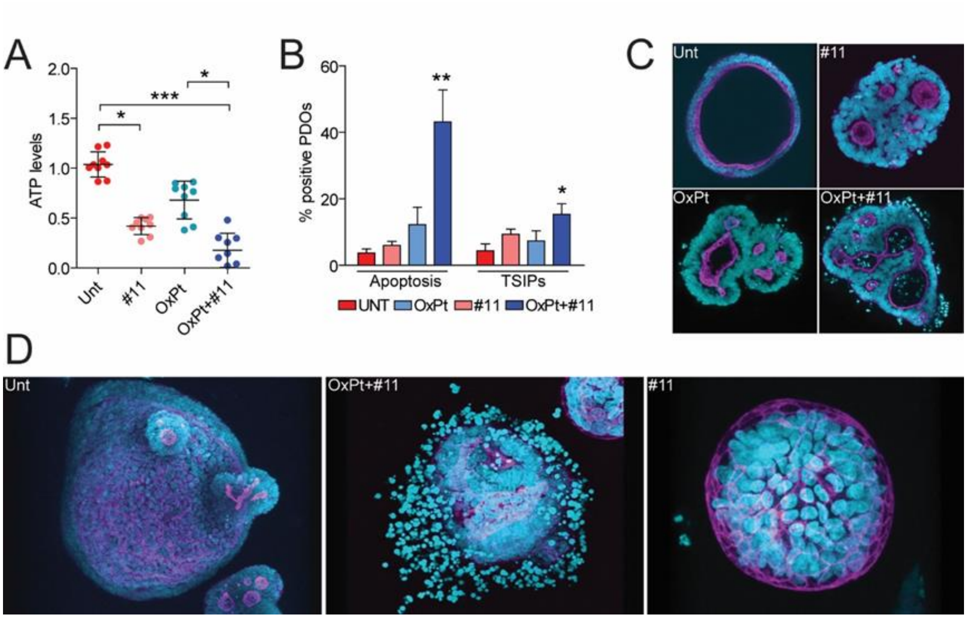

Figura 2: PROTAC #11 ri-sensibilizza i PDO FBXW7 mutati ai sali di platino. A. Livelli di ATP negli organoidi di CRC FBXW7 mutati trattati per 96h con 50uM OxPt e 1uM #11. **B.** % of PDOs displaying apoptotic nuclei or TSIPs (inversita morfologia). **C-D.** Immagini confocali 2D (**C**) e 3D (**D**) di un PDO FBXW7 mutato trattato come indicato. In Blu sono colorati i nuclei (DAPI) in magenta il citoscheletro di actina (falloidina).

In **Figura 3** si riportano le strutture dei composti PROTAC che verranno utilizzati in questo studio. Per le formule di OxPt, FOI, ADI-PEG20 e TDRL-551 si rimanda alla letteratura.

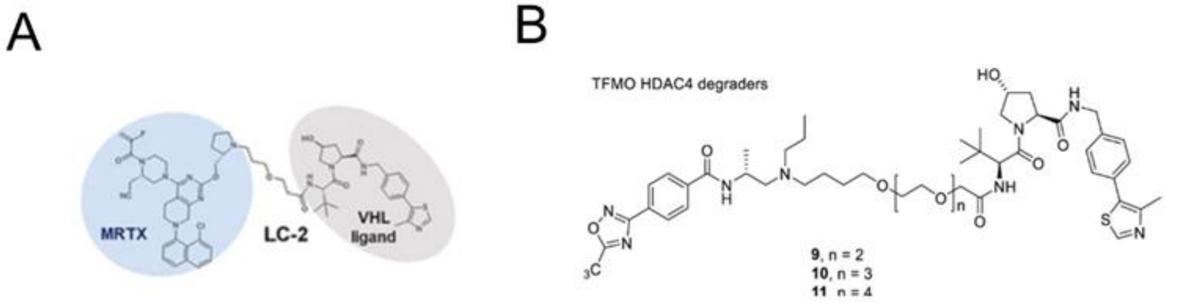

Figura 3: **A.** Formula strutturale di LC-2. **B.** Struttura base dei composti PROTAC da cui deriva il composto #11 con ripetizione modulare n=4 dello scheletro CH3-CH3-O.

2. **Originalità e/o interesse dello studio** *(valore scientifico)*

La ri-sensibilizzazione al platino dei carcinomi CRC FBXW7 mutati costituirebbe un importante progresso qualora si confermasse la base epigenetica di tale refrattarietà. L'esecuzione di tale esperimento *in vivo* permetterebbe di confermare le evidenze scientifiche raccolte *ex-vivo* e in modelli 3D di PDO (organoidi derivati da pazienti) e non ancora oggetto di pubblicazione. Si creerebbe inoltre un atlante epigenetico con cui confrontare i risultati scientifici ottenuti in altri modelli di sensibilità/refrattarietà alle terapie.

La valutazione della riprogrammazione metabolica e della crescita in vivo di cellule tumorali ingegnerizzate per controllare il metabolismo dell'arginina (silenziamento stabile di ASS1, PADI1, PADI3, CKB) permetterà di valutarne un'eventuale efficacia come bersagli terapeutici per il trattamento di PDAC e CRC. Permetterà inoltre di valutare l'efficacia di terapie sostitutive o integrative alla degradazione di KRAS. Infine ci si propone di valutare l'efficacia dei composti

PROTAC (#11 degradatore di HDAC4 e LC-2 degradatore di KRAS G12C) nella terapia anti-neoplastica.

3. **Eventuali ricadute nell'ambito della salute pubblica umana e/o animale** ***(valore sociale)***

I dati raccolti consentirebbero di personalizzare le terapie per i pazienti che recano mutazioni LOF di FBXW7. Ciò consentirebbe di minimizzare gli effetti tossici, massimizzare l'efficacia terapeutica e minimizzare il disagio sociale di questi pazienti.

I dati raccolti in merito allo stato metabolico dei tumori permetteranno inoltre di identificare bersagli terapeutici per sopprimere la resistenza alla degradazione di KRAS e valutare l'efficacia della riprogrammazione metabolica come terapia personalizzata. Consentiranno inoltre di evidenziare la predisposizione epigenetica allo sviluppo di resistenza (12).

4. **Eventuale impatto nel settore economico-industriale** *(valore economico)*

Il progetto potrebbe favorire l'abbattimento dei costi di ospedalizzazione per i pazienti recanti mutazioni FBXW7 e che risulterebbero refrattari alla terapia con il platino. Porterebbe alla predizione dell'efficacia terapeutica della chemioterapia convenzionale a partire dalla valutazione dello stato metabolico di PDAC e CRC e di analoghi tumori dipendenti da oncogeni forti. Favorirà la creazione di collaborazioni scientifiche ed eventuale brevetto dell'utilizzo del composto PROTAC. Infine, consentirà la validazione dell'utilizzo di inibitori selettivi PROTAC di regolatori epigenetici su modelli murini pre-clinici.

5. **Eventuali ricadute nell'ambito della formazione** *(valore didattico)*

Non riteniamo possano esserci ricadute in ambito della formazione.

Bibliografia

1. Goodwin, R. A., & Asmis, T. R. (2009). Overview of systemic therapy for colorectal cancer. Clinics in Colon and Rectal Surgery, 22(4), 251–256.
2. He, G., Xie, X., & Siddik, Z. H. (2020). Protein expression profiling identifies differential modulation of homologous recombination by platinum-based antitumor agents. Cancer Chemotherapy and Pharmacology, 85(6), 1129–1140.

3. He, L., Torres-Lockhart, K., Forster, N., Ramakrishnan, S., Greninger, P., Garnett, M. J., … Ellisen, L. W. (2013). Mcl-1 and FBW7 control a dominant survival pathway underlying HDAC and Bcl-2 inhibitor synergy in squamous cell carcinoma. Cancer Discovery, 3(3), 325–337.
4. Korphaisarn, K., Morris, V. K., Overman, M. J., Fogelman, D. R., Kee, B. K., Raghav, K. P. S., … Dasari, A. (2017). FBXW7 missense mutation: A novel negative prognostic factor in metastatic colorectal adenocarcinoma. Oncotarget, 8(24), 39268–39279.
5. Li, N., Babaei-Jadidi, R., Lorenzi, F., Spencer-Dene, B., Clarke, P., Domingo, E., … Nateri, A. S. (2019). An FBXW7-ZEB2 axis links EMT and tumour microenvironment to promote colorectal cancer stem cells and chemoresistance. Oncogenesis, 8(3), 1–17.
6. Di Giorgio, E., Paluvai, H., Dalla, E., Ranzino, L., Renzini, A., Moresi, V., Brancolini, C. (2022). HDAC4 repressive complex controls senescence entrance by antagonizing the epigenetic program of AP-1 and p300 and maintaining the genomic stability, Genome Biology.
7. Dale B, Cheng M, Park KS, Kaniskan HÜ, Xiong Y, Jin J. Advancing targeted protein degradation for cancer therapy. Nat Rev Cancer. 2021 Oct;21(10):638-654. doi: 10.1038/s41568-021-00365-x. Epub 2021 Jun 15. PMID: 34131295; PMCID: PMC8463487.
8. Macabuag, N., Esmieu, W., Breccia, P., Jarvis, R., Blackaby, W., Lazari, O., … Dominguez, C. (2022). Developing HDAC4-Selective Protein Degraders to Investigate the Role of HDAC4 in Huntington's Disease Pathology. Journal of Medicinal Chemistry, 65(18), 12445–12459.
9. Chu TT, Gao N, Li QQ, Chen PG, Yang XF, Chen YX, Zhao YF, Li YM. (2016). Specific Knockdown of Endogenous Tau Protein by Peptide-Directed Ubiquitin-Proteasome Degradation. Cell Chem Biol. Apr 21;23(4):453-61.
10. Bond, MJ, Ling Chu, Dhanusha A. Nalawansha, Ke Li, Crews, CM. (2020). ACS Cent Sci. 2020 Aug 26; 6(8): 1367–1375.
11. Di Giorgio E, Choudury H, Ferino A, Cortolezzis Y, Rapozzi V, Xodo L. (2023) Suppression of the KRAS-NRF2 axis shifts arginine into the phosphocreatine energy system in pancreatic cancer cells, iScience in press.
12. Orouji, E., Raman, A. T., Singh, A. K., Sorokin, A., Arslan, E., Ghosh, A. K., … Rai, K. (2022). Chromatin state dynamics confers specific therapeutic strategies in enhancer subtypes of colorectal cancer. Gut, 71(5), 938–949.

18. Descrizione dei fini del progetto di ricerca (Art. 5, comma 1)

Ricerca di base	**X**
Ricerca traslazionale o applicata	
Prove di tipo regolatorio	
Protezione dell'ambiente naturale nell'interesse della salute o del benessere degli esseri umani o degli animali	

Ricerca finalizzata alla conservazione delle specie	
Insegnamento superiore o formazione professionale	
Indagini medico-legali	
Mantenimento di colonie di animali GM modificati non usati in altre procedure	

19. La ricerca che si intende attuare è già stata effettuata? NO

Se la ricerca è già stata attuata, spiegare perché è scientificamente necessario ripetere l'esperimento.

20. Dichiarazioni riferite all'articolo 13, comma 2 del decreto

20.1

a. **Sostituzione** (*Dichiarazione che la specie animale di cui si farà uso sono quelle a più basso sviluppo neurologico, nonché della mancanza di metodi alternativi, compatibili con l'obiettivo del progetto di ricerca).*

Per questo studio è indispensabile il ricorso ad una sperimentazione in vivo per poter validare i risultati precedentemente ottenuti *in vitro/ex vivo* in modelli 3D di organoidi derivati da tumori di pazienti. È infatti fondamentale poter dimostrare in modello murino la ri-sensibilizzazione alla terapia per i carcinomi colorettali FBXW7 mutati e la valutazione delle terapie metaboliche alternative nei PDAC.

b. **Riduzione** (*Massima riduzione del numero di animali utilizzati, compatibile con gli obiettivi del progetto di ricerca).*

Il numero di animali è stato calcolato per utilizzare il minor numero di animali mantenendo la potenza e la validità statistica dello studio. Il numero di animali per gruppo è stato determinato in base a considerazioni statistiche utilizzando il programma Gpower.

c. **Affinamento** (*Ottimizzazione della metodica per ridurre la sofferenza imposta all'animale durante l'esecuzione delle procedure).*

Il personale coinvolto nell'esperimento possiede la formazione adeguata a eseguire le procedure e le tecniche più adeguate al fine di ridurre al minimo ogni possibile dolore, sofferenza e stress negli animali utilizzati in questo progetto di ricerca. I controlli sono giornalieri, effettuati mattina e sera, da parte di personale competente in grado di riconoscere gli eventuali segni di sofferenza. Durante gli esperimenti, gli animali saranno pesati due volte a settimana in modo da rilevare le variazioni di peso associate alla somministrazione dei composti e saranno osservate le variazioni nella capacità di locomozione e nel consumo di cibo. Gli animali con calo ponderale superiore al 15% verranno sacrificati. I trattamenti, nei vari gruppi, verranno effettuati 2 volte a settimana per due settimane.

**Procedure utilizzate**	**Effetti avversi**	**Metodologia e interventi**	**End-points**
Mantenimento di topi immunocompromessi	Animali sono suscettibili alle infezioni	le gabbie saranno alloggiate in IVC Tecniplast (o armadio ventilato Tecniplast); le manipolazioni avverranno sotto cappa biologica a flusso laminare e tutto il materiale (gabbie, coperchio, filtri, biberon) sarà sterilizzato prima dell'uso. Cibo e segatura saranno gamma irradiati autoclavati e l'acqua filtrata ed autoclavata<br><br>Fornito arricchimento ambientale per ridurre lo stress	Gli animali che mostrano segni di malattia verranno eutanasizzati.
Iniezione sub cutanea di cellule tumorali	Discomfort momentaneo successivo all'iniezione	L'iniezione viene fatta una sola volta. Volume iniettato massimo 0,2 ml.<br><br>Animali monitorati nel periodo immediatamente successivo all'iniezione.	Gli animali che dovessero presentare segni di distress senza rapido recupero verranno esclusi dall'esperimento
Trattamento i.p.	Discomfort momentaneo successivo all'iniezione	Il trattamento viene effettuato per 2 volte a settimana per 4 settimane consecutive. Gli animali vengono monitorati nel periodo immediatamente successivo al trattamento.	Gli animali che dovessero presentare segni di distress senza rapido recupero verranno esclusi dall'esperimento
Crescita del tumore	Può causare discomfort o produrre variazioni sulla locomozione.<br><br>Il tumore utilizzato non ulcera e non metastatizza.	Osservazione giornaliera degli animali, monitoraggio dello stato generale di salute e della crescita tumorale.<br><br>Schema di monitoraggio mediante punteggio per valutare lo stato di benessere dell'animale. (Tabella inclusa sotto)	Gli animali verranno eutanasizzati se il tumore interferisce con il comportamento normale dell'animale, la sua postura o la locomozione.<br><br>In questo esperimento i tumori non eccederanno i 1800 mm^3

Trattamento con nuovi composti	Possono causare diarrea, perdita di peso, anoressia o letargia	Schema di monitoraggio mediante punteggio per valutare lo stato di benessere dell'animale. (Tabella inclusa sotto)	Gli animali vengono eutanasizzati se l'animale non mangia o ha diarrea per più di 48 ore o comunque nel caso in cui il punteggio sia superiore a 3.

Valutazione della sofferenza negli animali da laboratorio. Tabella a punteggi standardizzata per la valutazione della sofferenza negli animali da laboratorio, modificata da Wolfensohn e Lloyd (2003).

Esempi di sistemi di valutazione clinica	Punteggio
**Aspetto**	
Peso corporeo	
Calo ponderale 5-10%	1
Calo ponderale 11-15%	2
Calo ponderale 16-20%	3
Calo ponderale 20% + HEP	HEP
**Stato del mantello**	
Mantello lievemente arruffato	1
Piloerezione lieve	2
Piloerezione marcata	3
**Funzioni corporee**	
Tachipnea (respirazione accellerata)	1
Dispea (respirazione difficoltosa)	3
**Ambiente**	
Feci molle o diarrea	1
Diarrea con sangue	HEP
**Comportamento**	
Teso e nervoso alla manipolazione	1
Distress marcato alla manipolazione, ad es. tremore, vocalizzazione, aggressività	3
**Locomozione**	
Locomozione /postura lievemente anormali	1
Locomozione /postura marcatamente anormali	2
Significativi problemi di mobilità, riluttanza a muoversi	3
Immobilità > 24 h	HEP
**Indicatori specifici della procedura**	
Dimensioni tumore >1,2 cm	1
Dimensioni tumore >1,8 cm	HEP
Ulcerazione del tumore	HEP
Tumore che impedisce il movimento	HEP

Punteggio	Interventi
**1**	Rivedere la frequenza del monitoraggio

**2**	Valutare la possibilità di somministrare cure supplementari, ad es. dose extra di liquidi
**3**	Consultare il veterinario
**HEP**	Attuare punto finale umanitario

MODALITA' DI OSSERVAZIONE, NUMEROSITA' DEL CAMPIONE, RACCOLTA DATI

Osservazione su tutti gli animali almeno una volta al giorno.

20.2

d. **Rapporto danno/beneficio**

Benefici: la neoplasia rappresenta una malattia sociale e cronica che ha costi elevatissimi a carico del sistema sanitario e di conseguenza dell'intera comunità, costituiti da costi diretti derivanti dall'impiego di risorse per la prevenzione, la diagnosi e il trattamento dei tumori e costi indiretti derivanti dalla perdita di produttività dei pazienti e eventualmente anche di pazienti ed amici. I pazienti che recano mutazioni LOF di FBXW7 potrebbero ottenere delle terapie personalizzate con una minimizzazione degli effetti tossici e una massimizzazione dell'efficacia terapeutica. Si potrebbero inoltre identificare bersagli terapeutici alternativi alla degradazione di KRAS e valutare l'efficacia della riprogrammazione metabolica come terapia personalizzata.

Danni: nella descrizione dell'affinamento abbiamo compreso tutti i danni potenziali agli animali derivanti dalle tecniche utilizzate. Nessuna delle procedure è classificabile come grave in base all'allegato 7 del D.Lgs.26/2014. Il responsabile dell'esecuzione dell'esperimento lavora con gli animali da 15 anni ed assieme agli altri membri del team e al veterinario presente nella struttura ha le nozioni scientifiche necessarie per riconoscere gli stati di dolore dell'animale e gli human end points.

21. METODOLOGIA E TECNICA DELL'ESPERIMENTO *(Va spiegato dettagliatamente il protocollo sperimentale, con particolare riferimento alle fasi che prevedono la manipolazione degli animali- frequenza, tipo di trattamenti, prelievi, ecc.)*

Durante la sperimentazione gli animali verranno stabulati in ambienti di stabulazione e laboratori adeguati alla manipolazione in vivo. Tali ambienti sono provvisti di temperatura controllata (20-24°C), di umidità mantenuta al 50-60% e di cicli di luce artificiale con 12 ore di luce alternata a 12

ore di buio. Gli animali avranno accesso illimitato a cibo e acqua. Le gabbie saranno alloggiate in IVC Tecniplast le manipolazioni avverranno sotto cappa biologica a flusso laminare e tutto il materiale (gabbie, coperchio, filtri, biberon) sarà sterilizzato prima dell'uso. Cibo e segatura saranno autoclavati e l'acqua filtrata e autoclavata. Dopo un periodo di acclimatazione verrà impiantato ad ogni topo un microchip in modo da seguire singolarmente la crescita del tumore e la variazione del peso corporeo.

Prove di crescita:

Essendo linee cellulari ingegnerizzazione con la tecnologia CRISPR/Cas9 per esprimere o meno geni che regolano il catabolismo dell'arginina come KRAS, ARG2, CKB, PADI1, PADI3 verranno eseguite prove di crescita per ogni linea in modo da valutare la percentuale di attecchimento e la velocità di crescita in confronto con le cellule wt.

Per ogni linea cellulare si inoculeranno sottocute da 2,5 a 5 x10^6 cellule / topo in 200µl di PBS sterile, a seconda della linea cellulare, secondo lo schema sottostante:

Gruppo	Linea cellulare	Numero topi
1	HCT-116 FBXW7KO+FBXW7 505C	5
2	HCT-116 wt	5

Gruppo	Linea cellulare	Numero topi
1	SW620 BJ mut	5
2	SW620 wt	5

Gruppo	Linea cellulare	Numero topi
1	SW620 E1A	5
2	SW620 wt	5

Gruppo	Linea cellulare	Numero topi
1	SW620 RAS mut	5
2	SW620 wt	5

Gruppo	Linea cellulare	Numero topi
1	SW620 P53DN mut	5
2	SW620 wt	5

In totale per le prove di crescita verranno utilizzati 50 topi.

Protocolli sperimentali:

a) Xenotrapianto di cellule HCT-116 ingegnerizzate con il CRISPR/Cas9 per esprimere FBXW7 505C

Al giorno 0 si inoculeranno sottocute 2 x10^6 cellule HCT-116 FBXW7KO+FBXW7 505C/topo in 200µl di PBS sterile.

La crescita del tumore verrà seguita misurando lo stesso due volte a settimana, a partire da 7 giorni dall'inoculo. Il trattamento inizierà quando i tumori arriveranno alla misura di 80 mm^3 ± 20 mm^3, seguendo lo schema sottostante.

Ogni composto verrà somministrato i.p. 2 volte a settimana per 4 settimane totali di trattamento. Gli animali verranno misurati e pesati due volte a settimana a partire dal giorno di inizio esperimento (giorno 0). Verranno registrati eventuali comportamenti anomali e/o morti avvenute in seguito al trattamento.

Gruppo ID	Composto	Dosaggio mg/kg	Posologia	Via di somministrazio ne	Analisi	Num. di topi
1	Veicolo (PBS + 5% DMSO)	-	2 a settimana per 4 settimane	i.p.	Tumore	10
2	OxPt + PROTAC scramble	5 + 10	2 a settimana per 4 settimane	i.p.	Tumore	10
3	OxPt + PROTAC #11	5 + 10	2 a settimana per 4 settimane	i.p.	Tumore	10
Totale						30

Obiettivo dell'esperimento: misurazione delle dimensioni tumorali, dello stato metabolico dei tumori espiantati, dello stato epigenetico (mappatura dei super enhancer).

b) Xenotrapianto di cellule BJ e valutazione dell'effetto di TDRL-551 (RPA inhibitor) nel sinergizzare con OxPt e valutazione dello stato metabolico ed epigenetico

Al giorno 0 si inoculeranno sottocute 5 x10^6 cellule BJ/ topo in 200µl di PBS sterile.

La crescita del tumore verrà seguita misurando lo stesso due volte a settimana, a partire da 7 giorni dall'inoculo. Il trattamento inizierà quando i tumori arriveranno alla misura di 80 mm^3 ± 20 mm^3, seguendo lo schema sottostante.

Ogni composto verrà somministrato i.p. 2 volte a settimana per 4 settimane totali di trattamento. Gli animali verranno misurati e pesati due volte a settimana a partire dal giorno di inizio esperimento (giorno 0). Verranno registrati eventuali comportamenti anomali e/o morti avvenute in seguito al trattamento.

Gruppo ID	Composto	Dosaggio mg/kg	Posologia	Via di somministrazione	Analisi	Num. di topi
1	Veicolo	0	2 a settimana per 4 settimane	i.p.	Tumore	10
2	OxPt	5	2 a settimana per 4 settimane	i.p.	Tumore	10
3	OxPt + ADI-PEG 20	5 + 5 IU	2 a settimana per 4 settimane	i.p.	Tumore	10
Totale						30

Obiettivo dell'esperimento: misurazione delle dimensioni tumorali, dello stato metabolico dei tumori spiantati e del replication stress.

c) Xenotrapianto di cellule E1A e valutazione dell'effetto di TDRL-551 (RPA inhibitor) nel sinergizzare con OxPt e valutazione dello stato metabolico ed epigenetico

Al giorno 0 si inoculeranno sottocute 5 x10^6 cellule /topo in 200µl di PBS sterile.

La crescita del tumore verrà seguita misurando lo stesso due volte a settimana, a partire da 7 giorni dall'inoculo. Il trattamento inizierà quando i tumori arriveranno alla misura di 80 mm^3 ± 20 mm^3, seguendo lo schema sottostante.

Ogni composto verrà somministrato i.p. 2 volte a settimana per 4 settimane totali di trattamento. Gli animali verranno misurati e pesati due volte a settimana a partire dal giorno di inizio esperimento (giorno 0). Verranno registrati eventuali comportamenti anomali e/o morti avvenute in seguito al

trattamento.

Gruppo ID	Composto	Dosaggio mg/kg	Posologia	Via di somministrazione	Analisi	Num. di topi
1	Veicolo	0	2 a settimana per 4 settimane	i.p.	Tumore	10
2	OxPt	5	2 a settimana per 4 settimane	i.p.	Tumore	10
3	OxPt + ADI-PEG 20	5 + 5 IU	2 a settimana per 4 settimane	i.p.	Tumore	10
Totale						30

d) Xenotrapianto di cellule RAS e valutazione dell'effetto di TDRL-551 (RPA inhibitor) nel sinergizzare con OxPt e valutazione dello stato metabolico ed epigenetico

Al giorno 0 si inoculeranno sottocute 5 x10^6 cellule RAS /topo in 200µl di PBS sterile.

La crescita del tumore verrà seguita misurando lo stesso due volte a settimana, a partire da 7 giorni dall'inoculo. Il trattamento inizierà quando i tumori arriveranno alla misura di 80 mm^3 ± 20 mm^3, seguendo lo schema sottostante.

Ogni composto verrà somministrato i.p. 2 volte a settimana per 4 settimane totali di trattamento. Gli animali verranno misurati e pesati due volte a settimana a partire dal giorno di inizio esperimento (giorno 0). Verranno registrati eventuali comportamenti anomali e/o morti avvenute in seguito al trattamento.

Gruppo ID	Composto	Dosaggio mg/kg	Posologia	Via di somministrazione	Analisi	Num. di topi
1	Veicolo	0	2 a settimana per 4 settimane	i.p.	Tumore	10
2	OxPt	5	2 a settimana per 4 settimane	i.p.	Tumore	10

3	OxPt + ADI-PEG 20	5 + 5 IU	2 a settimana per 4 settimane	i.p.	Tumore	10
Totale						30

e) **Xenotrapianto di cellule P53DN e valutazione dell'effetto di TDRL-551 (RPA inhibitor) nel sinergizzare con OxPt e valutazione dello stato metabolico ed epigenetico**

Al giorno 0 si inoculeranno sottocute 5 x10^6 cellule P53DN/topo in 200µl di PBS sterile.

La crescita del tumore verrà seguita misurando lo stesso due volte a settimana, a partire da 7 giorni dall'inoculo. Il trattamento inizierà quando i tumori arriveranno alla misura di 80 mm^3 ± 20 mm^3, seguendo lo schema sottostante.

Ogni composto verrà somministrato i.p. 2 volte a settimana per 4 settimane totali di trattamento. Gli animali verranno misurati e pesati due volte a settimana a partire dal giorno di inizio esperimento (giorno 0). Verranno registrati eventuali comportamenti anomali e/o morti avvenute in seguito al trattamento.

Gruppo ID	Composto	Dosaggio mg/kg	Posologia	Via di somministrazione	Analisi	Num. di topi
1	Veicolo	0	2 a settimana per 4 settimane	i.p.	Tumore	10
2	OxPt	5	2 a settimana per 4 settimane	i.p.	Tumore	10
3	OxPt + ADI-PEG 20	5 + 5 IU	2 a settimana per 4 settimane	i.p.	Tumore	10
Totale						30

Alla fine di ogni esperimento, gli animali verranno sacrificati mediante dislocazione cervicale e verrà eseguita un'autopsia per la valutazione macroscopica di eventuali modificazioni degli organi

principali (dimensione, colore). I tumori verranno prelevati, pesati e conservati per l'esecuzione di analisi trascrittomiche e proteomiche ex vivo. Gli organi interni verranno conservati per analisi ex vivo.
Se qualcuno degli animali dovesse presentare segni di sofferenza in un qualunque momento dopo la somministrazione si procederà immediatamente all'eutanasia e autopsia. Uno dei dati più rilevanti è rappresentato dalla variazione del peso corporeo degli animali. Di solito si considera segnale di malessere una diminuzione del peso corporeo del 12% valutato sulla curva di crescita. Si tenga presente che topi di 12-20 settimane (quali saranno quelli presi in esame nell'ambito di questo studio) sono nel pieno del loro sviluppo e ci si aspetta che il loro peso aumenti costantemente col passare dei giorni. Quindi la diminuzione del peso corporeo non è da intendersi in senso assoluto, rispetto all'inizio del trattamento, bensì relativamente ai controlli.

Endpoints degli esperimenti:

- Peso corporeo
- Volume del tumore

21.1 Criteri di selezione del campione *(indicare le modalità di selezione del campione, specificando i criteri di inclusione e l'eventuale suddivisione dell'unità campionaria in gruppi).*

Negli esperimenti di efficacia si utilizzeranno topi nudi in modo da permettere l'attecchimento di cellule tumorali umane. Prima di iniziare il trattamento verranno misurati i tumori e si effettuerà una randomizzazione in base alla crescita del tumore, avendo cura di escludere i tumori che risultassero cresciuti più del 30% o meno del 30% rispetto alla media o gli animali nei quali il tumore non è cresciuto.

21.2 Considerazioni statistiche *(descrivere come è stato determinato il numero di animali necessari per lo studio).*

Abbiamo utilizzato il software GPOWER per condurre un'analisi statistica e calcolare il dimensionamento del campione, dove è stato stabilito che il livello di significatività (α) accettabile fosse del 5% ($p<0.05$) e la differenza minima accettabile (δ) tra i gruppi a confronti (gruppo controllo vs gruppo trattato) fosse maggiore/uguale al 30 %. La variabilità (σ) attesa delle misure all'interno dei gruppi è stata assunta dai dati in nostro possesso ottenuti da esperimenti condotti in passato con le stesse linee cellulari indicate nel progetto.

Valido per ogni singolo protocollo sperimentale (a-f):

F tests - ANOVA: Fixed effects, omnibus, one-way

Analysis:	A priori: Compute required sample size	
**Input:**	**Effect size f**	**= 0.7**
**α err prob**	**=**	**0.05**
**Power (1-β err prob)**		**= 0.9**
**Number of groups**		**= 3**
Output:	Noncentrality parameter λ	= 14.7000000
Critical F	=	3.3541308
Numerator df =		3
Denominator df		= 27
**Total sample size**		**= 30**
Actual power =		0.9105726

21.3 Tecnica di esecuzione delle procedure *(descrivere il protocollo sperimentale con particolare riferimento alle procedure indicate di seguito).*

- *prelievi ematici* *NO*
- *produzione di anticorpi* *NO*
- *osservazioni comportamentali* *NO*
- *prelievi di organi e/o tessuti* ***SI***
- *procedure chirurgiche* *NO*
- *inoculo di microrganismi, anche GM* *NO*
- *somministrazioni di farmaci* ***SI***
- *test DL50* *NO*
- *manipolazioni su animali GM* *NO*
- *impianto/induzione di tumori* ***SI***
- *utilizzo di radioisotopi e/o radiazioni* *NO*
- *genotipizzazione animali GM* *NO*
- *altro (specificare)* *NO*

22. Indicare se si prevede di utilizzare le seguenti sostanze chimiche o agenti biologici:

- *Cancerogene* *NO*
- *Mutagene* *NO*

- *Tossiche per la riproduzione*		*NO*
- *Radioattive*		*NO*
- *Antiblastici*		*NO*
- *Colture Cellulari*	*SI*	
- *Microrganismi*		*NO*
- *Microrganismi GM*		*NO*

23. Indicare eventuali rischi per l'operatore.

L'operatore non corre rischi particolari se le buone procedure dello stabulario e le norme di sicurezza sul lavoro saranno applicate correttamente. Sarà cura del responsabile della ricerca informare gli operatori e i ricercatori, implicati nella manipolazione degli animali, delle sostanze chimiche e dei tessuti, in merito ai rischi e alle corrette procedure di prevenzione degli stessi. Verranno forniti i DPI più opportuni come individuati dal RSPP.

Tutte le operazioni verranno effettuate sotto cappa a flusso laminare.

Il personale che attenderà agli esperimenti indosserà, come individuato dal RSPP camice in tyvek monouso, calzature e sovrascarpe di uso esclusivo all'interno dello stabulario, doppi guanti in lattice, mascherina FFP2, occhiali di protezione. Tutte le operazioni di caricamento delle siringhe e trattamento degli animali avviene sotto cappa mobile (Techniplast).

24. Effetti avversi e misure per ridurre, evitare ed attenuare qualsiasi forma di sofferenza per l'animale dalla nascita alla morte.

Descrivere i potenziali effetti negativi che la procedura può determinare sull'animale (es. dolore, stress, perdita di peso, febbre, anemia, deficit neurologici, comportamenti anomali o altri sintomi clinici di stress acuto o cronico o deficienze nutrizionali).

Il progetto prevede procedure standard di sperimentazione animale. In particolare, sono previsti inoculi sottocutanei di cellule tumorali umane in topi immunocompromessi e trattamenti farmacologici.

Riguardo l'iniezione sottocutanea in topi immunocompromessi di cellule tumorali, gli animali saranno soppressi con metodi umanitari se verranno riscontrati distress o disagio più che lievi, in

mancanza di recupero rapido, dopo l'iniezione (eventualità molto rara). Dopo l'inoculo gli animali verranno giornalmente controllati e osservati, e al primo eventuale segno di sofferenza l'esperimento verrà interrotto. In particolare, gli animali che dovessero presentare segni di sofferenza verranno soppressi mediante dislocazione cervicale.

L'esperimento terminerà quando il tumore raggiungerà una grandezza massima di 1,8 cm^3, gli animali saranno quindi sacrificati attraverso dislocazione cervicale. Nella nostra esperienza tali dimensioni non hanno mai dato alcuna sofferenza agli animali. Pertanto, noi riteniamo che il livello di sofferenza dell'animale è da considerarsi moderato e non grave.

Infine, la somministrazione i.p. degli agenti terapeutici sarà ripetuta per due settimane, due volte a settimane, ma dai dati pregressi per la dose e la frequenza di somministrazione non prevediamo che dia effetti tossici in quanto, come menzionato precedentemente, i trattamenti farmacologici previsti rientrano nell'ambito della routine traslazionale dell'oncologia molecolare. Gli animali verranno monitorati giornalmente secondo la tabella inserita in precedenza, gli animali che dovessero presentare segni di sofferenza o con un calo ponderale che superi il 15% del peso corporeo iniziale verranno soppressi mediante dislocazione cervicale.

Indicare se questi effetti possono essere ridotti e/o annullati con la somministrazione di analgesici o altri farmaci e in caso contrario, indicare perché ciò non è possibile.

Non è possibile ridurre e/o annullare i suddetti effetti del trattamento in quanto la somministrazione di analgesici o altri farmaci andrebbero ad interferire e a modificare sostanzialmente i risultati dello studio. Si ribadisce che lo stato di salute degli animali verrà monitorato costantemente e, al fine di evitare inutili sofferenze, verrà applicato uno "scoring" giornaliero, secondo la tabella acclusa al punto 20 e 21.

25. La morte dell'animale è l'evento finale (endpoint) della procedura? NO

La morte come endpoint si manifesta nei test di tossicità acuta, nella valutazione della patogenicità degli agenti infettivi, nei test di neutralizzazione per le tossine e in altri studi nei quali gli animali non possono essere sottoposti ad eutanasia, ma la morte deve essere il diretto risultato della procedura sperimentale.

Se SI Assicurare il rispetto delle condizioni di cui all'articolo 12, comma 3

26. Proposta di classificazione della gravità delle procedure ai sensi dell'articolo 15 e Allegato VII

Non risveglio

Lievi

<u>Moderate</u>

Gravi

26.1.IX. Specie e Gravità

Codice Specie	*Specie*	*Numero Animali*	*Descrizione Gravità*
A1	Mus musculus	260	moderate

27. USO DI ANESTESIA DURANTE LE PROCEDURE SPERIMENTALI

NO

28. SE SÌ, INDICARE MODALITÀ E TIPO DI ANESTESIA/ANALGESIA

Specie	*Farmaco*	*Dose*	*Via somministrazione*

29. MODALITÀ E TIPO DELL'EVENTUALE TERAPIA ANTIDOLORIFICA

Specie	*Farmaco*	*Dose*	*Via somministrazione*

30. INDICARE L'EVENTUALE MODALITÀ DI SOPPRESSIONE DEGLI ANIMALI

Specie	*Metodo chimico o fisico*	*Sostanza (se del caso)*	*Dose*	*Via somministrazione*
Mus Musculus	*Fisico, dislocazione cervicale*			

31. Il Metodo di soppressione è compreso nell'elenco di cui all'Allegato IV

SI

Se NO giustificare scientificamente la necessità di ricorrere all'autorizzazione per tale metodo

32. AL TERMINE DELLE PROCEDURE SPERIMENTALI GLI ANIMALI SARANNO RIUTILIZZATI NEL RISPETTO DELLE CONDIZIONI DI CUI ALL'ARTICOLO 16

NO

32.1. IX. Specie Animali e Destino

Codice Specie	*Specie*	*Numero Animali*	*Descrizione Destino*
A1	Mus musculus	260	soppressione

33. COLLABORAZIONI

Dott. Di Giorgio Università degli Studi di Udine

34. FINANZIAMENTI

35. Curriculum Vitae   RESPONSABILE PROGETTO DI RICERCA

36. DOCUMENTAZIONE DA ALLEGARE

36.1 Parere dell'Organismo preposto al Benessere Animale

36.2 Sintesi non tecnica del progetto (ai sensi dell'articolo 34 e conforme all'Allegato IX)

36.3 Dichiarazione del responsabile del progetto di ricerca resa ai sensi dell'articolo 46, comma 1, lettera aa) del D.P.R. 28 dicembre 2000 "Disposizioni legislative in materia di documentazione amministrativa" circa l'assenza di sentenze definitive, ovvero rese ai sensi dell'articolo 444 c.p.p. per uno dei reati di cui agli articoli 544-bis, 544-ter del codice penale, nonché per quelli di cui agli articoli 4 e 5 della legge 4 novembre 2010, n. 201.

Luogo e data

Il Responsabile del Progetto di ricerca

Il Veterinario Designato

Il Responsabile del Benessere animale

Dichiarazione consenso trattamento dati personali (Firma Responsabile del progetto di ricerca) secondo normativa vigente.

Il Responsabile del Progetto di ricerca
