# Supplementary figures and images for: Class IIa HDACs forced degradation allows resensitization of oxaliplatin‐resistant FBXW7‐mutated colorectal cancer
Source: Mol Oncol. 2025 Oct 31;20(3):637–67. doi: 10.1002/1878-0261.70152 (PMC13042376; doi:10.1002/1878-0261.70152)

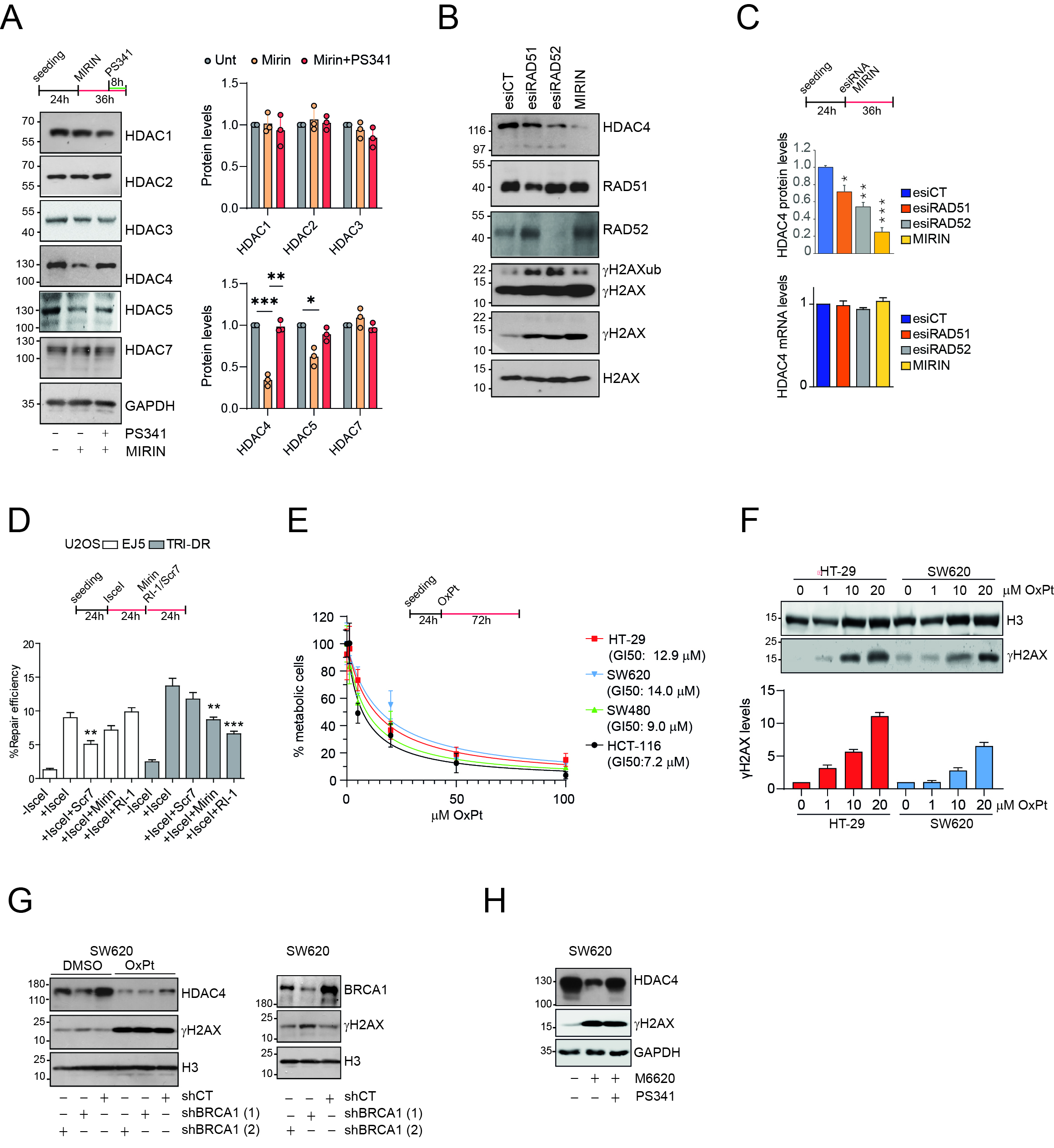

Supplement: Supplementary file 1 — Fig. S1. Inhibition of homologous recombination (HR) repair leads to a reduction in HDAC4 protein levels across various cellular contexts. Fig. S2. Identification of the E3 ligases involved in HDAC4 degradation through in silico and in vitro screenings. Fig. S3. Characterization of FBXW7−/− cells and FBXW7 R505C. Fig. S4. HDAC4 forced degradation or silencing increased OXPT cytotoxicity. Fig. S5. Identification of a signature of genes under the control of HDAC4. Fig. S6. Characterization of PDOs. Fig. S7. Characterization of the epigenetic response driven by HDAC4. Fig. S8. Dissection of the epigenetic response driven by HDAC4. Fig. S9. Original images used for the composition of the immunoblot panels in the main figures. Fig. S10. Original images used for the composition of the immunoblot panels in the supplementary figures. Table S1. Protein expression levels (z‐score) of HDAC4, HDAC5 and 365 E3 ligases available for the indicated 375 cancer cell lines of the Cancer Cell Line Encyclopedia. Table S2. Characteristics of CRC patients whose biopsies were used for the TMA. Table S3. .bed files of the SEs identified in HCT‐116 cells. Table S4. .bed files of the SEs belonging to group 1 and 2 and those directly bound by HDAC4. Table S5. Minimal signature of 116 genes associated to group 1 and 2 of SEs. Table S6. TCGA sample ID of CRC patients bearing FBXW7 LOF. Table S7. List and sequences of primers used for this study. Table S8. Raw data for in vivo experiments. Video S1. Time‐lapse video microscopy of PDM‐96 expressing pLS‐mP‐NR4A2‐EGFP treated with OXPT 20 μm at time 0. Video S2. Time‐lapse video microscopy of PDM‐96 expressing pLS‐mP‐NR4A2‐EGFP treated with OXPT 20 μm + #11 1 μm at time 0. Video S3. Time‐lapse video microscopy of PDM‐96 expressing pLS‐mP‐RNF43‐EGFP treated with OXPT 20 μm at time 0. Video S4. Time‐lapse video microscopy of PDM‐96 expressing pLS‐mP‐RNF43‐EGFP treated with OXPT 20 μm + #11 1 μm at time 0. File S1. Ethical documentation. [file MOL2-20-637-s001.zip › mol270152-sup-0013-FigS1.jpg]

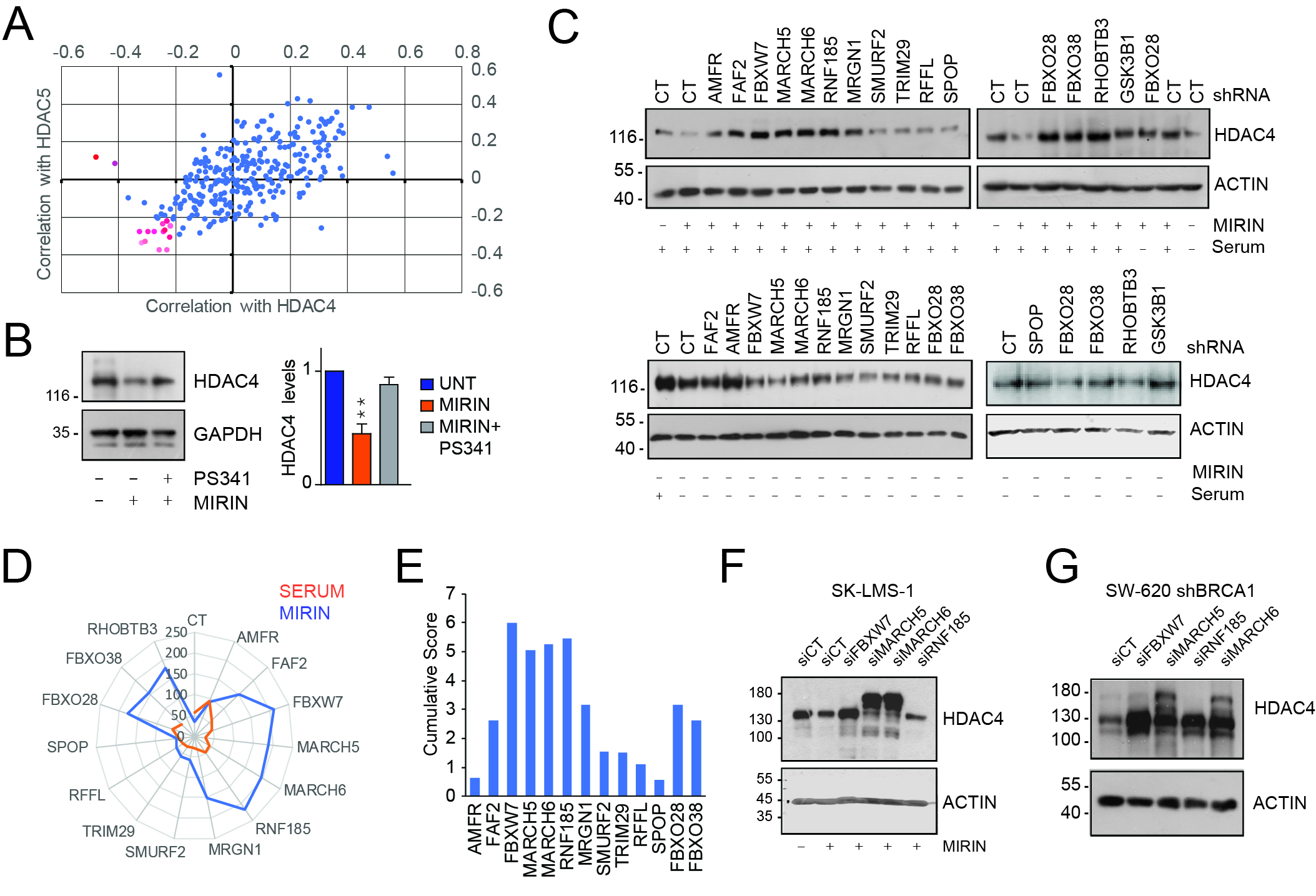

Supplement: Supplementary file 1 — Fig. S1. Inhibition of homologous recombination (HR) repair leads to a reduction in HDAC4 protein levels across various cellular contexts. Fig. S2. Identification of the E3 ligases involved in HDAC4 degradation through in silico and in vitro screenings. Fig. S3. Characterization of FBXW7−/− cells and FBXW7 R505C. Fig. S4. HDAC4 forced degradation or silencing increased OXPT cytotoxicity. Fig. S5. Identification of a signature of genes under the control of HDAC4. Fig. S6. Characterization of PDOs. Fig. S7. Characterization of the epigenetic response driven by HDAC4. Fig. S8. Dissection of the epigenetic response driven by HDAC4. Fig. S9. Original images used for the composition of the immunoblot panels in the main figures. Fig. S10. Original images used for the composition of the immunoblot panels in the supplementary figures. Table S1. Protein expression levels (z‐score) of HDAC4, HDAC5 and 365 E3 ligases available for the indicated 375 cancer cell lines of the Cancer Cell Line Encyclopedia. Table S2. Characteristics of CRC patients whose biopsies were used for the TMA. Table S3. .bed files of the SEs identified in HCT‐116 cells. Table S4. .bed files of the SEs belonging to group 1 and 2 and those directly bound by HDAC4. Table S5. Minimal signature of 116 genes associated to group 1 and 2 of SEs. Table S6. TCGA sample ID of CRC patients bearing FBXW7 LOF. Table S7. List and sequences of primers used for this study. Table S8. Raw data for in vivo experiments. Video S1. Time‐lapse video microscopy of PDM‐96 expressing pLS‐mP‐NR4A2‐EGFP treated with OXPT 20 μm at time 0. Video S2. Time‐lapse video microscopy of PDM‐96 expressing pLS‐mP‐NR4A2‐EGFP treated with OXPT 20 μm + #11 1 μm at time 0. Video S3. Time‐lapse video microscopy of PDM‐96 expressing pLS‐mP‐RNF43‐EGFP treated with OXPT 20 μm at time 0. Video S4. Time‐lapse video microscopy of PDM‐96 expressing pLS‐mP‐RNF43‐EGFP treated with OXPT 20 μm + #11 1 μm at time 0. File S1. Ethical documentation. [file MOL2-20-637-s001.zip › mol270152-sup-0014-FigS2.jpg]

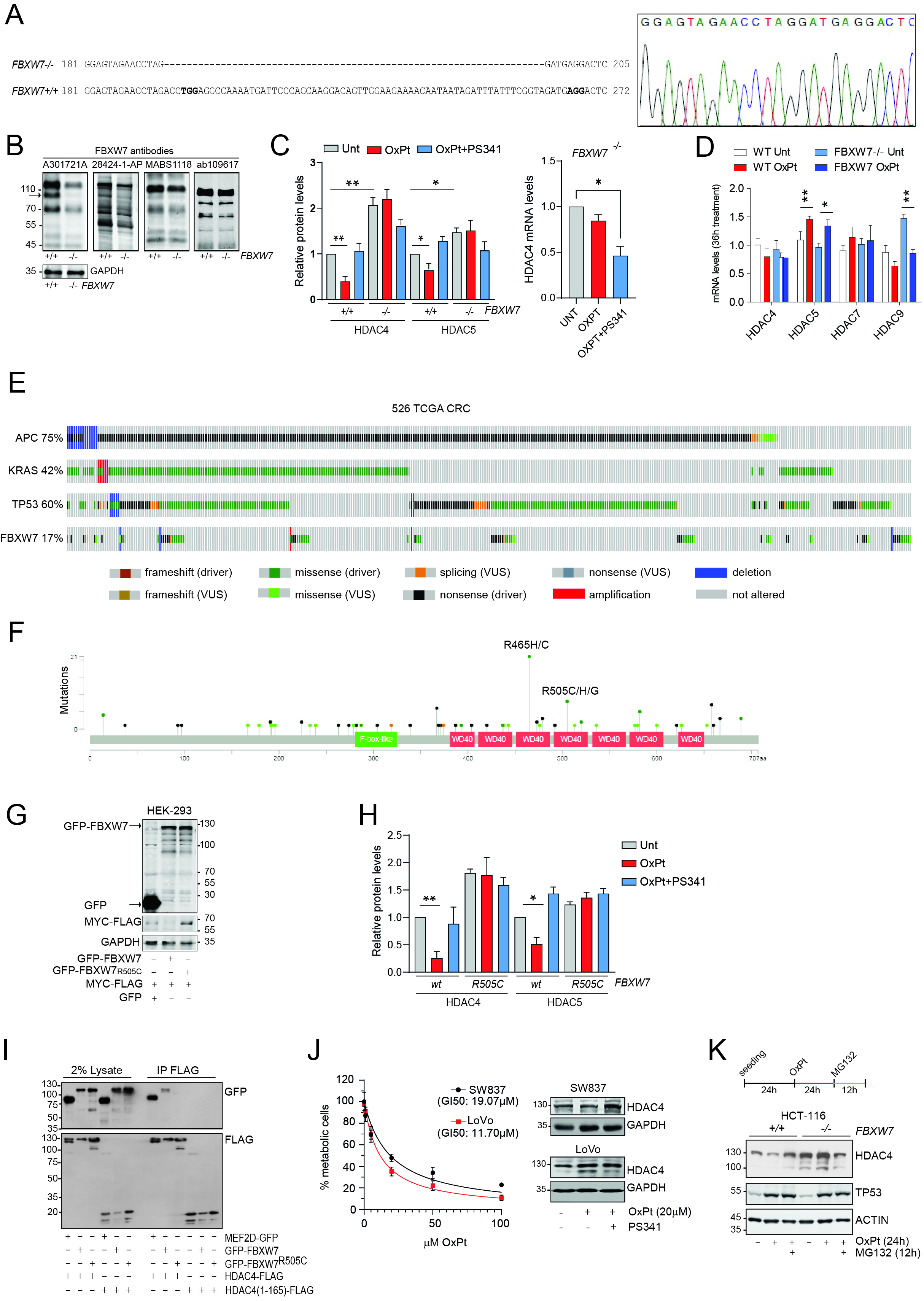

Supplement: Supplementary file 1 — Fig. S1. Inhibition of homologous recombination (HR) repair leads to a reduction in HDAC4 protein levels across various cellular contexts. Fig. S2. Identification of the E3 ligases involved in HDAC4 degradation through in silico and in vitro screenings. Fig. S3. Characterization of FBXW7−/− cells and FBXW7 R505C. Fig. S4. HDAC4 forced degradation or silencing increased OXPT cytotoxicity. Fig. S5. Identification of a signature of genes under the control of HDAC4. Fig. S6. Characterization of PDOs. Fig. S7. Characterization of the epigenetic response driven by HDAC4. Fig. S8. Dissection of the epigenetic response driven by HDAC4. Fig. S9. Original images used for the composition of the immunoblot panels in the main figures. Fig. S10. Original images used for the composition of the immunoblot panels in the supplementary figures. Table S1. Protein expression levels (z‐score) of HDAC4, HDAC5 and 365 E3 ligases available for the indicated 375 cancer cell lines of the Cancer Cell Line Encyclopedia. Table S2. Characteristics of CRC patients whose biopsies were used for the TMA. Table S3. .bed files of the SEs identified in HCT‐116 cells. Table S4. .bed files of the SEs belonging to group 1 and 2 and those directly bound by HDAC4. Table S5. Minimal signature of 116 genes associated to group 1 and 2 of SEs. Table S6. TCGA sample ID of CRC patients bearing FBXW7 LOF. Table S7. List and sequences of primers used for this study. Table S8. Raw data for in vivo experiments. Video S1. Time‐lapse video microscopy of PDM‐96 expressing pLS‐mP‐NR4A2‐EGFP treated with OXPT 20 μm at time 0. Video S2. Time‐lapse video microscopy of PDM‐96 expressing pLS‐mP‐NR4A2‐EGFP treated with OXPT 20 μm + #11 1 μm at time 0. Video S3. Time‐lapse video microscopy of PDM‐96 expressing pLS‐mP‐RNF43‐EGFP treated with OXPT 20 μm at time 0. Video S4. Time‐lapse video microscopy of PDM‐96 expressing pLS‐mP‐RNF43‐EGFP treated with OXPT 20 μm + #11 1 μm at time 0. File S1. Ethical documentation. [file MOL2-20-637-s001.zip › mol270152-sup-0015-FigS3.jpg]

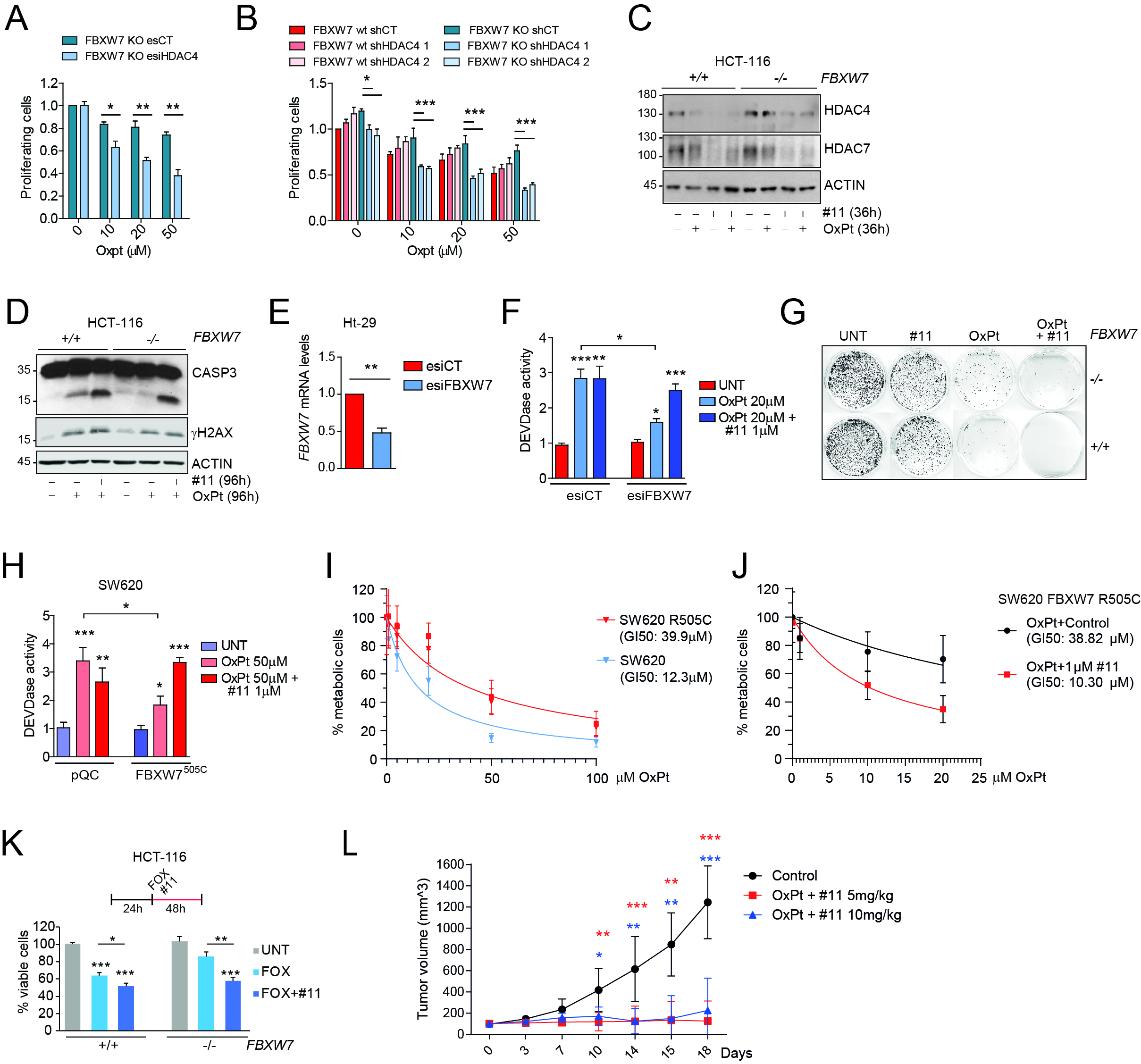

Supplement: Supplementary file 1 — Fig. S1. Inhibition of homologous recombination (HR) repair leads to a reduction in HDAC4 protein levels across various cellular contexts. Fig. S2. Identification of the E3 ligases involved in HDAC4 degradation through in silico and in vitro screenings. Fig. S3. Characterization of FBXW7−/− cells and FBXW7 R505C. Fig. S4. HDAC4 forced degradation or silencing increased OXPT cytotoxicity. Fig. S5. Identification of a signature of genes under the control of HDAC4. Fig. S6. Characterization of PDOs. Fig. S7. Characterization of the epigenetic response driven by HDAC4. Fig. S8. Dissection of the epigenetic response driven by HDAC4. Fig. S9. Original images used for the composition of the immunoblot panels in the main figures. Fig. S10. Original images used for the composition of the immunoblot panels in the supplementary figures. Table S1. Protein expression levels (z‐score) of HDAC4, HDAC5 and 365 E3 ligases available for the indicated 375 cancer cell lines of the Cancer Cell Line Encyclopedia. Table S2. Characteristics of CRC patients whose biopsies were used for the TMA. Table S3. .bed files of the SEs identified in HCT‐116 cells. Table S4. .bed files of the SEs belonging to group 1 and 2 and those directly bound by HDAC4. Table S5. Minimal signature of 116 genes associated to group 1 and 2 of SEs. Table S6. TCGA sample ID of CRC patients bearing FBXW7 LOF. Table S7. List and sequences of primers used for this study. Table S8. Raw data for in vivo experiments. Video S1. Time‐lapse video microscopy of PDM‐96 expressing pLS‐mP‐NR4A2‐EGFP treated with OXPT 20 μm at time 0. Video S2. Time‐lapse video microscopy of PDM‐96 expressing pLS‐mP‐NR4A2‐EGFP treated with OXPT 20 μm + #11 1 μm at time 0. Video S3. Time‐lapse video microscopy of PDM‐96 expressing pLS‐mP‐RNF43‐EGFP treated with OXPT 20 μm at time 0. Video S4. Time‐lapse video microscopy of PDM‐96 expressing pLS‐mP‐RNF43‐EGFP treated with OXPT 20 μm + #11 1 μm at time 0. File S1. Ethical documentation. [file MOL2-20-637-s001.zip › mol270152-sup-0016-FigS4.jpg]

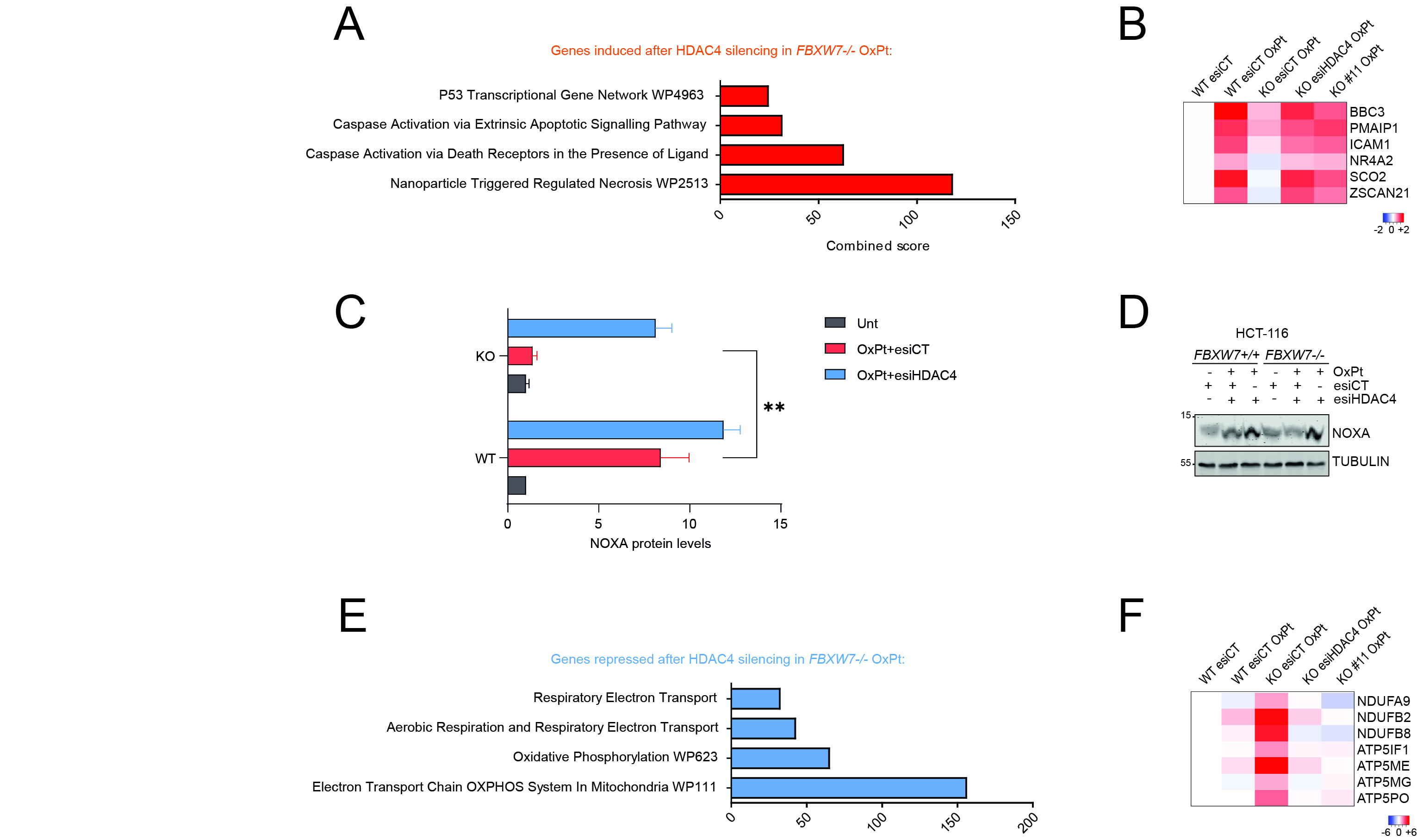

Supplement: Supplementary file 1 — Fig. S1. Inhibition of homologous recombination (HR) repair leads to a reduction in HDAC4 protein levels across various cellular contexts. Fig. S2. Identification of the E3 ligases involved in HDAC4 degradation through in silico and in vitro screenings. Fig. S3. Characterization of FBXW7−/− cells and FBXW7 R505C. Fig. S4. HDAC4 forced degradation or silencing increased OXPT cytotoxicity. Fig. S5. Identification of a signature of genes under the control of HDAC4. Fig. S6. Characterization of PDOs. Fig. S7. Characterization of the epigenetic response driven by HDAC4. Fig. S8. Dissection of the epigenetic response driven by HDAC4. Fig. S9. Original images used for the composition of the immunoblot panels in the main figures. Fig. S10. Original images used for the composition of the immunoblot panels in the supplementary figures. Table S1. Protein expression levels (z‐score) of HDAC4, HDAC5 and 365 E3 ligases available for the indicated 375 cancer cell lines of the Cancer Cell Line Encyclopedia. Table S2. Characteristics of CRC patients whose biopsies were used for the TMA. Table S3. .bed files of the SEs identified in HCT‐116 cells. Table S4. .bed files of the SEs belonging to group 1 and 2 and those directly bound by HDAC4. Table S5. Minimal signature of 116 genes associated to group 1 and 2 of SEs. Table S6. TCGA sample ID of CRC patients bearing FBXW7 LOF. Table S7. List and sequences of primers used for this study. Table S8. Raw data for in vivo experiments. Video S1. Time‐lapse video microscopy of PDM‐96 expressing pLS‐mP‐NR4A2‐EGFP treated with OXPT 20 μm at time 0. Video S2. Time‐lapse video microscopy of PDM‐96 expressing pLS‐mP‐NR4A2‐EGFP treated with OXPT 20 μm + #11 1 μm at time 0. Video S3. Time‐lapse video microscopy of PDM‐96 expressing pLS‐mP‐RNF43‐EGFP treated with OXPT 20 μm at time 0. Video S4. Time‐lapse video microscopy of PDM‐96 expressing pLS‐mP‐RNF43‐EGFP treated with OXPT 20 μm + #11 1 μm at time 0. File S1. Ethical documentation. [file MOL2-20-637-s001.zip › mol270152-sup-0017-FigS5.jpg]

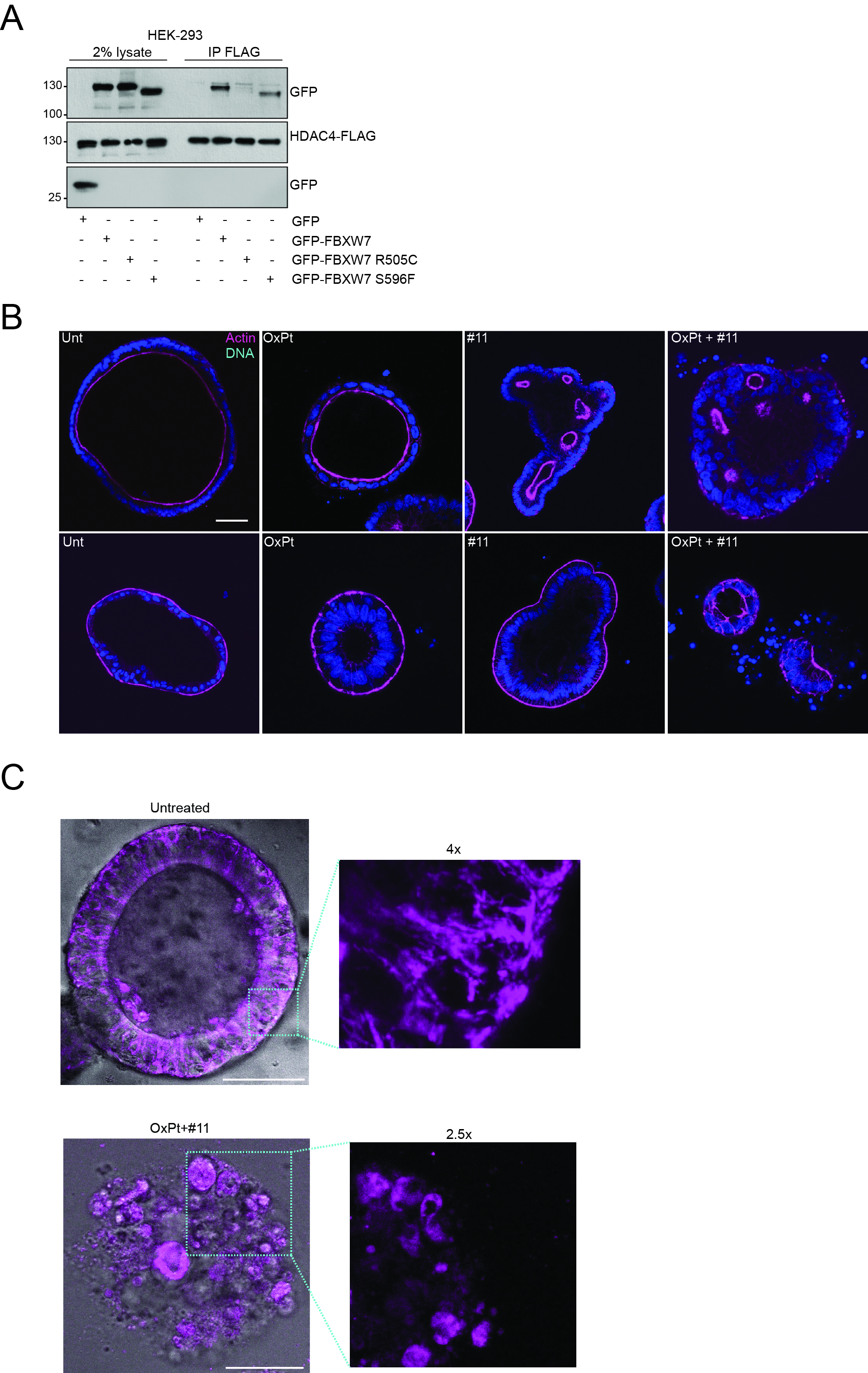

Supplement: Supplementary file 1 — Fig. S1. Inhibition of homologous recombination (HR) repair leads to a reduction in HDAC4 protein levels across various cellular contexts. Fig. S2. Identification of the E3 ligases involved in HDAC4 degradation through in silico and in vitro screenings. Fig. S3. Characterization of FBXW7−/− cells and FBXW7 R505C. Fig. S4. HDAC4 forced degradation or silencing increased OXPT cytotoxicity. Fig. S5. Identification of a signature of genes under the control of HDAC4. Fig. S6. Characterization of PDOs. Fig. S7. Characterization of the epigenetic response driven by HDAC4. Fig. S8. Dissection of the epigenetic response driven by HDAC4. Fig. S9. Original images used for the composition of the immunoblot panels in the main figures. Fig. S10. Original images used for the composition of the immunoblot panels in the supplementary figures. Table S1. Protein expression levels (z‐score) of HDAC4, HDAC5 and 365 E3 ligases available for the indicated 375 cancer cell lines of the Cancer Cell Line Encyclopedia. Table S2. Characteristics of CRC patients whose biopsies were used for the TMA. Table S3. .bed files of the SEs identified in HCT‐116 cells. Table S4. .bed files of the SEs belonging to group 1 and 2 and those directly bound by HDAC4. Table S5. Minimal signature of 116 genes associated to group 1 and 2 of SEs. Table S6. TCGA sample ID of CRC patients bearing FBXW7 LOF. Table S7. List and sequences of primers used for this study. Table S8. Raw data for in vivo experiments. Video S1. Time‐lapse video microscopy of PDM‐96 expressing pLS‐mP‐NR4A2‐EGFP treated with OXPT 20 μm at time 0. Video S2. Time‐lapse video microscopy of PDM‐96 expressing pLS‐mP‐NR4A2‐EGFP treated with OXPT 20 μm + #11 1 μm at time 0. Video S3. Time‐lapse video microscopy of PDM‐96 expressing pLS‐mP‐RNF43‐EGFP treated with OXPT 20 μm at time 0. Video S4. Time‐lapse video microscopy of PDM‐96 expressing pLS‐mP‐RNF43‐EGFP treated with OXPT 20 μm + #11 1 μm at time 0. File S1. Ethical documentation. [file MOL2-20-637-s001.zip › mol270152-sup-0018-FigS6.jpg]

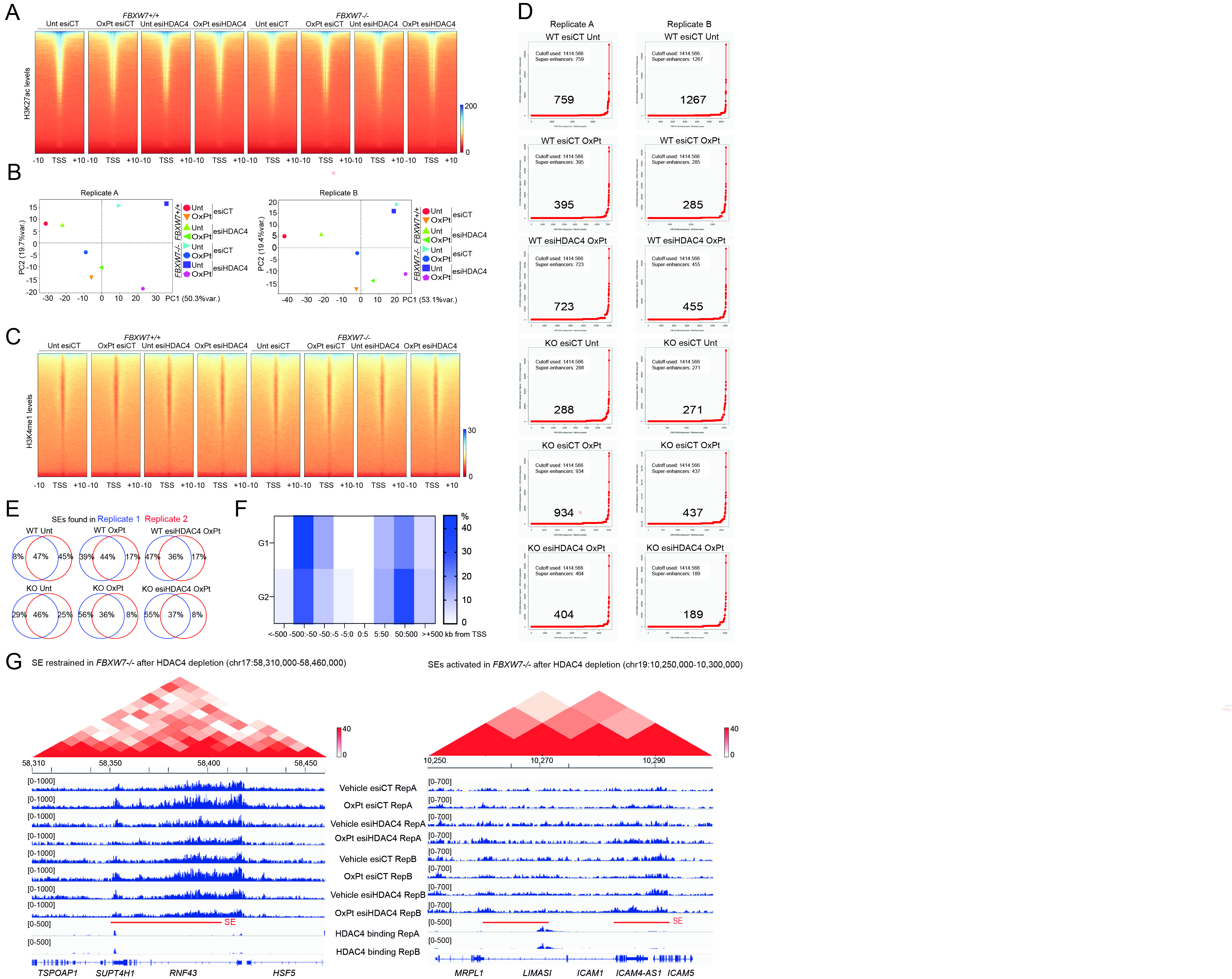

Supplement: Supplementary file 1 — Fig. S1. Inhibition of homologous recombination (HR) repair leads to a reduction in HDAC4 protein levels across various cellular contexts. Fig. S2. Identification of the E3 ligases involved in HDAC4 degradation through in silico and in vitro screenings. Fig. S3. Characterization of FBXW7−/− cells and FBXW7 R505C. Fig. S4. HDAC4 forced degradation or silencing increased OXPT cytotoxicity. Fig. S5. Identification of a signature of genes under the control of HDAC4. Fig. S6. Characterization of PDOs. Fig. S7. Characterization of the epigenetic response driven by HDAC4. Fig. S8. Dissection of the epigenetic response driven by HDAC4. Fig. S9. Original images used for the composition of the immunoblot panels in the main figures. Fig. S10. Original images used for the composition of the immunoblot panels in the supplementary figures. Table S1. Protein expression levels (z‐score) of HDAC4, HDAC5 and 365 E3 ligases available for the indicated 375 cancer cell lines of the Cancer Cell Line Encyclopedia. Table S2. Characteristics of CRC patients whose biopsies were used for the TMA. Table S3. .bed files of the SEs identified in HCT‐116 cells. Table S4. .bed files of the SEs belonging to group 1 and 2 and those directly bound by HDAC4. Table S5. Minimal signature of 116 genes associated to group 1 and 2 of SEs. Table S6. TCGA sample ID of CRC patients bearing FBXW7 LOF. Table S7. List and sequences of primers used for this study. Table S8. Raw data for in vivo experiments. Video S1. Time‐lapse video microscopy of PDM‐96 expressing pLS‐mP‐NR4A2‐EGFP treated with OXPT 20 μm at time 0. Video S2. Time‐lapse video microscopy of PDM‐96 expressing pLS‐mP‐NR4A2‐EGFP treated with OXPT 20 μm + #11 1 μm at time 0. Video S3. Time‐lapse video microscopy of PDM‐96 expressing pLS‐mP‐RNF43‐EGFP treated with OXPT 20 μm at time 0. Video S4. Time‐lapse video microscopy of PDM‐96 expressing pLS‐mP‐RNF43‐EGFP treated with OXPT 20 μm + #11 1 μm at time 0. File S1. Ethical documentation. [file MOL2-20-637-s001.zip › mol270152-sup-0019-FigS7.jpg]

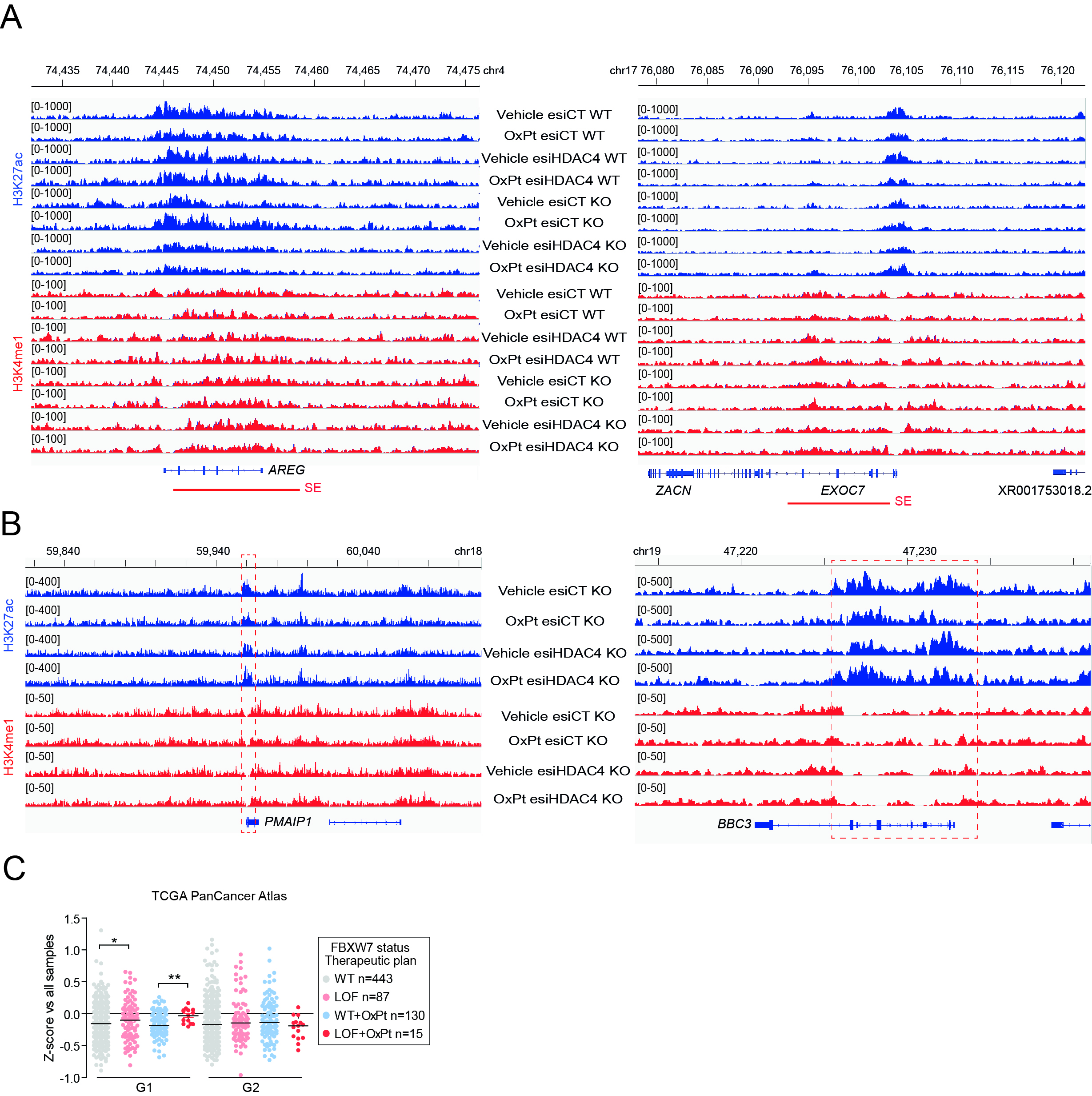

Supplement: Supplementary file 1 — Fig. S1. Inhibition of homologous recombination (HR) repair leads to a reduction in HDAC4 protein levels across various cellular contexts. Fig. S2. Identification of the E3 ligases involved in HDAC4 degradation through in silico and in vitro screenings. Fig. S3. Characterization of FBXW7−/− cells and FBXW7 R505C. Fig. S4. HDAC4 forced degradation or silencing increased OXPT cytotoxicity. Fig. S5. Identification of a signature of genes under the control of HDAC4. Fig. S6. Characterization of PDOs. Fig. S7. Characterization of the epigenetic response driven by HDAC4. Fig. S8. Dissection of the epigenetic response driven by HDAC4. Fig. S9. Original images used for the composition of the immunoblot panels in the main figures. Fig. S10. Original images used for the composition of the immunoblot panels in the supplementary figures. Table S1. Protein expression levels (z‐score) of HDAC4, HDAC5 and 365 E3 ligases available for the indicated 375 cancer cell lines of the Cancer Cell Line Encyclopedia. Table S2. Characteristics of CRC patients whose biopsies were used for the TMA. Table S3. .bed files of the SEs identified in HCT‐116 cells. Table S4. .bed files of the SEs belonging to group 1 and 2 and those directly bound by HDAC4. Table S5. Minimal signature of 116 genes associated to group 1 and 2 of SEs. Table S6. TCGA sample ID of CRC patients bearing FBXW7 LOF. Table S7. List and sequences of primers used for this study. Table S8. Raw data for in vivo experiments. Video S1. Time‐lapse video microscopy of PDM‐96 expressing pLS‐mP‐NR4A2‐EGFP treated with OXPT 20 μm at time 0. Video S2. Time‐lapse video microscopy of PDM‐96 expressing pLS‐mP‐NR4A2‐EGFP treated with OXPT 20 μm + #11 1 μm at time 0. Video S3. Time‐lapse video microscopy of PDM‐96 expressing pLS‐mP‐RNF43‐EGFP treated with OXPT 20 μm at time 0. Video S4. Time‐lapse video microscopy of PDM‐96 expressing pLS‐mP‐RNF43‐EGFP treated with OXPT 20 μm + #11 1 μm at time 0. File S1. Ethical documentation. [file MOL2-20-637-s001.zip › mol270152-sup-0020-FigS8.jpg]

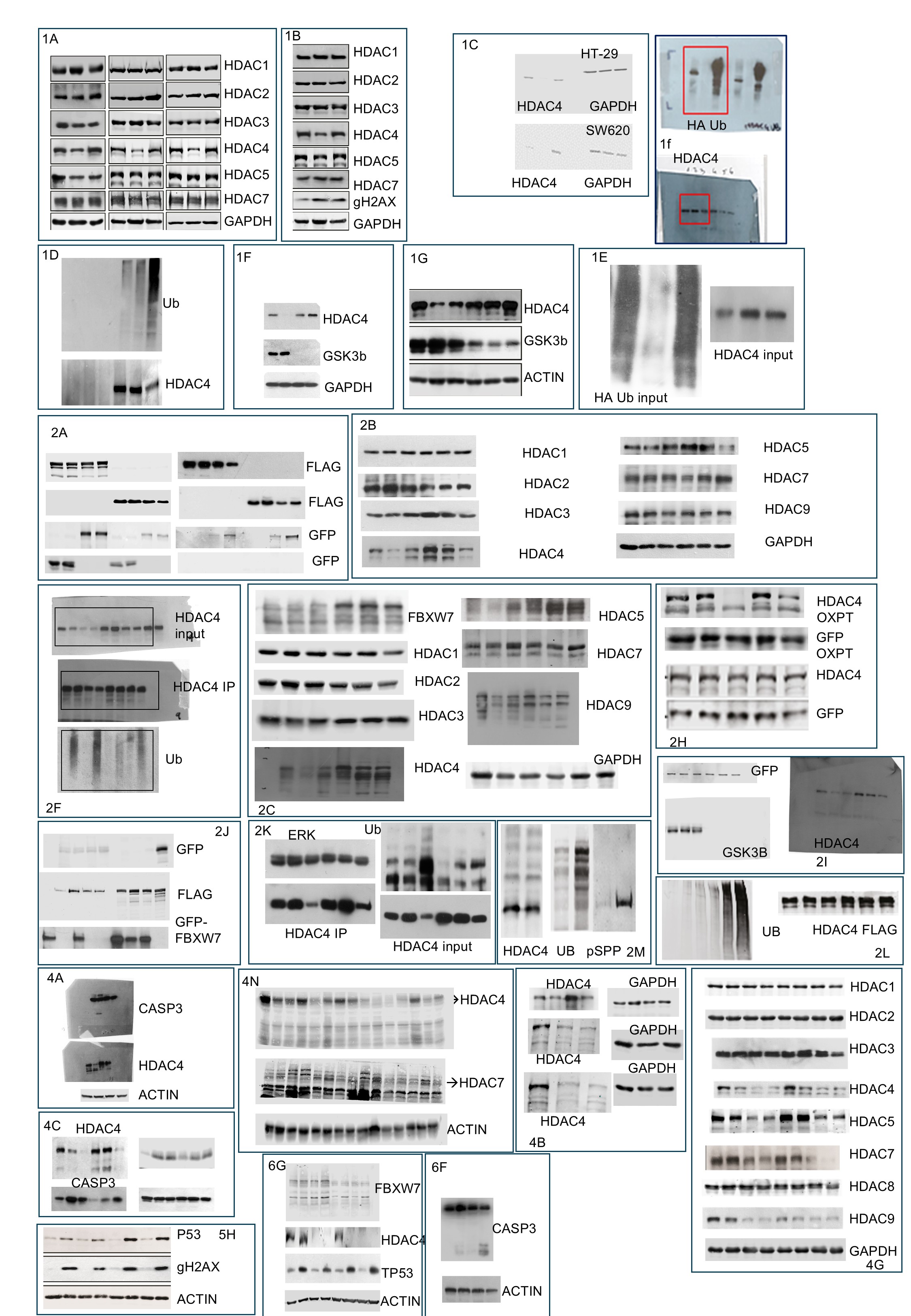

Supplement: Supplementary file 1 — Fig. S1. Inhibition of homologous recombination (HR) repair leads to a reduction in HDAC4 protein levels across various cellular contexts. Fig. S2. Identification of the E3 ligases involved in HDAC4 degradation through in silico and in vitro screenings. Fig. S3. Characterization of FBXW7−/− cells and FBXW7 R505C. Fig. S4. HDAC4 forced degradation or silencing increased OXPT cytotoxicity. Fig. S5. Identification of a signature of genes under the control of HDAC4. Fig. S6. Characterization of PDOs. Fig. S7. Characterization of the epigenetic response driven by HDAC4. Fig. S8. Dissection of the epigenetic response driven by HDAC4. Fig. S9. Original images used for the composition of the immunoblot panels in the main figures. Fig. S10. Original images used for the composition of the immunoblot panels in the supplementary figures. Table S1. Protein expression levels (z‐score) of HDAC4, HDAC5 and 365 E3 ligases available for the indicated 375 cancer cell lines of the Cancer Cell Line Encyclopedia. Table S2. Characteristics of CRC patients whose biopsies were used for the TMA. Table S3. .bed files of the SEs identified in HCT‐116 cells. Table S4. .bed files of the SEs belonging to group 1 and 2 and those directly bound by HDAC4. Table S5. Minimal signature of 116 genes associated to group 1 and 2 of SEs. Table S6. TCGA sample ID of CRC patients bearing FBXW7 LOF. Table S7. List and sequences of primers used for this study. Table S8. Raw data for in vivo experiments. Video S1. Time‐lapse video microscopy of PDM‐96 expressing pLS‐mP‐NR4A2‐EGFP treated with OXPT 20 μm at time 0. Video S2. Time‐lapse video microscopy of PDM‐96 expressing pLS‐mP‐NR4A2‐EGFP treated with OXPT 20 μm + #11 1 μm at time 0. Video S3. Time‐lapse video microscopy of PDM‐96 expressing pLS‐mP‐RNF43‐EGFP treated with OXPT 20 μm at time 0. Video S4. Time‐lapse video microscopy of PDM‐96 expressing pLS‐mP‐RNF43‐EGFP treated with OXPT 20 μm + #11 1 μm at time 0. File S1. Ethical documentation. [file MOL2-20-637-s001.zip › mol270152-sup-0021-FigS9.jpg]

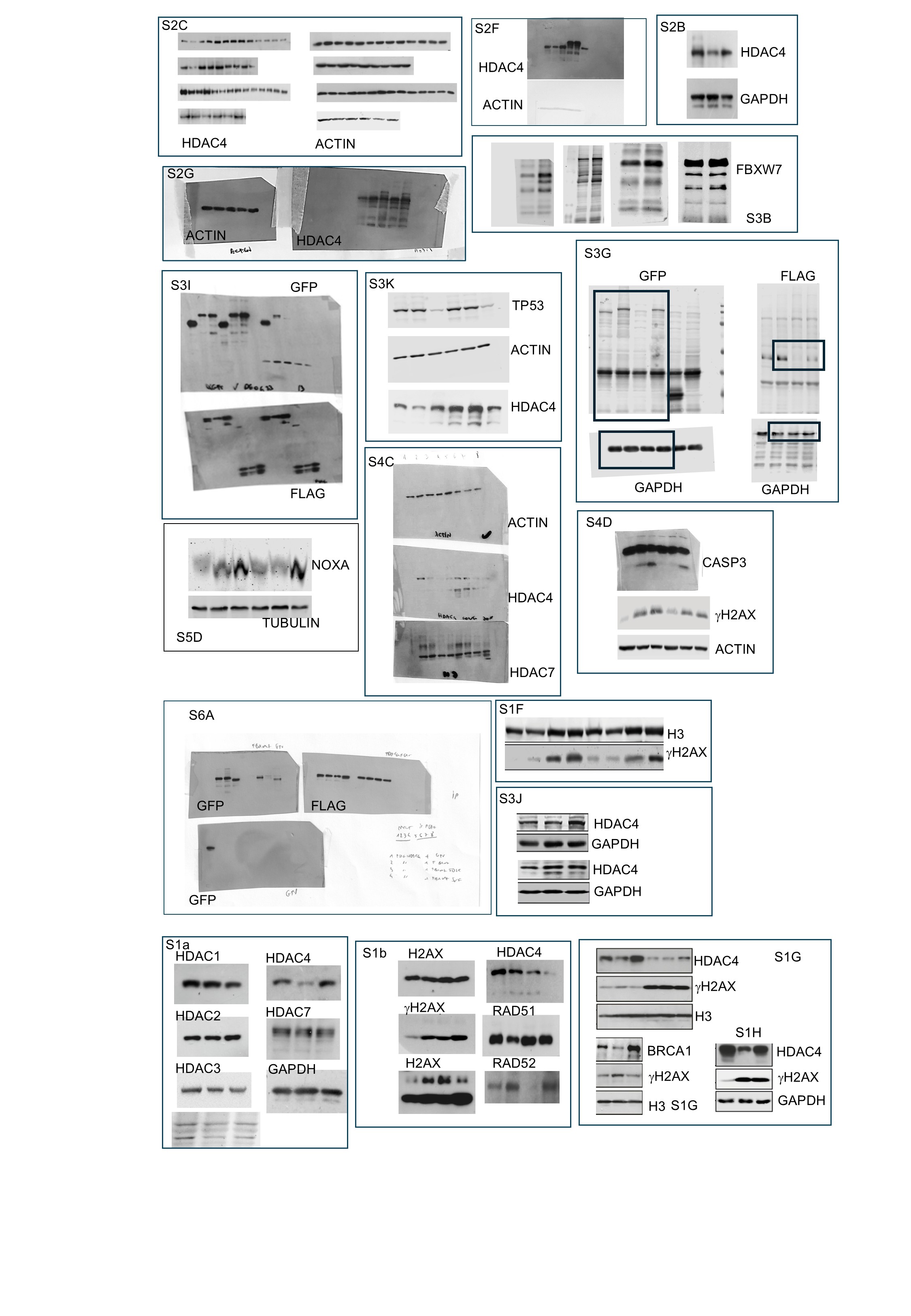

Supplement: Supplementary file 1 — Fig. S1. Inhibition of homologous recombination (HR) repair leads to a reduction in HDAC4 protein levels across various cellular contexts. Fig. S2. Identification of the E3 ligases involved in HDAC4 degradation through in silico and in vitro screenings. Fig. S3. Characterization of FBXW7−/− cells and FBXW7 R505C. Fig. S4. HDAC4 forced degradation or silencing increased OXPT cytotoxicity. Fig. S5. Identification of a signature of genes under the control of HDAC4. Fig. S6. Characterization of PDOs. Fig. S7. Characterization of the epigenetic response driven by HDAC4. Fig. S8. Dissection of the epigenetic response driven by HDAC4. Fig. S9. Original images used for the composition of the immunoblot panels in the main figures. Fig. S10. Original images used for the composition of the immunoblot panels in the supplementary figures. Table S1. Protein expression levels (z‐score) of HDAC4, HDAC5 and 365 E3 ligases available for the indicated 375 cancer cell lines of the Cancer Cell Line Encyclopedia. Table S2. Characteristics of CRC patients whose biopsies were used for the TMA. Table S3. .bed files of the SEs identified in HCT‐116 cells. Table S4. .bed files of the SEs belonging to group 1 and 2 and those directly bound by HDAC4. Table S5. Minimal signature of 116 genes associated to group 1 and 2 of SEs. Table S6. TCGA sample ID of CRC patients bearing FBXW7 LOF. Table S7. List and sequences of primers used for this study. Table S8. Raw data for in vivo experiments. Video S1. Time‐lapse video microscopy of PDM‐96 expressing pLS‐mP‐NR4A2‐EGFP treated with OXPT 20 μm at time 0. Video S2. Time‐lapse video microscopy of PDM‐96 expressing pLS‐mP‐NR4A2‐EGFP treated with OXPT 20 μm + #11 1 μm at time 0. Video S3. Time‐lapse video microscopy of PDM‐96 expressing pLS‐mP‐RNF43‐EGFP treated with OXPT 20 μm at time 0. Video S4. Time‐lapse video microscopy of PDM‐96 expressing pLS‐mP‐RNF43‐EGFP treated with OXPT 20 μm + #11 1 μm at time 0. File S1. Ethical documentation. [file MOL2-20-637-s001.zip › mol270152-sup-0022-FigS10.jpg]
